# Mapping local variation in educational attainment across Africa

**DOI:** 10.1038/nature25761

**Published:** 2018-03-01

**Authors:** Nicholas Graetz, Joseph Friedman, Aaron Osgood-Zimmerman, Roy Burstein, Molly H. Biehl, Chloe Shields, Jonathan F. Mosser, Daniel C. Casey, Aniruddha Deshpande, Lucas Earl, Robert C. Reiner, Sarah E. Ray, Nancy Fullman, Aubrey J. Levine, Rebecca W. Stubbs, Benjamin K. Mayala, Joshua Longbottom, Annie J. Browne, Samir Bhatt, Daniel J. Weiss, Peter W. Gething, Ali H. Mokdad, Stephen S. Lim, Christopher J. L. Murray, Emmanuela Gakidou, Simon I. Hay

**Affiliations:** 1 Institute for Health Metrics and Evaluation, University of Washington, Seattle, Washington 98121, USA; 2 Big Data Institute, Li Ka Shing Centre for Health Information and Discovery, University of Oxford, Oxford OX3 7FZ, UK; 3 Department of Infectious Disease Epidemiology, Imperial College London, London SW7 2AZ, UK

## Abstract

Educational attainment for women of reproductive age is linked to reduced child and maternal mortality, lower fertility and improved reproductive health. Comparable analyses of attainment exist only at the national level, potentially obscuring patterns in subnational inequality. Evidence suggests that wide disparities between urban and rural populations exist, raising questions about where the majority of progress towards the education targets of the Sustainable Development Goals is occurring in African countries. Here we explore within-country inequalities by predicting years of schooling across five by five kilometre grids, generating estimates of average educational attainment by age and sex at subnational levels. Despite marked progress in attainment from 2000 to 2015 across Africa, substantial differences persist between locations and sexes. These differences have widened in many countries, particularly across the Sahel. These high-resolution, comparable estimates improve the ability of decision-makers to plan the precisely targeted interventions that will be necessary to deliver progress during the era of the Sustainable Development Goals.

## Abstract (web summary)

Local-level analyses show that, despite marked progress in educational attainment from 2000 to 2015 across Africa, substantial differences persist between locations and sexes that have widened in many countries.

The United Nations Educational, Scientific and Cultural Organization (UNESCO) states that the ultimate mission of the education targets in Sustainable Development Goal (SDG) 4 is to “ensure inclusive and equitable quality education and promote lifelong learning opportunities for all”^1^,^2^,^3^. This is important, because it has been shown that increasing the number of years of schooling that are completed (educational attainment), can lead to higher capital, greater social mobility and increased equity among men and women, in these and other socio-economic outcomes^1^,^2^,^4^,^5^,^6^,^7^,^8^. Educational attainment for women of reproductive age is also among the leading social determinants of health, with higher attainment being strongly associated with improved reproductive health and decreased child mortality^9^,^10^,^11^,^12^,^13^,^14^. The causal pathway between education and health is difficult to study, because randomized control trial methods are logistically challenging and ethically problematic. Observational studies controlling for other predictors of health status, such as age and income, however, indicate that even small gains in educational attainment may improve health outcomes across a wide variety of low-income contexts. Studies across diverse settings have found that increased education for women of reproductive age is associated with improved child nutrition and decreased child mortality, and this effect is consistently stronger than increases in income^15^,^16^. Importantly, a comprehensive multi-level study found that increases in average attainment in communities are associated with improved survival for infants born to all women in that community, regardless of their own educational attainment or income^17^. This is consistent with research on health behaviours, showing that less-educated women model health behaviours on those of their broader community^18^. These improved health outcomes have also been shown through increased use of prenatal care, greater adherence to treatment regimens and increased contraception use^9^,^12^,^19^,^20^. Despite these clear benefits, international aid for basic education has been deprioritized as a proportion of total aid expenditure every year since 2010^21^.

## Precision public health and education

SDG 4 focuses on the reduction of inequalities in education on the basis of factors such as wealth, sex and location^1^,^2^,^22^. In addition, UNESCO’s agenda for reforming education access in developing countries is itself centred around equity^22^,^23^. Global health efforts have included substantial investments in the use of data to guide interventions that will benefit populations more efficiently and increase equity in outcomes, a strategy that has been termed precision public health^24^. The same paradigm should be extended to the social determinants of health that must be addressed for progress to be sustained. Therefore, although comparable indicators of educational attainment exist at the national level, it is increasingly important to measure subnational variation.

While past studies have assessed subnational variation in attainment for specific African countries^25^,^26^, to our knowledge no comprehensive and comparable set of estimates exist for the continent. Here we build a precisely geolocated database of 173 unique census and survey sources containing information on educational attainment (see Supplementary Figs 1–4 and Supplementary Table 2 for information on data type, coverage and source). We estimate the average number of years of attainment for women of reproductive age (15–49) across a grid of 5 × 5 km across 51 countries in Africa from 2000 to 2015. We also estimate attainment for 20–24-year-old women to more closely identify changes over time. Finally, we construct equivalent models for men to examine differences between the sexes at the same local level. We use recently developed Bayesian spatiotemporal methods^27^,^28^,^29^ for the analysis of this dataset, leveraging the high-resolution spatial and temporal information from these data. The estimates produced by these models enable comparisons of subnational regions. We focus on geographical inequality at the 5 × 5-km or local level to explore the subnational distribution of educational attainment, for the following reasons. First, data are increasingly geolocated to specific communities, and advances in Bayesian model-based geostatistics enable the modelling of these precise space–time covariance structures. Second, through the increasing availability of satellite imagery and other geospatial modelling endeavours, we have built a collection of covariates at the 5 × 5-km scale that are included in this predictive modelling framework. These are mostly available at only the community level, but allow us to predict outside of our data to estimate mean educational attainment and its uncertainty across all of Africa as a guide for policy formulation and intervention targeting. The utility of community-level and individual-level measurements is discussed in the Supplementary Discussion.

## Persistent differences in educational attainment

We used various validation strategies to assess the fit of our models. Across Africa, we use out-of-sample cross-validation to demonstrate that our models have low root mean square errors, low absolute errors, well-calibrated coverages and high concordance with existing small-area estimates (see Supplementary Figs 12–28, Supplementary Tables 8–23). Estimates of mean years of educational attainment for men and women aged 15–49 and 20–24 are shown in Fig. 1a–d and Fig. 2a–d, respectively. These summaries show geographical disparities across Africa, with persistently low levels of attainment across the Sahel region, particularly in northern Nigeria, South Sudan and northern Kenya. In 2015, Ekiti state had the highest mean attainment in Nigeria among women of reproductive age, 11.3 years (95% uncertainty interval, 10.7–11.9) years, whereas many states in the northern region had averages below two years: Kebbi, 1.6 years (1.0–2.1); Yobe, 1.7 years (1.2–2.3); Sokoto, 1.5 years (1.0–2.1); and Zamfara, 1.6 years (1.1–2.2). For the same age range in Kenya, Nairobi province had the highest average attainment, 11.4 years (10.5–12.4), whereas the more rural North Eastern province had an average of 2.1 years (1.3–3.0). The lowest four regions across all of Africa had averages of less than 0.5 years, and all were rural regions in Chad: Daraba (0.5; 0.1–1.2), Kanem (0.4; 0.1–0.9), Barl El Gazal (0.4; 0.1–0.8) and Lac (0.4; 0.1–0.9). All outputs of these analyses at the national, first administrative subdivision (for example, state), second administrative subdivision (for example, district) and 5 × 5-km levels are publicly available from the Global Health Data Exchange (http://ghdx.healthdata.org/record/africa-educational-attainment-geospatial-estimates-2000-2015) and via bespoke data visualization tools (https://vizhub.healthdata.org/lbd/education).

Marked changes were observed over time when focusing on the 20–24 age range (Fig. 2a–d), with particular improvement observed in urban centres between 2000 and 2015 in Nigeria, Kenya, Ghana, Sudan and South Africa. Several populous urban states in Nigeria showed significant gains in average attainment for women since 2000, such as Abuja state, where attainment increased from 6.0 (4.7–7.2) to 9.7 years (9.0–10.5). In Ghana, the most highly educated urban regions in the southern part of the country demonstrated moderate increases in average attainment for women aged 20–24, such as Ashanti region, where attainment improved from 7.4 (6.9–7.9) to 9.9 years (9.5–10.4). Additionally, Ghana stands out in Western Africa for its improvements in more rural regions, for example, in the Northern region attainment improved from 1.8 (1.4–2.2) to 5.2 years (4.8–5.7) since 2000.

## Implications for international goals

An explicit goal of SDG 4 is to eliminate sex-associated disparities across all levels of education by 2030^30^. We illustrate the gap in mean years of attainment between men and women for both age ranges (Figs 1e, f and 2e, f). Average attainment for men was significantly higher across the Sahel and Central Africa, particularly in the northern regions of Nigeria and Kenya that had very low levels of education in women of reproductive age (see Fig. 3). Here we use ‘significantly’ to refer to areas where 95% of the difference between Bayesian posterior predictive distributions was above zero (see Supplementary Information). These regions showed even stronger differences in the 20–24 age range, for which in some regions attainment in males was more than four years higher than in females (see Extended Data Fig. 1). Across states in 2015, we observed the largest difference in attainment by sex in the Kabia state of Chad, where men had achieved 5.8 more years (4.0–7.8) than women. In terms of statistical significance, 64 out of 77 states in Benin (representing 86% of the national population) had higher levels of attainment in males than females. The same was true for all districts within Sierra Leone, Guinea, Guinea-Bissau and Togo. By contrast, average attainment trended towards higher levels for women across much of southern Africa in 2015; however, this difference was never significant. We observed no significant differences by sex for any district within South Africa, Botswana, Zimbabwe, Rwanda and others.

**Figure 4:**
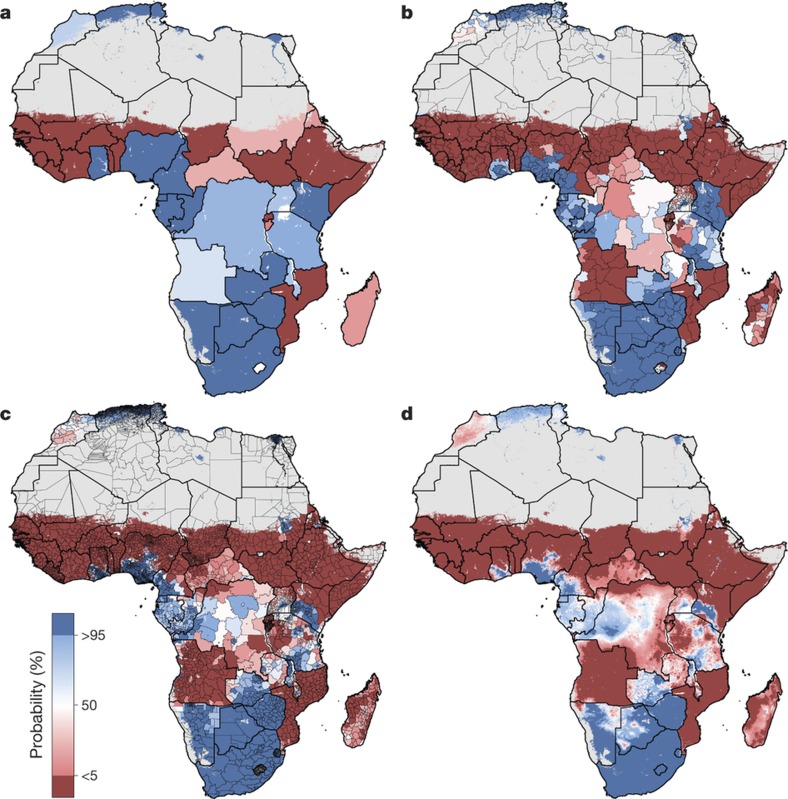
Probability that average educational attainment is greater than six years in 2015 among women of reproductive age (15–49). **a**–**d**, Probabilities at the pixel level (**d**) were aggregated using 5 × 5-km resolution population data to the district level (**c**), province level (**b**) and national level (**a**). Maps reflect administrative boundaries, land cover, lakes and population; pixels with fewer than ten people per 1 × 1 km and classified as ‘barren or sparsely vegetated’ are coloured in grey^32^,^36^,^37^,^38^,^39^,^40^.

We further examined these trends in educational opportunity by applying a threshold for attainment. UNESCO defines basic education as completing the first nine years of formal schooling, including primary education (1–6 years of schooling) and lower secondary (7–9 years of schooling)^31^. The mean of 1,000 realizations of our full model is shown in Figs 1, 2. The Bayesian modelling framework that we used enables probabilistic inferences to be made about the likelihood that such targets have been met, on the basis of the confidence of the predictions (see Supplementary Information). In Figure 4, we illustrate the probability of average attainment being above six years in 2015 for women of reproductive age, or the equivalent of completing primary education (see Extended Data Fig. 2 for women aged 20–24). Despite SDG 4 not containing specific targets on years of attainment, this threshold was selected to highlight how substantial work remains in order to achieve even basic levels of education in many subnational regions within Africa.

We use high-resolution population data to aggregate these probabilities to different administrative levels for increased use in policy development and targeted intervention strategies, as well as to demonstrate the value of geospatial estimation for showing disparities within countries^32^. For instance, at the national level, the average woman of reproductive age in Nigeria has completed primary school in 2015. At lower geographical levels, however, these probabilities ranged from almost 0 to 100% of the population depending on the district or grid cells within the district (Fig. 3). Across Africa, many areas had averages that we could reasonably conclude were less than primary school completion (less than 5% probability of being greater than six years), but others were less certain. These regions may be less certain because our estimates were very close to six years, or because our estimates had wide uncertainty intervals (see Supplementary Information). Using the precision public health paradigm, these results have important implications for investment in education. Areas that were very unlikely (less than 5%) to be achieving primary school completion in 2015 should have investment aimed at improved access to basic education (examples of such measures are discussed in the Supplementary Discussion). Many areas with higher uncertainties probably not only have very low averages, but also require increased data collection efforts. This echoes the call in precision public health to invest in quality data at the local level to target interventions most equitably and efficiently^24^.

## Discussion, limitations and future work

This study represents a notable application of Bayesian geostatistical methods in a comprehensive, geolocated dataset to model educational attainment with refined spatial and temporal resolution. Our estimates show that although attainment has generally improved for women of reproductive age in Africa since 2000, these gains have now stagnated in many subnational regions. We also demonstrate that in 2015, gaps remain in attainment between the sexes in many areas across Africa; these gaps were relatively stable over time. These findings suggest that both men and women are experiencing progress in educational attainment, but the achievement of greater equity by sex remains out of reach for much of Africa.

Geographical inequality is only one form of inequality that can be used to investigate disparities below the national level. While our framework allows us to explore geographical differences at a refined spatial level, there are many other dimensions that contribute to observed population inequities, such as social stratification by race, ethnicity or wealth (see Supplementary Discussion for limitations). Although further work is needed to explore additional forms of inequality, this predictive analysis has immediate relevance for policy development. First, our analysis maps a human capital indicator across Africa that is particularly relevant for the evolving global development agenda^33^. Second, and even more importantly, we are specifically considering educational attainment in women of reproductive age (and gender disparities in education) as a critical social determinant of maternal and thus child health^9^,^10^,^11^,^12^,^13^,^14^.

Given the intersection between educational attainment for women of reproductive age and maternal and child health targets^34^,^35^, these results have important implications for targeted investment to improve entrenched geographical and sex disparities. Communities with low education levels for women may be more likely to fail in public health interventions aimed at increasing prenatal care utilization, treatment adherence or contraception use^9^,^12^,^19^,^20^. Targeting precision health interventions without considering the landscape of human capital indexed by educational attainment poses sustainability risks, such as unrealistic assumptions about care-seeking behaviour and retention. In addition to the implications for health intervention, the global health agenda must also consider education and improved attainment as a goal itself in building sustainable, healthy populations.

Clearly the ultimate goal of SDG 4 extends beyond attainment to the quality of education. Nevertheless, as the global policy dialogue shifts to focusing on learning outcomes (see Supplementary Discussion), our results directly identify where gaps in basic education persist. These results can be used to improve accountability in need-based investment strategies from the national to local level. For communities which we have identified as having very low attainment, localized information can help to elucidate the drivers of low attendance and inform effective investment strategies.

Improving educational attainment among women of reproductive age has cross-cutting benefits for the SDG targets related to maternal and child health. This approach demonstrates the benefits of leveraging spatial information for modelling of human capital indicators in which data are correlated across space and time. This study emphasizes how documenting national-level trends in attainment masks pronounced variation across subnational areas. Despite progress, these findings suggest that large areas in sub-Saharan Africa still lag in meeting basic education targets, especially for women. In order to deliver on the promise of inclusive and equitable education for all^3^, it is critical for investments in education to be informed by locally relevant information so that no community is left behind.

## Methods

### Overview

Our study follows the Guidelines for Accurate and Transparent Health Estimates Reporting (GATHER). Using a Bayesian model-based geostatistical framework and synthesizing geolocated data from 173 household and census datasets, this analysis provides 5 × 5-km estimates of mean years of education for women of reproductive age (15–49), women aged 20–24, and equivalent male age-bins between 2000–2015 in Africa. This includes 48 countries in mainland Africa, as well as islands for which we had survey data, including Madagascar, Comoros, and São Tomé and Príncipe. We did not estimate for Mauritius, Seychelles or Cape Verde, as no available survey data could be sourced. Analytical steps are described below and additional detail can be found in the Supplementary Information.

### Data

We compiled a database of 173 survey and census datasets in Africa that contained geocoding of subnational administrative boundaries or precise coordinates for sampled clusters. These included datasets from the Demographic and Health Surveys (DHS), Multiple Indicator Cluster Surveys (MICS) and Integrated Public Use Microdata Series (IPUMS)^41^,^42^,^43^ (see Supplementary Table 2). We extracted demographic, education and sample design variables. The coding of educational attainment varies across survey families. In many surveys, respondents can indicate their level of attainment on a continuous year scale. In others, respondents may only have several aggregate categories such as ‘Secondary completion’, ‘Primary completion’, or ‘less than primary’. When all that is known is that an individual completed a particular level of education, but it is not known if they continued onto the next level, a theoretical level of completion must be assigned to the individual in order to estimate summary statistics for the population such as mean years of educational attainment. For example, if the option ‘Primary completion’ (6 years) is followed by ‘Secondary completion’ (12 years), it can be assumed that an individual who only selects the former has attained between 6 and 12 years of education. In previous literature examining trends in mean years of education, the assumption is made that all of these individuals have 6 years, or sometimes the midpoint of the feasible range (9)^44^,^45^. Trends in the single-year data demonstrate that this assumption introduces compositional bias in the estimation of attainment trends over time and space, as differences in true drop-out patterns or binning schema could lead to biased mean estimates.

For this analysis, we used a recently developed method that selects a training subset of similar surveys across time and space to estimate the true single-year distribution of binned datasets (J.F., N.G. & E.G., manuscript in preparation). This algorithmic approach markedly reduces bias in summary statistics estimated from datasets with binned coding schemes. The years in all coding schemes were mapped to the country- and year-specific references in the UNESCO International Standard Classification of Education (ISCED) for comparability^46^. We used a top coding of 18 years on all data; this is a common threshold in many surveys that have a cap and it is reasonable to assume that the importance of education for health outcomes (and other related SDGs) greatly decreases after what is the equivalent of 2 to 3 years of graduate education in most systems.

Data were aggregated to mean years for women of reproductive age (15–49) to measure progress towards the SDG 4 target^2^. A subset of the data for a smaller age range of women aged 20–24 was also examined to track temporal shifts as well as the effects of large educational initiatives in Africa since 2000. Equivalent age-bins were aggregated for males in order to examine differences in mean years of attainment by sex. Where precise coordinates were available, data were aggregated to mean years at a specific latitude and longitude assuming a simple random sample, as the cluster is the primary sampling unit for the stratified design of all DHS and MICS surveys. Where only geography information was available at the level of administrative units, data were aggregated according to their sample design. For aggregation to administrative units for which the survey was not sampled to be representative, design effects were re-estimated using a package for analysing complex survey data in R^47^.

### Spatial covariates

In order to leverage strength from locations with observations to the entire spatiotemporal domain, we compiled several 5 × 5-km raster layers of possible socio-economic and environmental correlates of education in Africa (see Supplementary Table 3 and Supplementary Fig. 5). Acquisition of temporally dynamic datasets, where possible, was prioritized in order to best match our observations and thus predict the changing dynamics of educational attainment. Of the 29 covariates included, 23 were temporally dynamic. The remaining six covariate layers were temporally static, and were applied uniformly across all modelling years. More information, including plots of all covariates, can be found in the Supplementary Information.

Our primary goal is to provide educational attainment predictions across the African continent at a high resolution and we have used methods to provide the best out-of-sample predictive performance at the expense of inferential understanding. In order to select covariates and capture possible nonlinear effects and complex interactions between them, an ensemble covariate modelling method was implemented^48^. For each region three sub-models were fit to our dataset using all of our covariate data as explanatory predictors: generalized additive models, boosted regression trees and lasso regression. Each sub-model was fit using fivefold cross-validation to avoid overfitting and the out-of-sample predictions from across the five holdouts are compiled into a single comprehensive set of predictions from that model. Additionally, the same sub-models were also run using 100% of the data and a full set of in-sample predictions were created. The five sets of out-of-sample sub-model predictions were fed into the full geostatistical model as the explanatory covariates when performing the model fit. The in-sample predictions from the sub-models were used as the covariates when generating predictions using the fitted full geostatistical model. This methodology maximizes out-of-sample predictive performance at the expense of no longer being able to provide statistical inferences on causality. A recent study has shown that this ensemble approach can improve predictive validity by up to 25% over an individual model^48^. More details on this approach can be found in the Supplementary Information.

### Analysis

#### Geostatistical model

Gaussian data are modelled within a Bayesian hierarchical modelling framework using a spatially and temporally explicit hierarchical generalized linear regression model to fit mean years of education attainment in five regions in Africa as defined in the Global Burden of Diseases, Injuries, and Risk Factors (GBD) study^49^ (‘Northern’, ‘Western’, ‘Southern’, ‘Central’ and ‘Eastern’; see Extended Data Fig. 3). GBD study design sought to create regions on the basis of two primary criteria: epidemiological homogeneity and geographical contiguity^49^. For each GBD region, we approximated the posterior distribution of our Bayesian model:
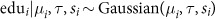

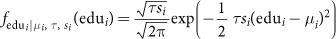

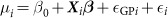

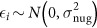

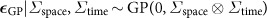

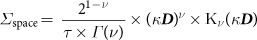

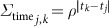
We model the mean years of attainment at cluster *i* as Gaussian data given precision *τ* and a fixed scaling parameter *s*_*i*_. We use the sample size in each cluster as our scaling parameter. We have suppressed the notation, but the means (edu_*i*_), scaling parameters (*s*_*i*_), predictions from the three submodels (***X***_*i*_), and residual terms (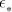) are all indexed at a space–time coordinate. The means (edu_*i*_) represent an individual’s expected educational attainment given that they live at that particular location. Mean attainment was modelled as a linear combination of the three sub-models (GAM, BRT and lasso), ***X***_*i*_, a correlated spatiotemporal error term, 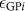, and an independent nugget effect,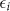. Coefficients, ***β***, on the sub-models represent their respective predictive weighting on the mean, while the joint error term, 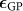, accounts for residual spatiotemporal autocorrelation between individual data points that remains after accounting for the predictive effect of the sub-model covariates and the nugget, 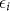, is an independent error term. The residuals, 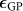, are modelled as three-dimensional Gaussian processes in space–time centred at zero and with a covariance matrix constructed from a Kroenecker product of spatial and temporal covariance kernels. The spatial covariance, *Σ*_space_, is modelled using an isotropic and stationary Matérn function^50^, and temporal covariance, *Σ*_time_, as an annual autoregressive (AR1) function over the 16 years represented in the model. This approach leveraged the data’s residual correlation structure to more accurately predict attainment estimates for locations with no data, while also propagating the dependence in the data through to uncertainty estimates^51^. The posterior distributions were fit using computationally efficient and accurate approximations in R INLA (integrated nested Laplace approximation) with the stochastic partial differential equations approximation to the Gaussian process residuals^52^. Pixel-level uncertainty intervals were generated from 1,000 draws (that is, statistically plausible candidate maps)^53^ created from the posterior-estimated distributions of modelled parameters.

To transform pixel level estimates into a range of information useful to a wide constituency of potential users, these estimates were aggregated from the 1,000 candidate maps up to district, provincial and national levels using 5 × 5-km population data^32^. This aggregation also enabled the calibration of estimates to national GBD estimates for 2000, 2005, 2010 and 2015. This was achieved by calculating the ratio of the posterior mean national-level estimate from each candidate map draw in the analysis to the posterior mean national estimates from GBD, and then multiplying each cell in the posterior sample by this ratio. This method also enabled the incorporation of the calibration into the pixel level uncertainties and thus to the uncertainties at the different levels of aggregation. The median raking factors for women aged 15–49, men aged 15–49, women aged 20–24 and men aged 20–24 were 0.926 (interquartile range (IQR): 0.794–1.084), 0.895 (IQR: 0.761–1.012), 1.036 (IQR: 0.798–1.031) and 1.053 (IQR: 0.861–1.233), respectively, indicating close agreement with GBD estimates. Scatter plots comparing national level estimates from this analysis with GBD estimates can be found in Supplementary Figs 24–27.

Although the model can predict at all locations covered by available raster covariates, all final model outputs for which land cover was classified as ‘barren or sparsely vegetated’ were masked, on the basis of the most recently available MODIS satellite data (2013), as well as areas where the total population density was less than ten individuals per 1 × 1-km pixel in 2015. This step has led to improved understanding when communicating with data specialists and policy makers.

#### Model validation

Models were validated using spatially stratified fivefold out-of-sample cross-validation. In order to offer a more stringent analysis by respecting some of the spatial correlations in the data, holdout sets were created by combining sets of spatially contiguous data. Validation was performed by calculating bias (mean error), total variance (root-mean-square error) and 95% data coverage within prediction intervals, and correlation between observed data and predictions. All validation metrics were calculated on the out-of-sample predictions from the fivefold cross-validation. Where possible, estimates from these models were compared against other existing estimates. Furthermore, measurements of spatial and temporal autocorrelation pre- and post-modelling were examined to verify correct recognition, fitting and accounting for the complex spatiotemporal correlation structure of the data. All validation procedures and corresponding results are provided in the Supplementary Information.

### Code availability

All code used for these analyses is available online at http://ghdx.healthdata.org/record/africa-educational-attainment-geospatial-estimates-2000-2015.

### Data availability

The findings of this study are supported by data that are available from public online repositories, data that are publicly available upon request of the data provider and data that are not publicly available because of restrictions by the data provider, which were used under license for the current study, but may be available from the authors upon reasonable request and permission of the data provider. A detailed table of data sources and availability can be found in Supplementary Table 2.

Administrative boundaries were retrieved from the Global Administrative Unit Layers (GAUL) dataset, implemented by FAO within the CountrySTAT and Agricultural Market Information System (AMIS) projects^36^. Land cover was retrieved from the online Data Pool, courtesy of the NASA EOSDIS Land Processes Distributed Active Archive Center (LP DAAC), USGS/Earth Resources Observation and Science (EROS) Center, Sioux Falls, South Dakota^37^. Lakes were retrieved from the Global Lakes and Wetlands Database (GLWD), courtesy of the World Wildlife Fund and the Center for Environmental Systems Research, University of Kassel^38^,^39^. Populations were retrieved from WorldPop^32^,^40^.

## Supplementary Material

Life Sciences Reporting Summary

Supplementary Information This file contains Supplementary Text and Data, Supplementary Figures 1-34, Supplementary Tables 1-24 and Supplementary References – see contents pages for details.

## Figures and Tables

**Figure 1:**
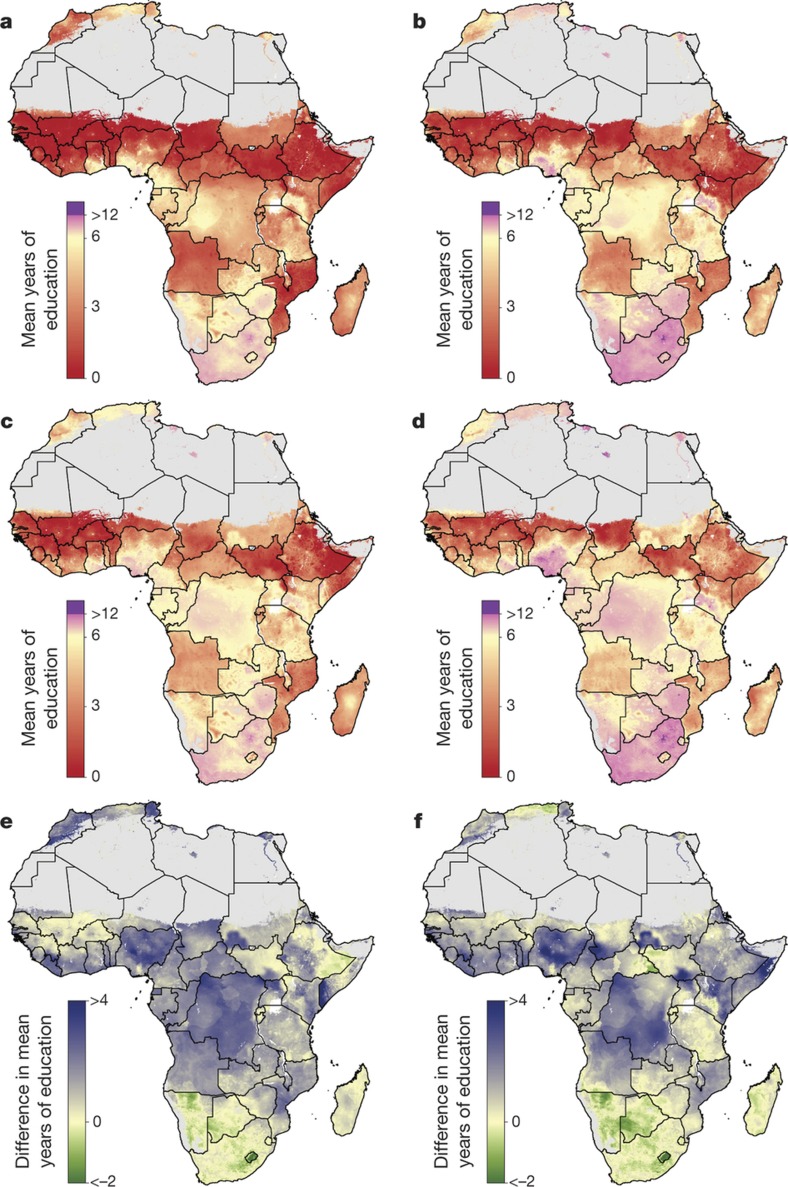
Average educational attainment for and absolute difference between women and men aged 15–49 in 2000 and 2015. **a**–**d**, Average educational attainment for women (**a**, **b**) and men (**c**, **d**) aged 15–49 in 2000 (**a**, **c**) and 2015 (**b**, **d**). **e**, **f**, The absolute difference in average educational attainment between men and women aged 15–49 in 2000 (**e**) and 2015 (**f**). Maps reflect administrative boundaries, land cover, lakes and population; pixels with fewer than ten people per 1 × 1 km and classified as ‘barren or sparsely vegetated’ are coloured in grey^32^,^36^,^37^,^38^,^39^,^40^.

**Figure 2:**
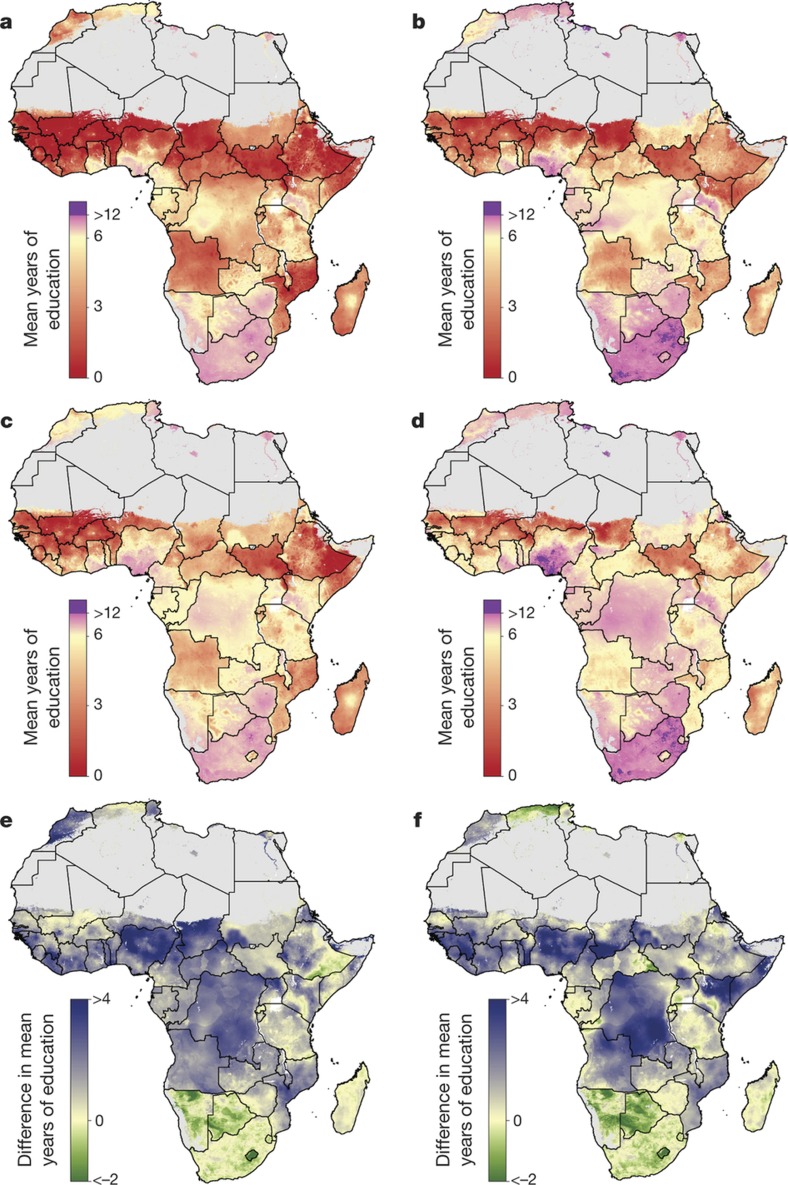
Average educational attainment for and absolute difference between women and men aged 20–24 in 2000 and 2015. **a**–**d**, Average educational attainment for women (**a**, **b**) and men (**c**, **d**) aged 20–24 in 2000 (**a**, **c**) and 2015 (**b**, **d**). **e**, **f**, The absolute difference in average educational attainment between men and women aged 20–24 in 2000 (**e**) and 2015 (**f**). Maps reflect administrative boundaries, land cover, lakes and population; pixels with fewer than ten people per 1 × 1 km and classified as ‘barren or sparsely vegetated’ are coloured in grey^32^,^36^,^37^,^38^,^39^,^40^.

**Figure 3:**
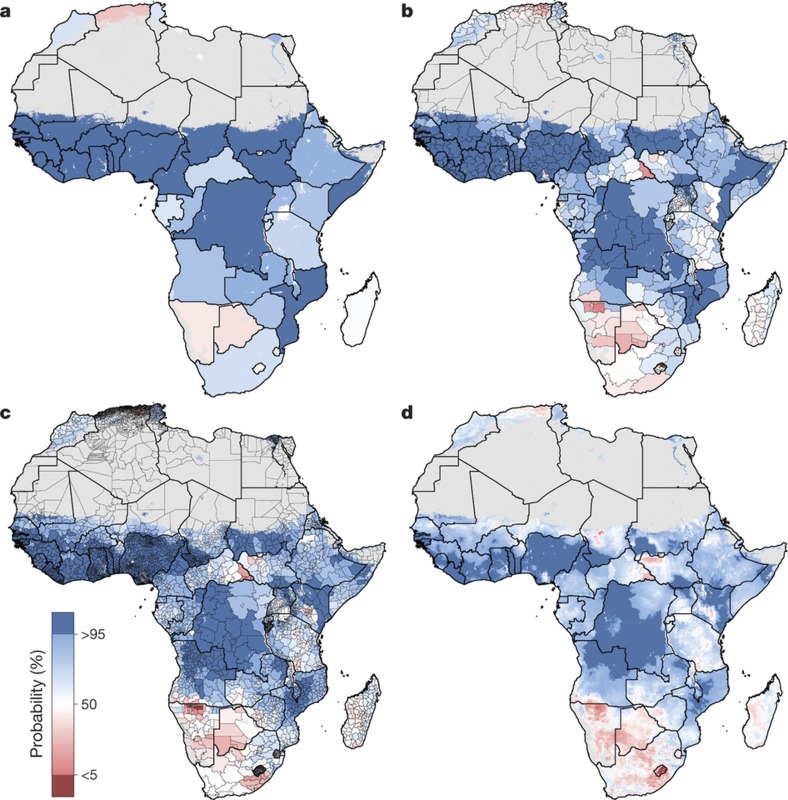
Probability that male educational attainment is greater than female educational attainment for men and women aged 15–49 in 2015. **a**–**d**, Probabilities at the pixel level (**d**) were aggregated using 5 × 5-km resolution population data to the district level (**c**), province level (**b**) and national level (**a**). Maps reflect administrative boundaries, land cover, lakes and population; pixels with fewer than ten people per 1 × 1 km and classified as ‘barren or sparsely vegetated’ are coloured in grey^32^,^36^,^37^,^38^,^39^,^40^.

**Figure 1:**
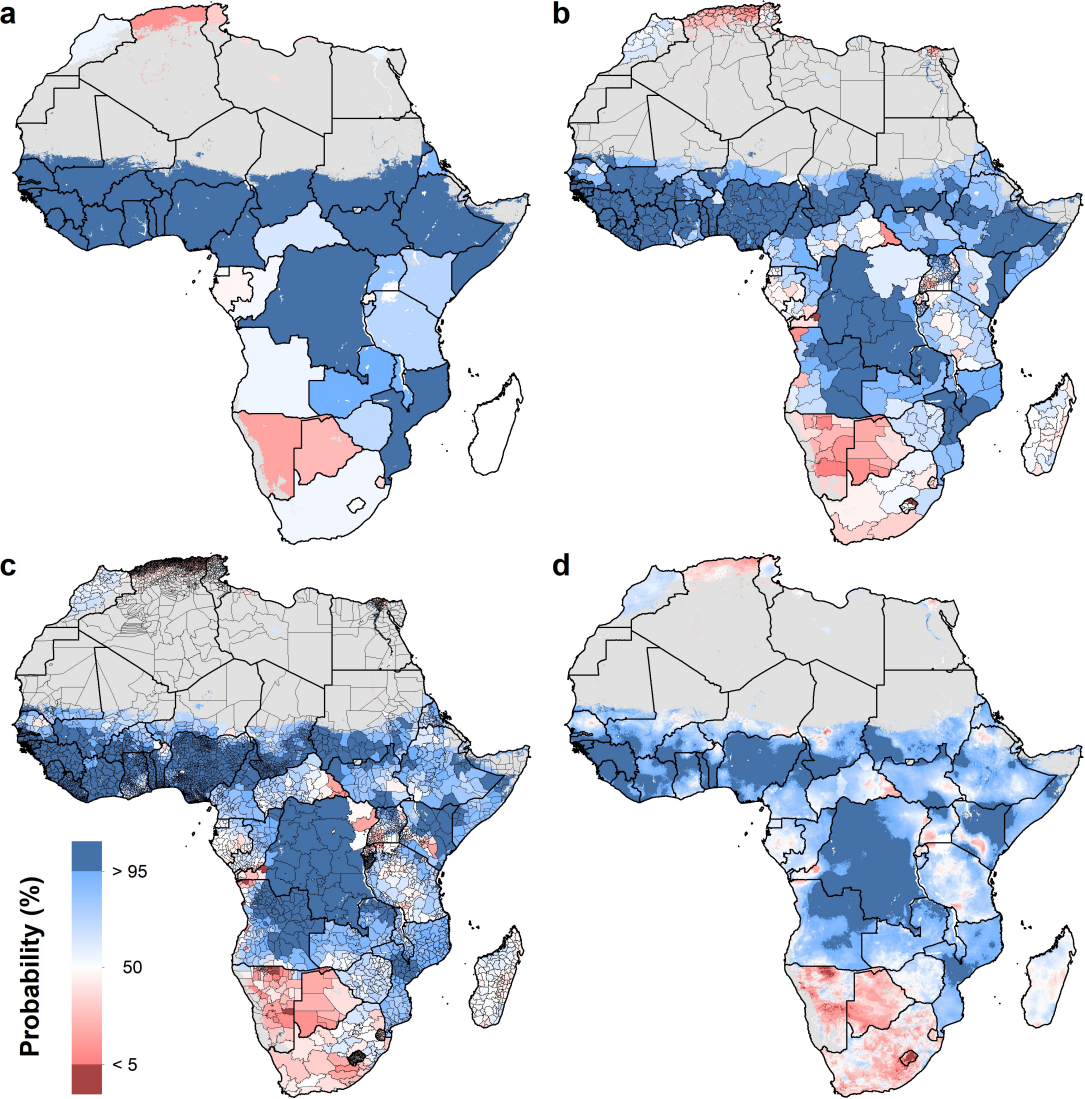
Probability that educational attainment in men is greater than attainment in women for men and women aged 20–24 in 2015 **a**–**d**, Probabilities at the pixel level (**d**) were aggregated using 5 × 5-km resolution population data to the district level (**c**), province level (**b**) and national level (A). Maps reflect administrative boundaries, land cover, lakes and population; pixels with fewer than ten people per 1 × 1 km and classified as ‘barren or sparsely vegetated’ are coloured in grey^32^,^36^,^37^,^38^,^39^,^40^.

**Figure 2:**
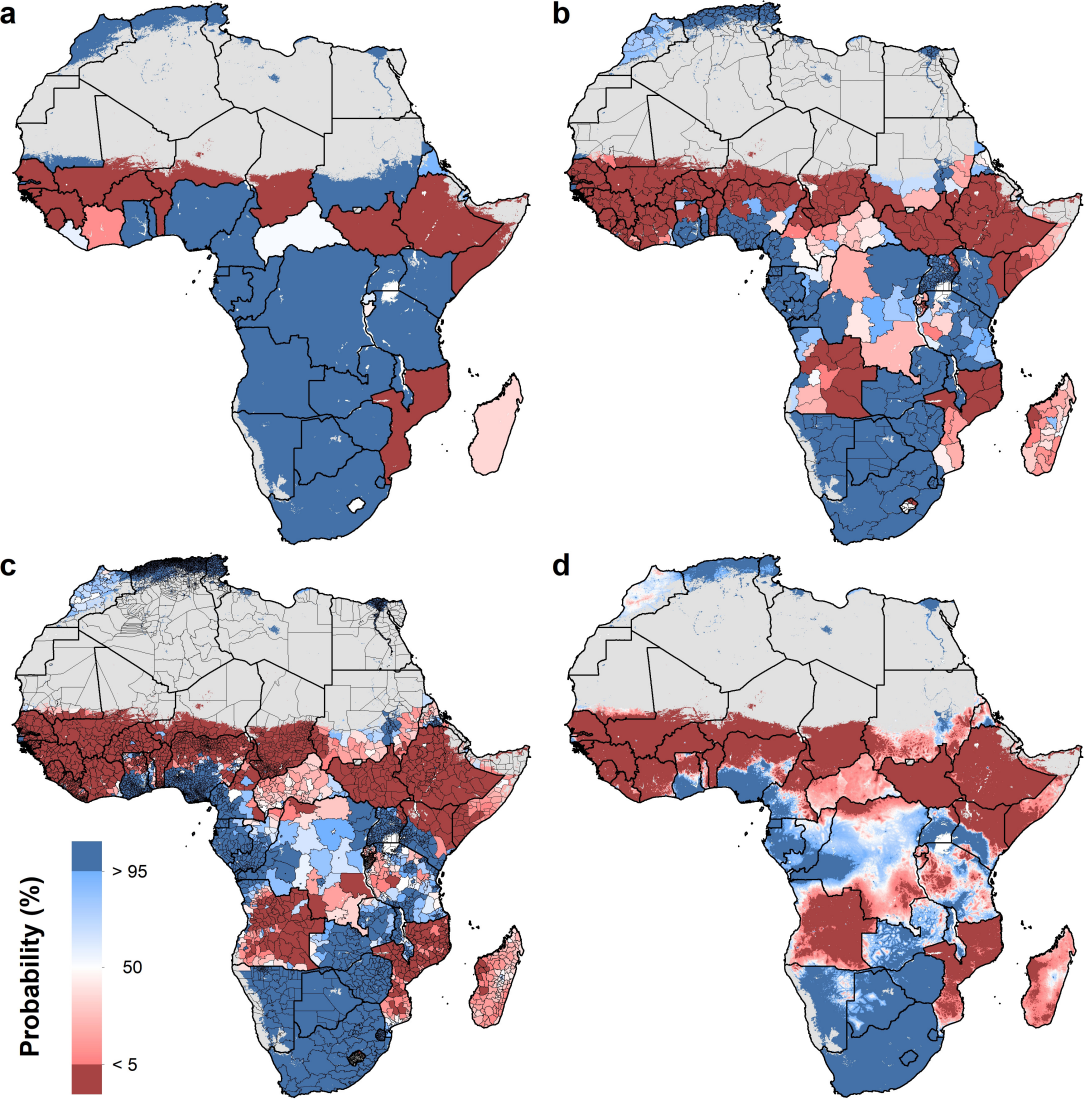
Probability that average educational attainment is greater than six years in 2015 among women aged 20–24. Probabilities at the pixel level (**d**) were aggregated using 5 × 5-km resolution population data to the district level (**c**), province level (**b**) and national level (A). Maps reflect administrative boundaries, land cover, lakes and population; pixels with fewer than ten people per 1 × 1 km and classified as ‘barren or sparsely vegetated’ are coloured in grey^32^,^36^,^37^,^38^,^39^,^40^.

**Figure 3:**
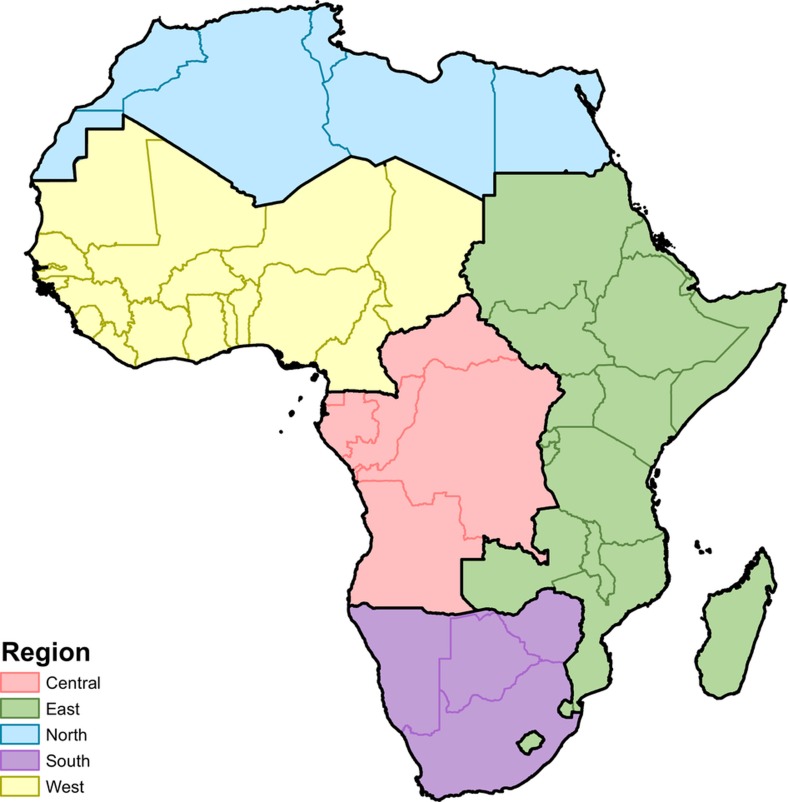
Map of modelling regions. Modelling regions were defined as the five GBD regions of Central (central sub-Saharan Africa), East (eastern sub-Saharan Africa), North (North Africa and the Middle East), South (Southern sub-Saharan Africa) and West Africa (Western sub-Saharan Africa)^54^. As this study was limited to mainland Africa and African island nations, select countries were excluded from the North Africa and Middle East region (Afghanistan, Bahrain, Iran, Iraq, Jordan, Kuwait, Lebanon, Oman, Palestinian territories, Qatar, Saudi Arabia, Syria, Turkey, United Arab Emirates and Yemen). Western Sahara was included as part of the North region. Several countries were moved to East (Lesotho and Swaziland from South, Sudan from North) to make high-income status more similar in the North and South regions.

## References

[b1] UNESCO. Global Education Monitoring Report. (UNESCO, 2016)

[b2] United Nations. Transforming our World: the 2030 Agenda for Sustainable Development. (United Nations, 2015)

[b3] United Nations. Goal 4: Ensure Inclusive and Quality Education for All and promote Lifelong Learning. (United Nations, 2016)

[b4] Klasen S. *Does Gender Inequality Reduce Growth and Development: Evidence from Cross-Country Regressions*. Working Paper Series No. 7 (World Bank, 2000)

[b5] Klasen S. Low schooling for girls, slower growth for all? Cross-country evidence on the effect of gender inequality in education on economic development. World Bank Econ. Rev. 16, 345–373 (2002)

[b6] Klasen S. & Lamanna F. The impact of gender inequality in education and employment on economic growth: new evidence for a panel of countries. Fem. Econ. 15, 91–132 (2009)

[b7] UNESCO. *Reducing Global Poverty through Universal Primary and Secondary Education*. Policy Paper 32 (UNESCO, 2017)

[b8] Abel G. J., Barakat B., Kc S. & Lutz W. Meeting the Sustainable Development Goals leads to lower world population growth. Proc. Natl Acad. Sci. USA 113, 14294–14299 (2016) 27911797 10.1073/pnas.1611386113 PMC5167193

[b9] Gakidou E., Cowling K., Lozano R. & Murray C. J. Increased educational attainment and its effect on child mortality in 175 countries between 1970 and 2009: a systematic analysis. Lancet 376, 959–974 (2010) 20851260 10.1016/S0140-6736(10)61257-3

[b10] Caldwell J. C. How is greater maternal education translated into lower child mortality? Health Transit. Rev. 4, 224–229 (1994)

[b11] Caldwell J. C. Education as a factor in mortality decline: an examination of Nigerian data. Popul. Stud. (NY) 33, 395 (1979)

[b12] Jejeebhoy S. J. Women’s Education, Autonomy, and Reproductive Behaviour: Experience from Developing Countries (Clarendon, 1995)

[b13] Desai S. & Alva S. Maternal education and child health: is there a strong causal relationship? Demography 35, 71–81 (1998) 9512911

[b14] Basu A. M. & Stephenson R. Low levels of maternal education and the proximate determinants of childhood mortality: a little learning is not a dangerous thing. Soc. Sci. Med. 60, 2011–2023 (2005) 15743650 10.1016/j.socscimed.2004.08.057

[b15] Fuchs R., Pamuk E. & Lutz W. Education or wealth: which matters more for reducing child mortality in developing countries? Vienna Yearb. Popul. Res. 8, 175–199 (2010)

[b16] Boyle M. H. . The influence of economic development level, household wealth and maternal education on child health in the developing world. Soc. Sci. Med. 63, 2242–2254 (2006) 16790308 10.1016/j.socscimed.2006.04.034

[b17] Pamuk E. R., Fuchs R. & Lutz W. Comparing relative effects of education and economic resources on infant mortality in developing countries. Popul. Dev. Rev. 37, 637–664 (2011) 22319768 10.1111/j.1728-4457.2011.00451.x

[b18] Kravdal Ø. Child mortality in India: the community-level effect of education. Popul. Stud. 58, 177–192 (2004) 10.1080/0032472042000213721 15204252

[b19] LeVine R. A., LeVine S., Schnell-Anzola B., Rowe M. L. & Dexter E. Literacy and Mothering: How Women’s Schooling Changes the Lives of the World’s Children (Oxford Univ. Press, 2012)

[b20] Makate M. & Makate C. The causal effect of increased primary schooling on child mortality in Malawi: universal primary education as a natural experiment. Soc. Sci. Med. 168, 72–83 (2016) 27639483 10.1016/j.socscimed.2016.09.003

[b21] UNESCO. Aid to Education Is Stagnating and Not Going to Countries Most in Need. (UNESCO, 2017)

[b22] UNESCO. Education 2030. *World Educ. Forum 2015* (UNESCO, 2015)

[b23] UNESCO. Is real progress being made in the equitable provision of education? #PISAresults. http://www.iiep.unesco.org/en/real-progress-being-made-equitable-provision-education-pisaresults-3915 (IIEP UNESCO, 2017)

[b24] Dowell S. F., Blazes D. & Desmond-Hellmann S. Four steps to precision public health. Nature 540, 189–191 (2016)

[b25] Bosco C. . Exploring the high-resolution mapping of gender-disaggregated development indicators. J. R. Soc. Interface 14, 20160825 (2017) 28381641 10.1098/rsif.2016.0825 PMC5414904

[b26] Roberts D. A. . Benchmarking health system performance across regions in Uganda: a systematic analysis of levels and trends in key maternal and child health interventions, 1990–2011. BMC Med. 13, 285 (2015) 26631048 10.1186/s12916-015-0518-x PMC4668680

[b27] Gething P. W. . Mapping *Plasmodium falciparum* mortality in Africa between 1990 and 2015. N. Engl. J. Med. 375, 2435–2445 (2016) 27723434 10.1056/NEJMoa1606701 PMC5484406

[b28] Bhatt S. . The effect of malaria control on *Plasmodium falciparum* in Africa between 2000 and 2015. Nature 526, 207–211 (2015) 26375008 10.1038/nature15535 PMC4820050

[b29] Diggle P. & Ribeiro P. J. Model-based Geostatistics (Springer, 2007)

[b30] United Nations. Sustainable Development Goals — United Nations (United Nations, 2015)

[b31] UNESCO. UNESCO Operational Definition Of Basic Education Thematic Framework (UNESCO, 2007)

[b32] Tatem A. J. WorldPop, open data for spatial demography. Sci. Data 4, 170004 (2017) 28140397 10.1038/sdata.2017.4 PMC5283060

[b33] The World Bank. World Bank Group President Jim Yong Kim Speech at the 2017 Annual Meetings Plenary; http://www.worldbank.org/en/news/speech/2017/10/13/wbg-president-jim-yong-kim-speech-2017-annual-meetings-plenary-session (2017)

[b34] Nilsson M., Griggs D. & Visbeck M. Policy: map the interactions between Sustainable Development Goals. Nature 534, 320–322 (2016) 27306173 10.1038/534320a

[b35] Osgood-Zimmerman A. . Mapping child growth failure in Africa between 2000 and 2015. Nature 10.1038/nature25760 (2018) PMC6346257 29493591

[b36] GeoNetwork. Global Administrative Unit Layers (GAUL); http://www.fao.org/geonetwork/srv/en/metadata.show?id=12691 (2015)

[b37] LP DAAC. Combined MODIS 5.1 dataset; available at: https://lpdaac.usgs.gov/dataset_discovery/modis/modis_products_table/mcd12q1 (accessed 1 June 2017)

[b38] World Wildlife Fund. *Global Lakes and Wetlands Database Level 3* (2004); https://www.worldwildlife.org/pages/global-lakes-and-wetlands-database (accessed 1 June 2017)

[b39] Lehner B. & Döll P. Development and validation of a global database of lakes, reservoirs and wetlands. J. Hydrol. (Amst.) 296, 1–22 (2004)

[b40] WorldPop. WorldPop dataset; available at: http://www.worldpop.org.uk/data/get_data/ (accessed 7 July 2017)

[b41] ICF. The DHS Program, Data. http://dhsprogram.com/data/ (1998)

[b42] UNICEF. Multiple Indicator Cluster Survey (MICS). https://www.unicef.org/statistics/index_24302.html (UNICEF, 2010)

[b43] Minnesota Population Center. *IPUMS International*; https://international.ipums.org/international (2015)

[b44] Barro R. J. & Lee J.-W. *International Comparisons of Educational Attainment*. NBER Working Paper No. 4349 (NBER, 1993)

[b45] Barro R. J. & Lee J.-W. *A New Data Set of Educational Attainment in the World, 1950–2010*. NBER Working Paper No. 15902 (NBER, 2010)

[b46] UNESCO. ISCED Mappings; http://uis.unesco.org/en/isced-mappings (UNESCO, 2016)

[b47] Lumley. T. Complex Surveys: A Guide to Analysis Using R (Wiley-Blackwell, 2014)

[b48] Bhatt S. . Improved prediction accuracy for disease risk mapping using Gaussian process stacked generalisation. J. R. Soc. Interface 14, 20170520 (2016) 10.1098/rsif.2017.0520 PMC5636278 28931634

[b49] Murray C. J. . GBD 2010: design, definitions, and metrics. Lancet 380, 2063–2066 (2012) 23245602 10.1016/S0140-6736(12)61899-6

[b50] Stein M. L. Interpolation of Spatial Data (Springer, 1999)

[b51] Waller L. & Carlin B. in *Handbook of* Spatial Statistics (eds Gelfand A. .) 217–243 (CRC, 2010)

[b52] Rue H., Martino S. & Chopin N. Approximate Bayesian inference for latent Gaussian models by using integrated nested Laplace approximations. J. R. Stat. Soc. B 71, 319–392 (2009)

[b53] Patil A. P., Gething P. W., Piel F. B. & Hay S. I. Bayesian geostatistics in health cartography: the perspective of malaria. Trends Parasitol. 27, 246–253 (2011) 21420361 10.1016/j.pt.2011.01.003 PMC3109552

[b54] GBD 2015 DALYs and HALE Collaborators. Global, regional, and national disability-adjusted life-years (DALYs) for 315 diseases and injuries and healthy life expectancy (HALE), 1990–2015: a systematic analysis for the Global Burden of Disease Study 2015. Lancet 388, 1603–1658 (2016) 27733283 10.1016/S0140-6736(16)31460-X PMC5388857

