# Supplementary material for: Mapping local variation in educational attainment across Africa
Source: Nature. 2018 Mar 1;555(7694):48–53. doi: 10.1038/nature25761 (PMC6346272; doi:10.1038/nature25761)
Supplement: Life Sciences Reporting Summary [file nature25761-s1.pdf]

nature research

Corresponding author(s): Simon I. Hay

☐ Initial submission ☐ Revised version ☒ Final submission

Life Sciences Reporting Summary

Nature Research wishes to improve the reproducibility of the work that we publish. This form is intended for publication with all accepted life science papers and provides structure for consistency and transparency in reporting. Every life science submission will use this form; some list items might not apply to an individual manuscript, but all fields must be completed for clarity.

For further information on the points included in this form, see Reporting Life Sciences Research. For further information on Nature Research policies, including our data availability policy, see Authors & Referees and the Editorial Policy Checklist.

Experimental design

1. Sample size

Describe how sample size was determined.	Sample size was calculated by survey/census source and was taken to be the sum of those sampled, after cleaning the data. The number of latitude-longitude referenced point data clusters, the number of areal polygons, and the total number of individuals from each source may be found in Supplementary Table 2.

2. Data exclusions

Describe any data exclusions.	Select data sources that were identified to contain years of education within the geographic area of interest were excluded for the following reasons: missing survey weights for areal data, missing gender variable, incomplete sampling (e.g., only a specific age range), or untrustworthy data (as determined by the survey administrator or by inspection). Within each source, administrative units with a sample size of one were excluded.

3. Replication

Describe whether the experimental findings were reliably reproduced.	This is an observational study using many years of survey data and could be replicated.

4. Randomization

Describe how samples/organisms/participants were allocated into experimental groups.	The data in our study predominantly comes from surveys with randomized survey designs. As an observational mapping project, there were no experimental groups.

5. Blinding

Describe whether the investigators were blinded to group allocation during data collection and/or analysis.	Blinding was not relevant to this study, as it was an observational study using survey data.

Note: all studies involving animals and/or human research participants must disclose whether blinding and randomization were used.

6. Statistical parameters

For all figures and tables that use statistical methods, confirm that the following items are present in relevant figure legends (or in the Methods section if additional space is needed).

n/a	Confirmed	
☐	☒	The exact sample size (n) for each experimental group/condition, given as a discrete number and unit of measurement (animals, litters, cultures, etc.)
☐	☒	A description of how samples were collected, noting whether measurements were taken from distinct samples or whether the same sample was measured repeatedly
☒	☐	A statement indicating how many times each experiment was replicated
☒	☐	The statistical test(s) used and whether they are one- or two-sided (note: only common tests should be described solely by name; more complex techniques should be described in the Methods section)
☐	☒	A description of any assumptions or corrections, such as an adjustment for multiple comparisons
☒	☐	The test results (e.g. P values) given as exact values whenever possible and with confidence intervals noted
☐	☒	A clear description of statistics including central tendency (e.g. median, mean) and variation (e.g. standard deviation, interquartile range)
☐	☒	Clearly defined error bars

See the web collection on statistics for biologists for further resources and guidance.

Software

Policy information about availability of computer code

7. Software

Describe the software used to analyze the data in this study.	The models were all fit using R version 3.3.2. The main statistical space-time Gaussian process regression models were fit using R-INLA version 0.0-1440400394.

For manuscripts utilizing custom algorithms or software that are central to the paper but not yet described in the published literature, software must be made available to editors and reviewers upon request. We strongly encourage code deposition in a community repository (e.g. GitHub). *Nature Methods* guidance for providing algorithms and software for publication provides further information on this topic.

Materials and reagents

Policy information about availability of materials

8. Materials availability

Indicate whether there are restrictions on availability of unique materials or if these materials are only available for distribution by a for-profit company.	No unique materials were used.

9. Antibodies

Describe the antibodies used and how they were validated for use in the system under study (i.e. assay and species).	No antibodies were used.

10. Eukaryotic cell lines

a. State the source of each eukaryotic cell line used.	No eukaryotic cell lines were used.
b. Describe the method of cell line authentication used.	No eukaryotic cell lines were used.
c. Report whether the cell lines were tested for mycoplasma contamination.	No eukaryotic cell lines were used.
d. If any of the cell lines used are listed in the database of commonly misidentified cell lines maintained by ICLAC, provide a scientific rationale for their use.	No commonly misidentified cell lines were used.

Animals and human research participants

Policy information about studies involving animals; when reporting animal research, follow the ARRIVE guidelines

11. Description of research animals

Provide details on animals and/or animal-derived materials used in the study.	No animals were used.

Policy information about studies involving human research participants

12. Description of human research participants

Describe the covariate-relevant population characteristics of the human research participants.	The study did not involve human research participants, as all data was obtained from secondary sources.
