# Supplementary material for: Mapping local variation in educational attainment across Africa
Source: Nature. 2018 Mar 1;555(7694):48–53. doi: 10.1038/nature25761 (PMC6346272; doi:10.1038/nature25761)
Supplement: Supplementary Information — This file contains Supplementary Text and Data, Supplementary Figures 1-34, Supplementary Tables 1-24 and Supplementary References – see contents pages for details. [file nature25761-s2.pdf]

doi:10.1038/nature25761

Supplementary Information

Contents

Supplementary Figures 2
Supplementary Tables 4
1.0 GATHER compliance 6
2.0 Supplementary discussion 9
2.1 Quality vs. quantity in education 9
3.0 Supplementary data 13
3.1 Data excluded from model 18
4.0 Supplementary covariates 23
5.0 Supplementary methods 30
5.1 Cluster combination and spatial integration over polygon records 30
5.2 Geostatistical model 30
5.2.1 Model geographies 30
5.2.2 Ensemble covariate modelling 31
5.2.3 Model description 31
5.2.4 Priors 33
5.2.5 Mesh construction 34
5.2.6 Model fitting and estimate generation 34
5.2.7 Model Results 35
5.3 Model validation 45
5.3.1 In-sample metrics 45
5.3.2 Metrics of predictive validity 45
5.3.3 Education (women, ages 15-49) validation metrics 46
5.3.4 Education (women, ages 20-24) validation metrics 50
5.3.5 Education (men, ages 15-49) validation metrics 54
5.3.6 Education (men, ages 20-24) validation metrics 58
5.3.7 Post estimation calibration to national estimates 62
5.3.8 Verification and comparison against other subnational education estimates 67
6.0 Supplementary references 74

doi:10.1038/nature25761

Supplementary Figures

Supplementary Figure 1. Education data availability by type and country for women ages 15-49, 2000–2015......19
Supplementary Figure 2. Education data availability by type and country for men ages 15-49, 2000–2015......20
Supplementary Figure 3. Education data availability by type and country for women ages 20-24, 2000–2015......21
Supplementary Figure 4. Education data availability by type and country for men ages 20-24, 2000–2015......22
Supplementary Figure 5. Covariates......29
Supplementary Figure 6. Finite elements mesh. 35
Supplementary Figure 7. Education (women, ages 15-49) posterior means and 95% uncertainty intervals......41
Supplementary Figure 8. Education (men, ages 15-49) posterior means and 95% uncertainty intervals......42
Supplementary Figure 9. Education (women, ages 20-24) posterior means and 95% uncertainty intervals......43
Supplementary Figure 10. Education (men, ages 20-24) posterior means and 95% uncertainty intervals......44
Supplementary Figure 11. Education (women, ages 15-49) admin 0 aggregation. Comparison of in-sample education predictions aggregated to admin 0 with 95% uncertainty intervals plotted against admin 0 aggregated data observations......47
Supplementary Figure 12. Education (women, ages 15-49) admin 1 aggregation. Comparison of in-sample education predictions aggregated to admin 1 with 95% uncertainty intervals plotted against admin 1 aggregated data observations......48
Supplementary Figure 13. Education (women, ages 15-49) admin 2 aggregation. Comparison of in-sample education predictions aggregated to admin 2 with 95% uncertainty intervals plotted against admin 2 aggregated data observations......49
Supplementary Figure 14. Education (women, ages 20-24) admin 0 aggregation. Comparison of in-sample education predictions aggregated to admin 0 with 95% uncertainty intervals plotted against admin 0 aggregated data observations......51
Supplementary Figure 15. Education (women, ages 20-24) admin 1 aggregation. Comparison of in-sample education predictions aggregated to admin 1 with 95% uncertainty intervals plotted against admin 1 aggregated data observations......52
Supplementary Figure 16. Education (women, ages 20-24) admin 2 aggregation. Comparison of in-sample education predictions aggregated to admin 2 with 95% uncertainty intervals plotted against admin 2 aggregated data observations......53
Supplementary Figure 17. Education (men, ages 15-49) admin 0 aggregation......55
Supplementary Figure 18. Education (men, ages 15-49) admin 1 aggregation......56
Supplementary Figure 19. Education (men, ages 15-49) admin 2 aggregation......57
Supplementary Figure 20. Education (men, ages 20-24) admin 0 aggregation......59
Supplementary Figure 21. Education (men, ages 20-24) admin 1 aggregation......60
Supplementary Figure 22. Education (men, ages 20-24) admin 2 aggregation......61

Supplementary Figure 23. Comparison of aggregated education (women, ages 15-49) MBG estimates to GBD 2016 education estimates. 63
Supplementary Figure 24. Comparison of aggregated education (men, ages 15-49) MBG estimates to GBD 2016 education estimates. 64
Supplementary Figure 25. Comparison of aggregated education (women, ages 20-24) MBG estimates to GBD 2016 education estimates. 65
Supplementary Figure 26. Comparison of aggregated education (men, ages 20-24) MBG estimates to GBD 2016 education estimates. 66
Supplementary Figure 27. Comparison of education (women, ages 15-49) MBG estimates aggregated to admin 1 to DHS admin 1 estimates. 67
Supplementary Figure 28. Comparison of education (men, ages 15-49) MBG estimates aggregated to admin 1 to DHS admin 1 estimates. 68
Supplementary Figure 29. Comparison of education (women, ages 20-24) MBG estimates aggregated to admin 1 to DHS admin 1 estimates. 69
Supplementary Figure 30. Comparison of education (men, ages 20-24) MBG estimates aggregated to admin 1 to DHS admin 1 estimates. 70
Supplementary Figure 31. Education (women 15-49) out-of-sample root-mean-squared-error (RMSE) and 95% coverage. 71
Supplementary Figure 32. Education (men 15-49) out-of-sample root-mean-squared-error (RMSE) and 95% coverage. 71
Supplementary Figure 33. Education (women 20-24) out-of-sample root-mean-squared-error (RMSE) and 95% coverage. 72
Supplementary Figure 34. Education (men 20-24) out-of-sample root-mean-squared-error (RMSE) and 95% coverage. 73

doi:10.1038/nature25761

Supplementary Tables

Supplementary Table 1. Guidelines for Accurate and Transparent Health Estimates Reporting (GATHER) checklist........6

Supplementary Table 2. Household surveys and censuses used in mapping........13

Supplementary Table 3. Covariates used in mapping........23

Supplementary Table 4. Spatial hyperparameter priors by region........33

Supplementary Table 5. Education (women, ages 15-49) fitted parameters........37

Supplementary Table 6. Education (male, ages 15-49) fitted parameters........38

Supplementary Table 7. Education (women, ages 20 to 24) fitted parameters........39

Supplementary Table 8. Education (men, ages 20 to 24) fitted parameters........40

Supplementary Table 9. Predictive metrics for education (women, ages 15-49) aggregated to admin 0……........46

Supplementary Table 10. Predictive metrics for education (women, ages 15-49) aggregated to admin 1…........46

Supplementary Table 11. Predictive metrics for education (women, ages 15-49) aggregated to admin 2……........46

Supplementary Table 12. Predictive metrics for education (women, ages 15-49) aggregated to holdout units........46

Supplementary Table 13. Predictive metrics for education (women, ages 20-24) aggregated to admin 0. …........50

Supplementary Table 14. Predictive metrics for education (women, ages 20-24) aggregated to admin 1. 50

Supplementary Table 15. Predictive metrics for education (women, ages 20-24) aggregated to admin 2. 50

Supplementary Table 16. Predictive metrics for education (women, ages 20-24) aggregated to holdout units........50

Supplementary Table 17. Predictive metrics for education (men, ages 15-49) aggregated to admin 0 ……….. 54

Supplementary Table 18. Predictive metrics for education (men, ages 15-49) aggregated to admin 1 ……….. 54

Supplementary Table 19. Predictive metrics for education (men, ages 15-49) aggregated to admin 2 ……….. 54

Supplementary Table 20. Predictive metrics for education (men, ages 15-49) aggregated to holdout units…… 54

Supplementary Table 21. Predictive metrics for education (men, ages 20-24) aggregated to admin 0 ………........58

Supplementary Table 22. Predictive metrics for education (men, ages 20-24) aggregated to admin 1 ………...58

Supplementary Table 23. Predictive metrics for education (men, ages 20-24) aggregated to admin 2 ……..…..58

Supplementary Table 24. Predictive metrics for education (men, ages 20-24) aggregated to holdout units …… 58

1.0 GATHER compliance

Supplementary Table 1. Guidelines for Accurate and Transparent Health Estimates Reporting (GATHER) checklist

Item #	Checklist item	Reported on page #
**Objectives and funding**		
**1**	Define the indicator(s), populations (including age, sex, and geographic entities), and time period(s) for which estimates were made.	Main text: Precision public health and education, Main text: Methods
**2**	List the funding sources for the work.	Main text: Acknowledgements
**Data Inputs**		
For all data inputs from multiple sources that are synthesized as part of the study:		
**3**	Describe how the data were identified and how the data were accessed.	Main text: Methods (Analysis)
**4**	Specify the inclusion and exclusion criteria. Identify all ad-hoc exclusions.	Main text: Methods, Supplementary Information: 3.1 Data excluded from model
**5**	Provide information on all included data sources and their main characteristics. For each data source used, report reference information or contact name/institution, population represented, data collection method, year(s) of data collection, sex and age range, diagnostic criteria or measurement method, and sample size, as relevant.	Supplementary Information: 3.0 Supplementary data
**6**	Identify and describe any categories of input data that have potentially important biases (e.g., based on characteristics listed in item 5).	Main text: Methods
For data inputs that contribute to the analysis but were not synthesized as part of the study:		
**7**	Describe and give sources for any other data inputs.	Supplementary Information: 4.0 Supplementary covariates
For all data inputs:		
**8**	Provide all data inputs in a file format from which data can be efficiently extracted (e.g., a spreadsheet rather than a PDF), including all relevant meta-data listed in item 5. For any data inputs that cannot be shared because of ethical or legal reasons, such as third-party ownership, provide a contact name or the name of the institution that retains the right to the data.	Available at http://ghdx.healthdata.org/record/africa-educational-attainment-geospatial-estimates-2000-2015<br><br>Supplementary Information: 3.0 Supplementary data

Data analysis		
**9**	Provide a conceptual overview of the data analysis method. A diagram may be helpful.	Main text: Methods (Analysis)
**10**	Provide a detailed description of all steps of the analysis, including mathematical formulae. This description should cover, as relevant, data cleaning, data pre-processing, data adjustments and weighting of data sources, and mathematical or statistical model(s).	Main text: Methods, Supplementary Information: 5.2 Geostatistical model
**11**	Describe how candidate models were evaluated and how the final model(s) were selected.	Main text: Methods (Model validation), Supplementary Information: 5.2 Geostatistical model, 5.3 Model validation
**12**	Provide the results of an evaluation of model performance, if done, as well as the results of any relevant sensitivity analysis.	Main text: Methods (Model validation), Supplementary Information: 5.2 Geostatistical model, 5.3 Model validation
**13**	Describe methods for calculating uncertainty of the estimates. State which sources of uncertainty were, and were not, accounted for in the uncertainty analysis.	Main text: Methods (Model validation), Supplementary Information: 5.2 Geostatistical model, 5.3 Model validation
**14**	State how analytic or statistical source code used to generate estimates can be accessed.	Available at http://ghdx.healthdata.org/record/africa-educational-attainment-geospatial-estimates-2000-2015
**Results and Discussion**		
**15**	Provide published estimates in a file format from which data can be efficiently extracted.	Raster files for spatial data and CSVs of estimates available at http://ghdx.healthdata.org/record/africa-educational-attainment-geospatial-estimates-2000-2015
**16**	Report a quantitative measure of the uncertainty of the estimates (e.g. uncertainty intervals).	Supplementary Information: 5.2 Geostatistical model, 5.3 Model validation
**17**	Interpret results in light of existing evidence. If updating a previous set of estimates, describe the reasons for changes in estimates.	Main text: Persistent differences in educational attainment, Implications for international goals

**18**	Discuss limitations of the estimates. Include a discussion of any modelling assumptions or data limitations that affect interpretation of the estimates.	Main text: Discussion, limitations and future work, Supplementary Information: 2.4 Limitations of a geographic perspective

2.0 Supplementary discussion

2.1 Policy implications of community-level education measurement

The past decade has seen huge increases in enrollment across sub-Saharan Africa. However, this has created a shift in international and domestic policy initiatives from school enrollment to improving quality.[1] With stagnating international aid, we argue that shifting resources away from basic attainment is premature and it is now more important than ever to identify where gaps still exist.[2] Studies have pointed out that these are typically the most marginalized rural communities, and our results certainly identify these communities even in countries that have high national average attainment.[3,4] Neglecting these communities while shifting national policies to target quality is a wasted opportunity not just for improving human capital but for achieving the child and maternal health targets of the SDGs.[5]

There is no universal solution for improving enrollment in these communities. Many rural regions of sub-Saharan Africa still lack the basic physical infrastructure and teaching workforce necessary for education delivery. But as studies have shown, the problem is typically not lack of schools and basic resources, but low attendance rates.[4,6] The drivers are diverse and vary regionally. Many interventions have found success in providing incentives for attendance, such as deworming programs or free school lunches.[7] Rural regions of Southeast Asia and Latin America have seen success in employing cluster-school systems to pool limited resources, improve efficiency and increase access.[8] Many countries have moved to eliminate fees and costs associated with primary school attendance, but in many cases these economic incentives have not been strong enough. Girls are especially at risk, as in many rural communities there are financial pressures to marry at very early ages. A World Bank report notes the immense social and economic impact of marriage before 18, which is especially prevalent in rural Niger, Ethiopia, Uganda and Nigeria.[9] In the countries examined, one in five women will have their first child before the age of 18. The analysis suggests that gains in annual welfare from the lower population growth associated with reduced child marriage leading to improved school enrollment could reach more than $500 billion annually by 2030. The World Bank also estimates that through ending child marriage and keeping young girls in school, the benefits of lower under-five mortality and malnutrition could reach more than $90 billion annually by 2030.[9]

Several organizations have offered policy solutions that can be used to address enrollment in these neglected communities. The recently released Global Education Monitoring Report (GEM) 2017/8 stresses the need for accountability in education and investment therein, especially in the context of decreasing international aid.[10] The education sector is one of the most frequently targeted by accusations of widespread corruption.[11] The World Bank has advocated for results-based financing, aligning investment incentives with improvement in desired outcomes.[12] They have also advocated for investment in pre-school programs and early child development, which can help improve a child's success once enrolled and reduce the risk of dropout.[1]

Clearly the ultimate goal of SDG4 extends beyond attainment to the quality of education. Nevertheless, as the global policy dialogue shifts to focusing on learning outcomes (see Supplementary Discussion), our results identify directly where gaps to basic education persist. These results can be leveraged to improve accountability in need-based investment strategies from the national- to local-level. In communities we have identified as having very low attainment, localized information can help elucidate the drivers of low attendance and inform effective investment strategies.

2.2 Quality vs. quantity in education

As a proportion of total aid, international funding for education has dropped in priority every year since 2010, and the subset of that funding targeted at basic education has stagnated.[2,4] Research has demonstrated that aid improves quantitative indicators of education including infrastructure (i.e. schools, textbooks and teachers), enrollment, and, most significantly, attainment. There is a bias toward investing in these more easily measured outcomes rather than in indicators of higher quality, yet research has failed to demonstrate that increased enrollment and attainment always lead to improved literacy and cognitive outcomes. Within several countries, UNESCO highlights alarming statistics of persistent illiteracy in children despite high overall rates of primary schooling, demonstrating that it is a separate, challenging task to assess quality differences across different systems.[11] While literacy and other quality outcomes have been shown to mediate the positive association between education and health, the effect of attainment itself remains significant for improving maternal and child health outcomes while controlling for other known predictors.[3,13] To help explain this linkage, researchers have demonstrated across a wide variety of low-income contexts that the pervasiveness of Westernized education delivery may facilitate knowledge gains and navigation of other bureaucratically organized systems, such as health and family planning sectors.[3] In subnational areas where the current average is near zero, increasing educational attainment even two or three years may have cross-cutting benefits to SDG targets pertaining to health and gender equity. Even a few years of attainment, regardless of the quality of education received, has been associated with increased prenatal care-seeking, comprehension of public health messaging, ability to effectively articulate illness narratives to physicians, and internalization of the pupil-teacher (and by extension, patient-provider) relationship that improves trust in medical experts and uptake of treatment recommendations.[3] Because international aid has stagnated for basic education, it is more important than ever to be intentional and targeted with limited resources in order to achieve the SDG milestone of basic education for all.

2.3 Community-level vs. individual-level measurement

Average educational attainment at the 5x5 km / local-level has a significant effect on infant mortality beyond the attainment of individuals.[3,14] The purpose of our analysis is not inferential

and does not seek to provide new insight into these observational relationships between education and sustainable development and health, relationships we find to already be very convincing in the literature.[3,13–15] Rather, our analysis goes one step further by taking a purely predictive perspective to provide those working in the health and development implementation sectors with the most accurate estimates of educational attainment at a granular local level, combining all available data, covariates, and spatiotemporal modelling techniques.

The policy relevance of this predictive analysis is twofold. First, our analysis is novel in mapping a human capital indicator across Africa that is particularly relevant to the global development agenda. Second, and even more importantly, we are specifically considering educational attainment in women of reproductive age (and gender disparities in education) as a critical social determinant of health that should not be overlooked by the global health agenda. As resource allocation and implementation efforts based on precision public health continue to grow, such as interventions targeting child growth failure, it is increasingly important to recognize how the geographic distribution of mortality and disease is driven by the geographic distribution of human capital. Our analysis thus combines all available data with the most statistically robust methodology to provide unique evidence for a public health intervention paradigm. Targeting precision health interventions without considering the landscape of human capital poses large risks to the sustainability of intervention strategies (i.e., unrealistic assumptions about care-seeking behavior and retention). High resolution estimates of average, local-level human capital must be considered and used in conjunction with granular estimates of disease and mortality when striving to make sustainable programmatic decisions at a local level.

2.4 Limitations of a geographic perspective

Geographic inequality is only one form of inequality that can be used to investigate heterogeneity below the national level. While our framework allows us to explore geographic differences at a refined spatial level, there are many other dimensions that contribute to observed population inequities, such as social stratification by race, ethnicity or wealth. These dimensions may be particularly useful in parsing the landscape of educational attainment in southern sub-Saharan Africa, where countries like South Africa have large socioeconomic gaps in urban centers. Using national household surveys in several countries, the most recent Global Education Monitoring Report found that while rural poor have much lower secondary completion rates than the national average, rates among urban poor are equally low and often worse.[16] Though in the present study we use a high resolution 5x5 km grid, even this will have difficulty identifying spatial heterogeneity in extremely concentrated urban centers, especially in the absence of robust high resolution socioeconomic covariate layers and precise data coverage. Given the volume of informal settlements throughout many large cities in Africa, this highlights the need for future analyses and data collection to focus on capturing inequalities across disparate urban communities.

doi:10.1038/nature25761

3.0 Supplementary data

The data sources used to model educational attainment indicators are described below. Information on survey locations, years, source, polygons, and/or geo-positioned clusters can be found in Supplementary Table 2. Supplementary Figures 1-4 display data availability for each indicator. Reasons for data sets being excluded from analysis are detailed in Supplementary Information 3.1, Data Excluded from Model.

Supplementary Table 2. Household surveys and censuses used in mapping. Number identification (NID) can be used to locate a particular data source in the Global Health Data Exchange at http://ghdx.healthdata.org/.

Country	Survey year(s)	Source	Number Identification (NID)	Number of geo-positioned clusters	Number of polygons (areal)
Algeria	2012	UNICEF MICS	210614	0	1909
Angola	2001	UNICEF MICS	687	0	1502
Angola	2015	DHS Program	218555	625	0
Benin	2001	DHS Program	18950	247	0
Benin	2006	DHS Program	18959	0	143
Benin	2011	DHS Program	79839	746	0
Botswana	2000	UNICEF MICS	1404	0	1
Burkina Faso	1998	DHS Program	19076	208	0
Burkina Faso	2003	DHS Program	19088	397	0
Burkina Faso	2006	IPUMS	105403	0	350
Burkina Faso	2006	UNICEF MICS	1927	195	0
Burkina Faso	2010	DHS Program	19133	541	0
Burundi	2000	UNICEF MICS	1994	0	39
Burundi	2005	UNICEF MICS	1981	0	39
Burundi	2010	DHS Program	30431	376	0
Cameroon	1998	DHS Program	19198	0	552
Cameroon	2000	UNICEF MICS	2053	0	552
Cameroon	2004	DHS Program	19211	463	0
Cameroon	2005	IPUMS	105800	0	575
Cameroon	2006	UNICEF MICS	2063	0	752
Cameroon	2011	DHS Program	19274	577	0
Central African Republic	2000	UNICEF MICS	2209	0	702
Central African Republic	2006	UNICEF MICS	2223	0	475
Central African Republic	2010	UNICEF MICS	82832	0	702
Chad	2000	UNICEF MICS	2244	0	1462

Country	Survey year(s)	Source	Number Identification (NID)	Number of geo-positioned clusters	Number of polygons (areal)
Chad	2004	DHS Program	19315	0	1466
Chad	2010	UNICEF MICS	76701	0	1453
Chad	2014	DHS Program	157025	624	0
Comoros	2012	DHS Program	76850	242	0
Congo	2005	DHS Program	19391	0	406
Congo	2011	DHS Program	56151	0	406
Côte d'Ivoire	1998	DHS Program	18531	140	0
CÃ´te D'ivoire	2000	UNICEF MICS	26444	0	384
CÃ´te D'ivoire	2006	UNICEF MICS	26433	0	282
CÃ´te D'ivoire	2011	DHS Program	18533	341	0
Democratic Republic of the Congo	2001	UNICEF MICS	3161	0	2717
Democratic Republic of the Congo	2007	DHS Program	19381	293	0
Democratic Republic of the Congo	2010	UNICEF MICS	26998	0	2717
Democratic Republic of the Congo	2013	DHS Program	76878	492	0
Djibouti	2006	UNICEF MICS	3404	96	1
Egypt	2000	DHS Program	19511	997	0
Egypt	2005	DHS Program	19521	1298	0
Egypt	2006	IPUMS	35578	0	895
Egypt	2008	DHS Program	26842	1244	0
Egypt	2014	DHS Program	154897	1815	0
Eritrea	2002	DHS Program	19539	0	148
Ethiopia	2000	DHS Program	19571	533	0
Ethiopia	2005	DHS Program	19557	528	0
Ethiopia	2007	IPUMS	227133	0	1382
Ethiopia	2010	DHS Program	21301	571	0
Ethiopia	2016	DHS Program	218568	622	0
Gabon	2000	DHS Program	19579	0	297
Gabon	2012	DHS Program	76706	331	0
Gambia	2000	UNICEF MICS	3922	0	18
Gambia	2013	DHS Program	77384	0	37
Ghana	1998	DHS Program	19614	400	0
Ghana	2000	IPUMS	38508	0	329
Ghana	2003	DHS Program	19627	410	0
Ghana	2006	UNICEF MICS	4694	0	286

Country	Survey year(s)	Source	Number Identification (NID)	Number of geo-positioned clusters	Number of polygons (areal)
Ghana	2007	UNICEF MICS	160576	0	117
Ghana	2008	DHS Program	21188	404	0
Ghana	2010	IPUMS	151306	0	371
Ghana	2011	UNICEF MICS	63993	13	0
Ghana	2014	DHS Program	157027	427	0
Guinea	1999	DHS Program	19670	293	0
Guinea	2005	DHS Program	19683	291	0
Guinea	2012	DHS Program	69761	300	0
Guinea-Bissau	2000	UNICEF MICS	4808	0	39
Guinea-Bissau	2006	UNICEF MICS	4818	0	39
Kenya	1998	DHS Program	20132	530	0
Kenya	2000	UNICEF MICS	7387	883	0
Kenya	2003	DHS Program	20145	399	0
Kenya	2008	DHS Program	21365	397	0
Kenya	2014	DHS Program	157057	1591	0
Lesotho	2004	DHS Program	20167	381	0
Lesotho	2009	DHS Program	21382	395	0
Lesotho	2014	DHS Program	157058	399	0
Liberia	2006	DHS Program	20191	291	0
Liberia	2008	IPUMS	151310	0	117
Liberia	2013	DHS Program	77385	322	0
Madagascar	2003	DHS Program	20223	0	730
Madagascar	2008	DHS Program	21409	585	0
Madagascar	2012	UNICEF MICS	125594	127	0
Malawi	1998	IPUMS	40179	0	259
Malawi	2000	DHS Program	20252	559	0
Malawi	2004	DHS Program	20263	520	0
Malawi	2008	IPUMS	40186	0	260
Malawi	2010	DHS Program	21393	827	0
Malawi	2013	UNICEF MICS	161662	0	127
Mali	1998	IPUMS	40235	0	1047
Mali	2001	DHS Program	20315	399	0
Mali	2006	DHS Program	20274	405	0
Mali	2009	IPUMS	151311	0	952
Mali	2012	DHS Program	77388	413	0
Mauritania	2000	DHS Program	20322	0	770
Mauritania	2011	UNICEF MICS	152783	0	769
Morocco	2003	DHS Program	20361	480	0
Morocco	2004	IPUMS	56492	0	597
Mozambique	2003	DHS Program	20394	0	959
Mozambique	2007	IPUMS	227143	0	1024
Mozambique	2011	DHS Program	55975	609	0
Namibia	2000	DHS Program	20417	259	0

doi:10.1038/nature25761

Country	Survey year(s)	Source	Number Identification (NID)	Number of geo-positioned clusters	Number of polygons (areal)
Namibia	2006	DHS Program	20428	491	0
Namibia	2013	DHS Program	150382	549	0
Niger	1998	DHS Program	20537	268	0
Niger	2000	UNICEF MICS	9439	0	1123
Niger	2006	DHS Program	20499	0	1123
Niger	2012	DHS Program	74393	0	1123
Nigeria	2003	DHS Program	20567	360	0
Nigeria	2006	IPUMS	151312	0	1097
Nigeria	2007	IPUMS	151312	0	1097
Nigeria	2007	UNICEF MICS	9516	0	1095
Nigeria	2008	IPUMS	151313	0	1097
Nigeria	2008	DHS Program	21433	886	0
Nigeria	2009	IPUMS	151314	0	1097
Nigeria	2010	IPUMS	151317	0	1097
Nigeria	2013	DHS Program	77390	889	0
Rwanda	2000	DHS Program	20722	0	36
Rwanda	2000	UNICEF MICS	26930	0	36
Rwanda	2002	IPUMS	42432	0	35
Rwanda	2005	DHS Program	20740	456	0
Rwanda	2010	DHS Program	56040	492	0
Rwanda	2014	DHS Program	157063	492	0
Sao Tome and Principe	2000	UNICEF MICS	27055	0	3
Sao Tome and Principe	2008	DHS Program	26866	0	7
Sao Tome and Principe	2014	UNICEF MICS	214640	0	7
Senegal	2000	UNICEF MICS	27044	0	241
Senegal	2002	IPUMS	43142	0	256
Senegal	2005	DHS Program	26855	366	0
Senegal	2010	DHS Program	56063	385	0
Senegal	2012	DHS Program	111432	200	0
Senegal	2014	DHS Program	191270	0	244
Senegal	2015	DHS Program	218592	0	244
Senegal	2016	DHS Program	286772	214	0
Sierra Leone	2000	UNICEF MICS	11639	0	86
Sierra Leone	2004	IPUMS	11661	0	92
Sierra Leone	2008	DHS Program	21258	350	0
Sierra Leone	2010	UNICEF MICS	76700	0	93
Sierra Leone	2013	DHS Program	131467	435	0
Somalia	2006	UNICEF MICS	11774	0	751
Somalia	2011	UNICEF MICS	91507	509	109
South Africa	1998	DHS Program	20796	0	1610
South Africa	2001	IPUMS	43152	0	1635
South Africa	2007	IPUMS	43158	0	1632

Country	Survey year(s)	Source	Number Identification (NID)	Number of geo-positioned clusters	Number of polygons (areal)
South Africa	2011	IPUMS	227194	0	1631
South Sudan	2008	IPUMS	106548	0	762
Swaziland	2000	UNICEF MICS	12320	0	24
Swaziland	2006	DHS Program	20829	270	0
Swaziland	2010	UNICEF MICS	30325	0	24
Togo	1998	DHS Program	20909	287	0
Togo	2000	UNICEF MICS	12886	0	70
Togo	2006	UNICEF MICS	12896	0	70
Togo	2010	UNICEF MICS	40021	0	70
Togo	2013	DHS Program	77515	330	0
Tunisia	2011	UNICEF MICS	76709	0	223
Uganda	2000	DHS Program	20993	266	0
Uganda	2002	IPUMS	43328	0	261
Uganda	2006	DHS Program	21014	336	0
Uganda	2011	DHS Program	56021	400	0
United Republic of Tanzania	1999	DHS Program	20865	173	0
United Republic of Tanzania	2002	IPUMS	43212	0	1112
United Republic of Tanzania	2004	DHS Program	20875	0	1061
United Republic of Tanzania	2009	DHS Program	21331	458	0
United Republic of Tanzania	2015	DHS Program	218593	608	0
Yemen	2006	UNICEF MICS	13816	0	176
Zambia	2000	IPUMS	151325	0	895
Zambia	2001	DHS Program	21102	0	896
Zambia	2007	DHS Program	21117	319	0
Zambia	2010	IPUMS	151326	0	895
Zambia	2013	DHS Program	77516	719	0
Zimbabwe	1999	DHS Program	21151	221	0
Zimbabwe	2005	DHS Program	21163	396	0
Zimbabwe	2009	UNICEF MICS	35493	0	486
Zimbabwe	2010	DHS Program	55992	393	0
Zimbabwe	2014	UNICEF MICS	152720	0	972
Zimbabwe	2015	DHS Program	157066	400	0

3.1 Data excluded from model

Select data sources that were identified to contain years of education within the geographic area of interest were excluded for the following reasons: missing survey weights for areal data, missing gender variable, incomplete sampling (e.g., only a specific age range), or untrustworthy data (as determined by the survey administrator or by inspection). We use the term "untrustworthy data (as determined by the survey administrator or by inspection)" in reference to the exclusion of only one specific source for the education model, the 2008 South Sudan Census. This source was removed due to implausibly high educational attainment values around the capital city and expert opinion regarding the quality of the data. Within each source, administrative units with a sample size of one were excluded.

doi:10.1038/nature25761

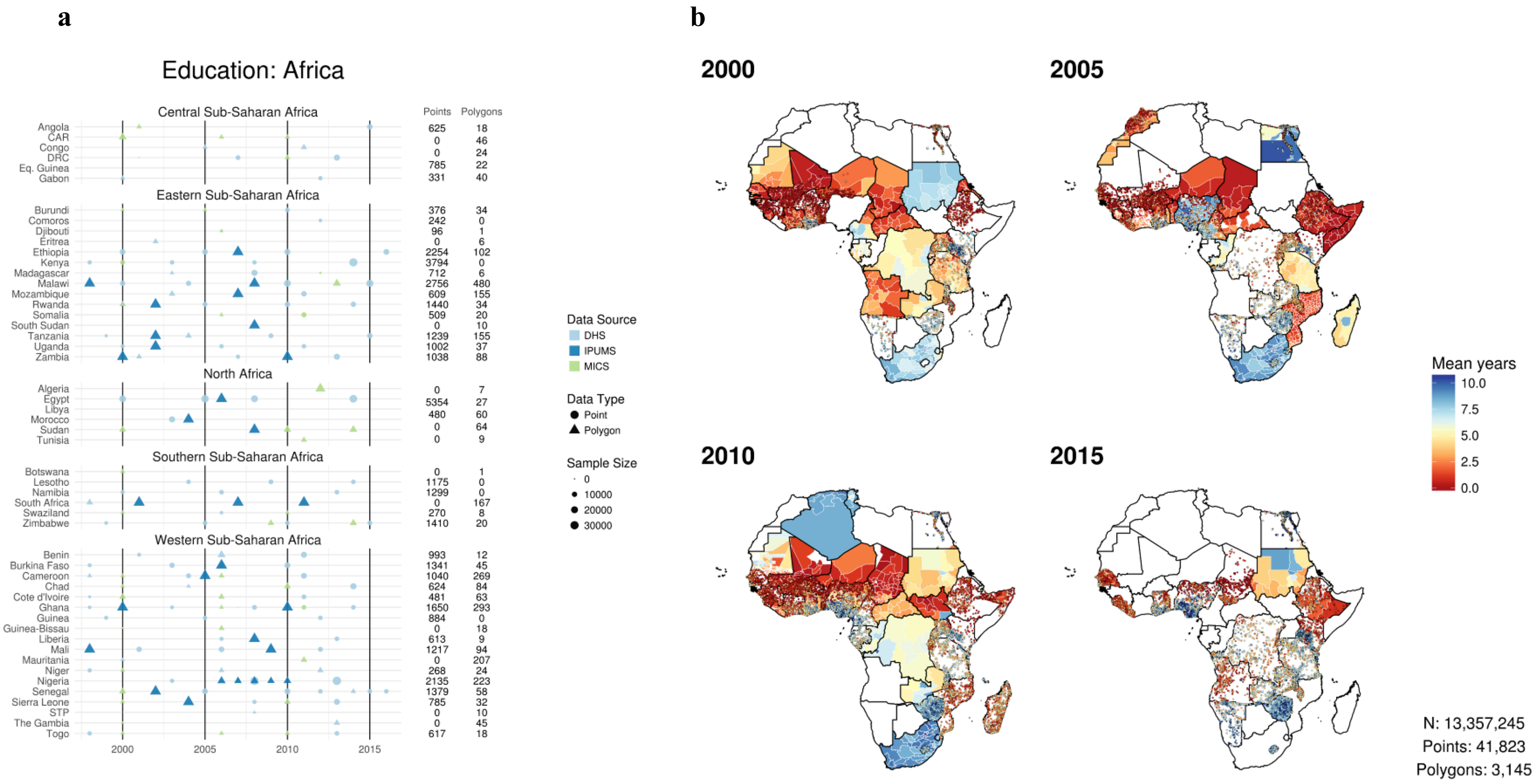

Supplementary Figure 1. Education data availability by type and country for women ages 15-49, 2000–2015. All data are shown by country and year of survey and mapped at their corresponding geopositioned coordinate or area. The total number of points and polygons (areal) for each country are ranked and plotted by data source, type, and sample size (a). The rank column describes a composite metric of data richness, such that each five-year period with any point data adds an integer value of 1 to the country rank, and each five-year period with any polygon data adds a value of 0.01 to the rank. Sample size represents the number of individual microdata records for each survey. Mean years of attainment for the input coordinate or area are mapped (b). This database consists of 39,933 clusters and 3,050 polygons.

doi:10.1038/nature25761

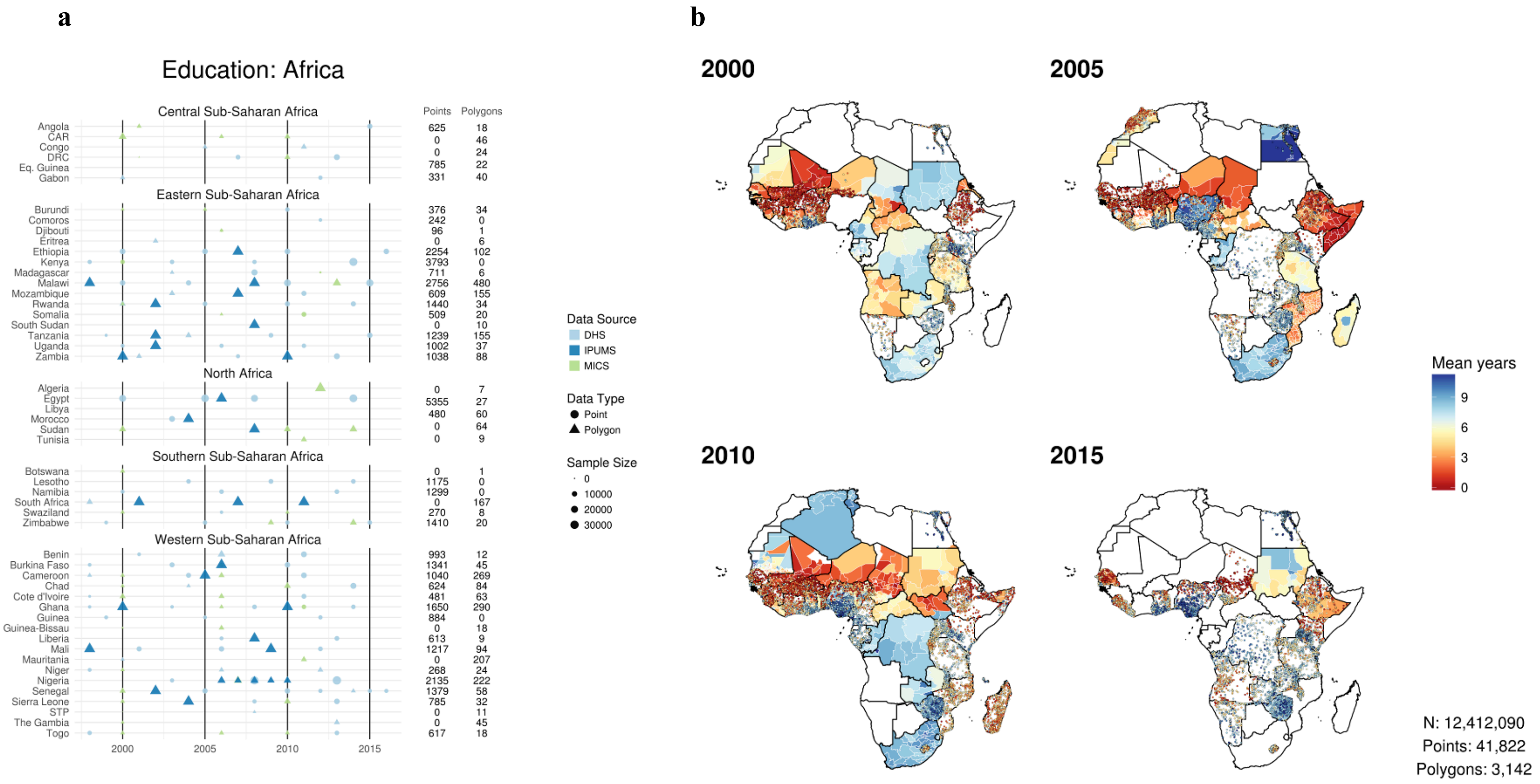

Supplementary Figure 2. Education data availability by type and country for men ages 15-49, 2000–2015. All data are shown by country and year of survey and mapped at their corresponding geopositioned coordinate or area. The total number of points and polygons (areal) for each country are ranked and plotted by data source, type, and sample size (a). The rank column describes a composite metric of data richness, such that each five-year period with any point data adds an integer value of 1 to the country rank, and each five-year period with any polygon data adds a value of 0.01 to the rank. Sample size represents the number of individual microdata records for each survey. Mean years of attainment for the input coordinate or area are mapped (b). This database consists of 39,933 clusters and 3,050 polygons.

doi:10.1038/nature25761

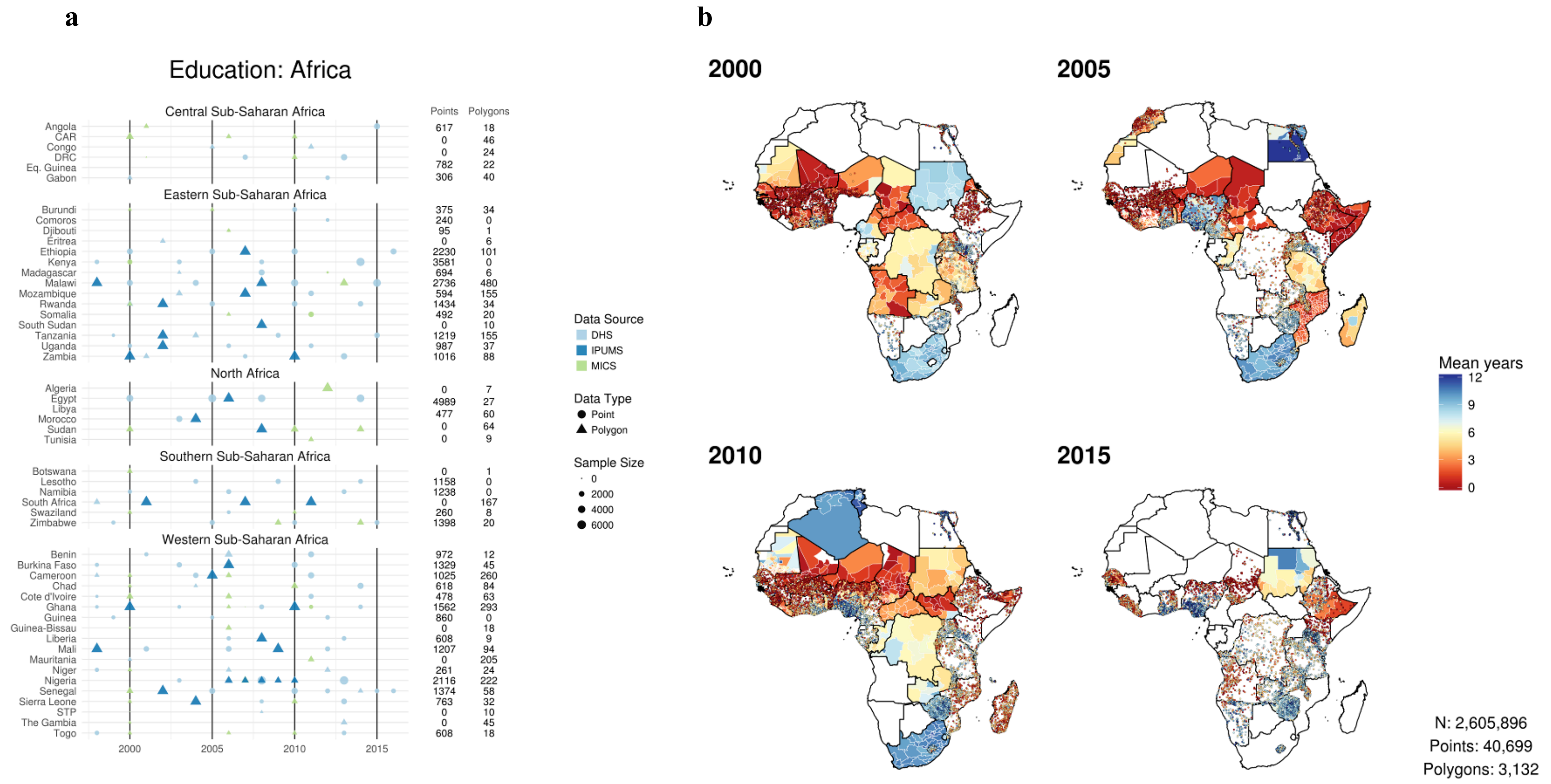

Supplementary Figure 3. **Education data availability by type and country for women ages 20-24, 2000–2015.**
All data are shown by country and year of survey and mapped at their corresponding geopositioned coordinate or area. The total number of points and polygons (areal) for each country are ranked and plotted by data source, type, and sample size (a). The rank column describes a composite metric of data richness, such that each five-year period with any point data adds an integer value of 1 to the country rank, and each five-year period with any polygon data adds a value of 0.01 to the rank. Sample size represents the number of individual microdata records for each survey. Mean years of attainment for the input coordinate or area are mapped (b). This database consists of 39,933 clusters and 3,050 polygons.

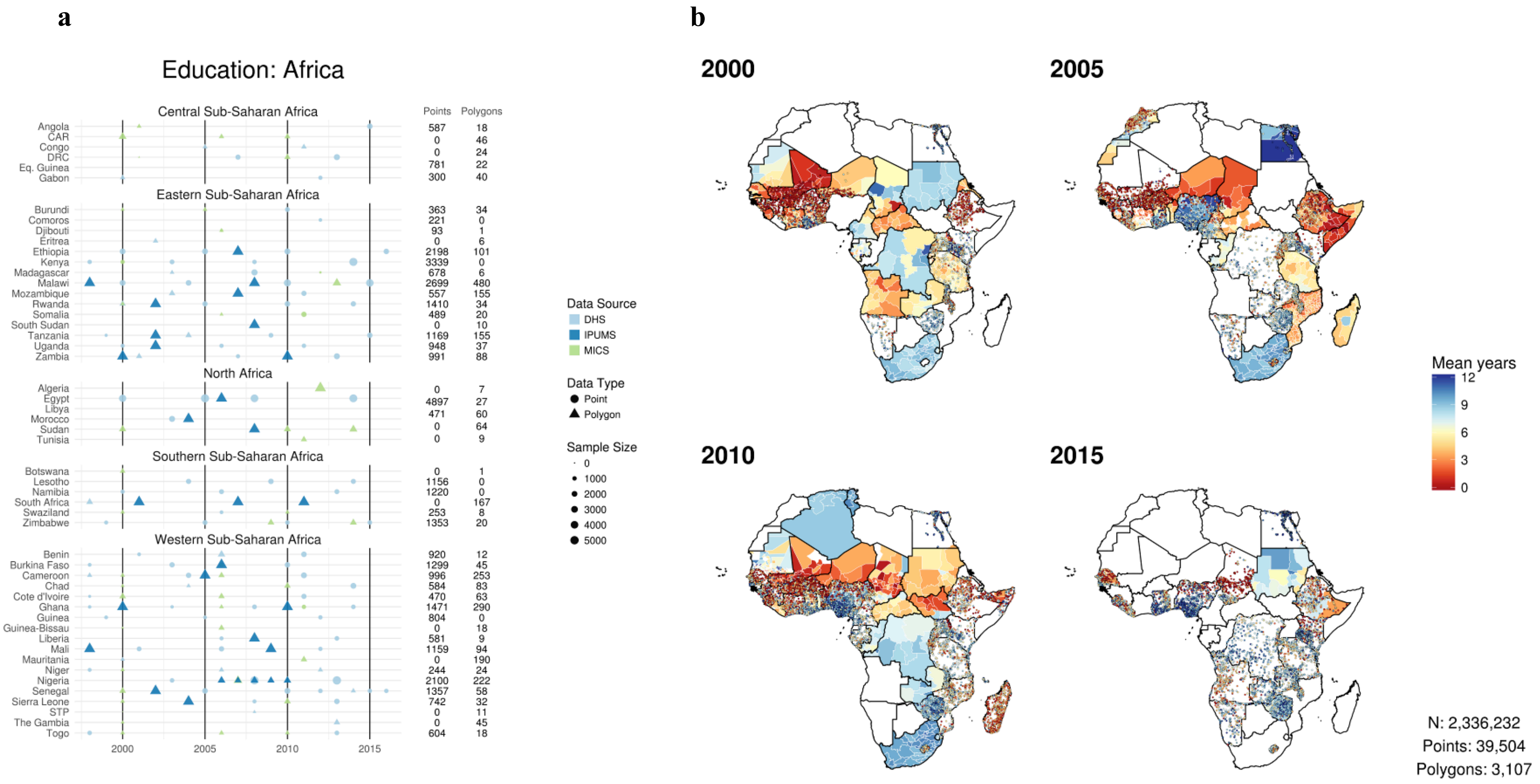

Supplementary Figure 4. Education data availability by type and country for men ages 20-24, 2000–2015. All data are shown by country and year of survey and mapped at their corresponding geopositioned coordinate or area. The total number of points and polygons (areal) for each country are ranked and plotted by data source, type, and sample size (a). The rank column describes a composite metric of data richness, such that each five-year period with any point data adds an integer value of 1 to the country rank, and each five-year period with any polygon data adds a value of 0.01 to the rank. Sample size represents the number of individual microdata records for each survey. Mean years of attainment for the input coordinate or area are mapped (b). This database consists of 39,933 clusters and 3,050 polygons.

4.0 Supplementary covariates

A variety of socioeconomic and environmental variables were used to predict educational attainment. Where available, the finest spatio-temporal resolution of gridded data sets was used.

Supplementary Table 3. Covariates used in mapping

Covariate	Temporal resolution	Source	Reference
Aridity	Annual	Climatic Research Unit Time-Series (CRUTS)	Harris, I., Jones, P. d., Osborn, T. j. & Lister, D. h. Updated high-resolution grids of monthly climatic observations – the CRU TS3.10 dataset. *Int. J. Climatol*. **34**, 623–642 (2014).<br><br>University of East Anglia. Climatic Research Unit TS v. 3.24 dataset. Available at: https://crudata.uea.ac.uk/cru/data/hrg/cru_ts_3.24.01/. (Accessed: 24th July 2017).
Average daily maximum temperature	Annual	CRUTS	Harris, I., Jones, P. d., Osborn, T. j. & Lister, D. h. Updated high-resolution grids of monthly climatic observations – the CRU TS3.10 dataset. *Int. J. Climatol*. **34**, 623–642 (2014).<br><br>University of East Anglia. Climatic Research Unit TS v. 3.24 dataset. Available at: https://crudata.uea.ac.uk/cru/data/hrg/cru_ts_3.24.01/. (Accessed: 24th July 2017).
Average daily mean temperature	Annual	CRUTS	Harris, I., Jones, P. d., Osborn, T. j. & Lister, D. h. Updated high-resolution grids of monthly climatic observations – the CRU TS3.10 dataset. *Int. J. Climatol*. **34**, 623–642 (2014).<br><br>University of East Anglia. Climatic Research Unit TS v. 3.24 dataset. Available at: https://crudata.uea.ac.uk/cru/data/hrg/cru_ts_3.24.01/. (Accessed: 24th July 2017).
Average daily minimum temperature	Annual	CRUTS	Harris, I., Jones, P. d., Osborn, T. j. & Lister, D. h. Updated high-resolution grids of monthly climatic observations – the CRU TS3.10 dataset. *Int. J. Climatol*. **34**, 623–642 (2014).<br><br>University of East Anglia. Climatic Research Unit TS v. 3.24 dataset. Available at:

Covariate	Temporal resolution	Source	Reference
			https://crudata.uea.ac.uk/cru/data/hrg/cru_ts_3.24.01/. (Accessed: 24th July 2017).
Average Land Surface Temperature (LST)	Annual	MODIS	USGS & NASA. Land surface temperature and emissivity 8-day L3 global 1km MOD11A2 dataset. Available at: https://lpdaac.usgs.gov/dataset_discovery/modis/modis_products_table/mod11a2. (Accessed: 24th July 2017)<br><br>Wan, Z. MODIS Land-Surface Temperature Algorithm Theoretical Basis Document (LST ATBD).<br><br>Weiss, D. J. et al. An effective approach for gap-filling continental scale remotely sensed time-series. *Isprs J. Photogramm. Remote Sens*. **98**, 106–118 (2014).
Daytime LST	Annual	MODIS	USGS & NASA. Land surface temperature and emissivity 8-day L3 global 1km MOD11A2 dataset. Available at: https://lpdaac.usgs.gov/dataset_discovery/modis/modis_products_table/mod11a2. (Accessed: 24th July 2017)<br><br>Wan, Z. MODIS Land-Surface Temperature Algorithm Theoretical Basis Document (LST ATBD).<br><br>Weiss, D. J. et al. An effective approach for gap-filling continental scale remotely sensed time-series. *Isprs J. Photogramm. Remote Sens*. **98**, 106–118 (2014).
Distance to rivers	Static	Natural Earth Data (derived)	Natural Earth. Rivers and lake centerlines dataset. Available at: http://www.naturalearthdata.com/downloads/10m-physical-vectors/10m-rivers-lake-centerlines/. (Accessed: 24th July 2017)
Diurnal difference in LST	Annual	MODIS	USGS & NASA. Land surface temperature and emissivity 8-day L3 global 1km MOD11A2 dataset. Available at: https://lpdaac.usgs.gov/dataset_discovery/modis/m

Covariate	Temporal resolution	Source	Reference
			odis_products_table/mod11a2. (Accessed: 24th July 2017)<br><br>Wan, Z. MODIS Land-Surface Temperature Algorithm Theoretical Basis Document (LST ATBD).<br><br>Weiss, D. J. et al. An effective approach for gap-filling continental scale remotely sensed time-series. *Isprs J. Photogramm. Remote Sens*. **98**, 106–118 (2014).
Diurnal temperature range	Annual	CRUTS	Harris, I., Jones, P. d., Osborn, T. j. & Lister, D. h. Updated high-resolution grids of monthly climatic observations – the CRU TS3.10 dataset. *Int. J. Climatol*. **34**, 623–642 (2014).<br><br>University of East Anglia. Climatic Research Unit TS v. 3.24 dataset. Available at: https://crudata.uea.ac.uk/cru/data/hrg/cru_ts_3.24.01/. (Accessed: 24th July 2017).
Enhanced Vegetation Index (EVI)	Annual	MODIS	Huete, A., Justice, C. & van Leeuwen, W. MODIS vegetation index (MOD 13) algorithm theoretical basis document. (1999).<br><br>USGS & NASA. Vegetation indices 16-Day L3 global 500m MOD13A1 dataset. Available at: https://lpdaac.usgs.gov/dataset_discovery/modis/modis_products_table/mod13a1. (Accessed: 25th July 2017)<br><br>Weiss, D. J. et al. An effective approach for gap-filling continental scale remotely sensed time-series. *Isprs J. Photogramm. Remote Sens*. **98**, 106–118 (2014).
Fertility	Annual	WorldPop (derived)	Lloyd, C. T., Sorichetta, A. & Tatem, A. J. High resolution global gridded data for use in population studies. *Sci. Data* **4**, sdata20171 (2017).<br><br>World Pop. Get data. Available at: http://www.worldpop.org.uk/data/get_data/. (Accessed: 25th July 2017)

Covariate	Temporal resolution	Source	Reference
Frost day frequency	Annual	CRUTS	Harris, I., Jones, P. d., Osborn, T. j. & Lister, D. h. Updated high-resolution grids of monthly climatic observations – the CRU TS3.10 dataset. *Int. J. Climatol*. **34**, 623–642 (2014).<br><br>University of East Anglia. Climatic Research Unit TS v. 3.24 dataset. Available at: https://crudata.uea.ac.uk/cru/data/hrg/cru_ts_3.24.01/. (Accessed: 24th July 2017)
Irrigation	Static	University of Frankfurt	Goethe-Universität. Generation of a digital global map of irrigation areas. Available at: https://www.uni-frankfurt.de/45218039/Global_Irrigation_Map. (Accessed: 25th July 2017)
Nighttime LST	Annual	MODIS	USGS & NASA. Land surface temperature and emissivity 8-day L3 global 1km MOD11A2 dataset. Available at: https://lpdaac.usgs.gov/dataset_discovery/modis/modis_products_table/mod11a2. (Accessed: 24th July 2017)<br><br>Wan, Z. MODIS Land-Surface Temperature Algorithm Theoretical Basis Document (LST ATBD).<br><br>Weiss, D. J. et al. An effective approach for gap-filling continental scale remotely sensed time-series. *Isprs J. Photogramm. Remote Sens*. **98**, 106–118 (2014).
Nighttime lights	Annual	NOAA DMSP	NOAA. Version 4 DMSP-OLS nighttime lights time series dataset. Available at: https://www.ngdc.noaa.gov/eog/dmsp/downloadV4composites.html. (Accessed: 25th July 2017)
Normalized Difference Vegetation Index (NDVI)	Annual	AVHRR	NASA & NOAA. Advanced Very High Resolution Radiometer (AVHRR) Normalized Difference Vegetation Index (NDVI) dataset. Available at: https://nex.nasa.gov/nex/projects/1349/. (Accessed: 25th July 2017)
Population	Annual	WorldPop	Lloyd, C. T., Sorichetta, A. & Tatem, A. J. High resolution global gridded data for use in population studies. *Sci. Data* **4**, sdata20171 (2017).

Covariate	Temporal resolution	Source	Reference
			World Pop. Get data. Available at: http://www.worldpop.org.uk/data/get_data/. (Accessed: 25th July 2017)
Potential Evapotranspiration (PET)	Annual	CRUTS	Harris, I., Jones, P. d., Osborn, T. j. & Lister, D. h. Updated high-resolution grids of monthly climatic observations – the CRU TS3.10 dataset. *Int. J. Climatol*. **34**, 623–642 (2014).<br><br>University of East Anglia. Climatic Research Unit TS v. 3.24 dataset. Available at: https://crudata.uea.ac.uk/cru/data/hrg/cru_ts_3.24.01/. (Accessed: 24th July 2017).
Precipitation	Annual	CRUTS	Harris, I., Jones, P. d., Osborn, T. j. & Lister, D. h. Updated high-resolution grids of monthly climatic observations – the CRU TS3.10 dataset. *Int. J. Climatol*. **34**, 623–642 (2014).<br><br>University of East Anglia. Climatic Research Unit TS v. 3.24 dataset. Available at: https://crudata.uea.ac.uk/cru/data/hrg/cru_ts_3.24.01/. (Accessed: 24th July 2017).
Tassled cap brightness	Annual	MODIS	USGS & NASA. Nadir BRDF- Adjusted Reflectance Reflectance 16-Day L3 Global 1km dataset. Available at: https://lpdaac.usgs.gov/dataset_discovery/modis/modis_products_table/mcd43b4. (Accessed: 25th July 2017)<br><br>Strahler, A. H. & Muller, J.-P. MODIS BRDF/Albedo product: algorithm theoretical basis document version 5.0. (1999).<br><br>Weiss, D. J. et al. An effective approach for gap-filling continental scale remotely sensed time-series. *Isprs J. Photogramm. Remote Sens*. **98**, 106–118 (2014).
Tassled cap wetness	Annual	MODIS	USGS & NASA. Nadir BRDF- Adjusted Reflectance Reflectance 16-Day L3 Global 1km dataset. Available at: https://lpdaac.usgs.gov/dataset_discovery/modis/modis_products_table/mcd43b4. (Accessed: 25th July 2017)

Covariate	Temporal resolution	Source	Reference
			Strahler, A. H. & Muller, J.-P. MODIS BRDF/Albedo product: algorithm theoretical basis document version 5.0. (1999).
Travel time to nearest settlement >50,000 inhabitants	Static	Malaria Atlas Project, Big Data Institute, Nuffield Department of Medicine, University of Oxford	Weiss, D. J. *et al.* A global map of travel time to cities to assess inequalities in accessibility in 2015. *Nature* **533,** 333-336 (2018).
Urbanicity	Annual	European Commission/ GHS	Pesaresi, M. et al. Operating procedure for the production of the Global Human Settlement Layer from Landsat data of the epochs 1975, 1990, 2000, and 2014. (Publications Office of the European Union, 2016).
Wet day frequency	Annual	CRUTS	Harris, I., Jones, P. d., Osborn, T. j. & Lister, D. h. Updated high-resolution grids of monthly climatic observations – the CRU TS3.10 dataset. *Int. J. Climatol*. **34**, 623–642 (2014).<br><br>University of East Anglia. Climatic Research Unit TS v. 3.24 dataset. Available at: https://crudata.uea.ac.uk/cru/data/hrg/cru_ts_3.24.01/. (Accessed: 24th July 2017).

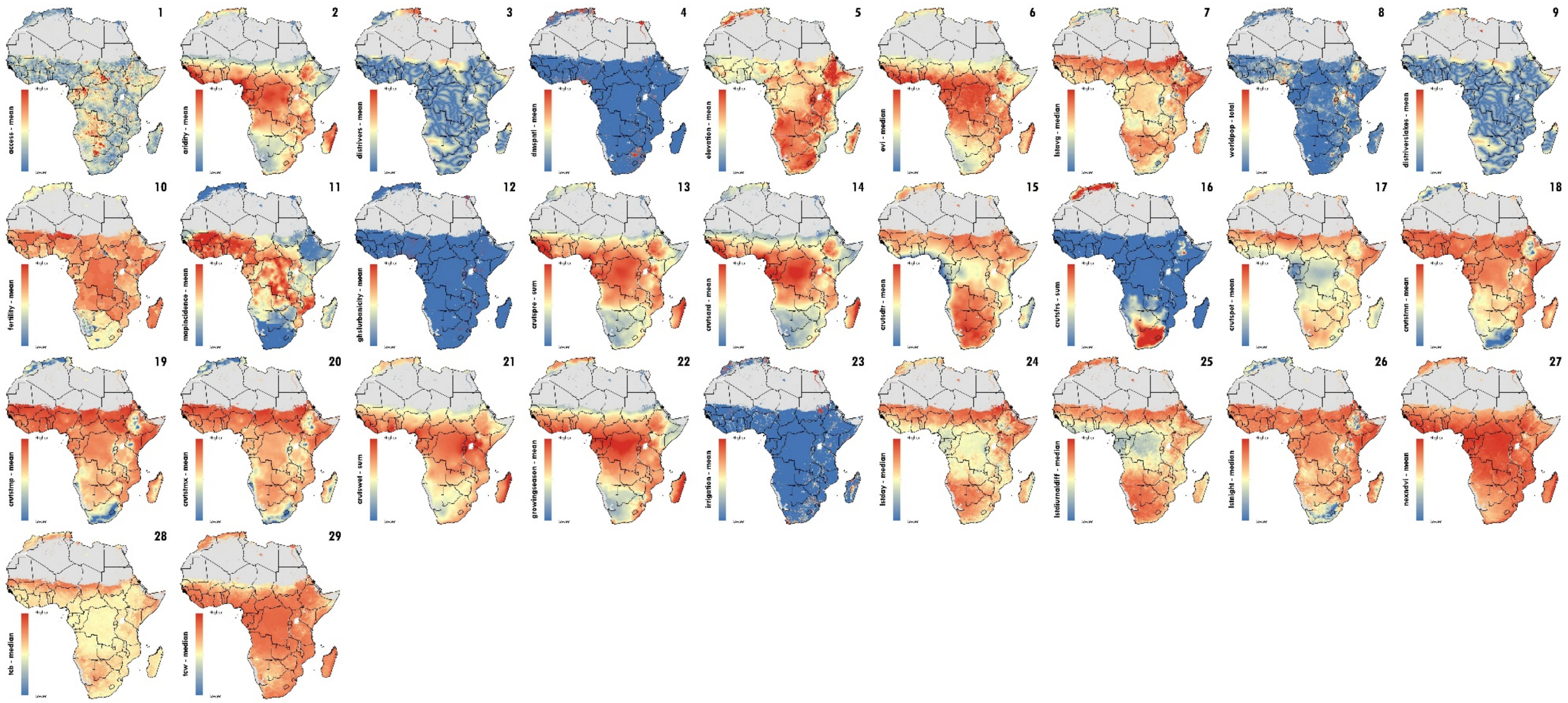

Supplementary Figure 5. Covariates.
Twenty-nine covariate raster layers of possible socioeconomic and environmental correlates of educational attainment in Africa were used as inputs for the stacking modelling process. Time-varying covariates are presented for the year 2015. For the year of production of non-time-varying covariates, please refer to the individual covariate citation in Supplementary Table 3 for additional detail.

5.0 Supplementary methods

5.1 Cluster combination and spatial integration over polygon records

Our individual-level data were collapsed (summarised) into clusters if they could be georeferenced to latitude-longitude pairs. Otherwise, we collapsed our individual-level data to the smallest polygon that could be referenced. We used survey weights and the survey package in R to account for matching our observations to a higher resolution than the representative resolution of the survey.[17,18] The survey package was used to adjust the mean response in the higher resolution polygons using post-stratification weighted averages[19], but we found that the package somewhat regularly produced very large design effects estimates that rendered our polygon sample size adjustments to be nonsensical. Instead, we use the classic Kish's effective sample size calculation where the effective sample size for a polygon is calculated as:

$$n_{eff} = \frac{(\sum w_i)^2}{(\sum w_i{}^2)}$$

where w_i is the survey weight associated with data observation i, and the summations in the effective sample size calculation are both taken over all observation within a polygon.[20]

Data without latitude and longitude, but that could be geolocated to an administrative area, were resampled to generate candidate point locations based on the underlying population of the administrative area. The main concept is to leverage covariate values across the polygon when performing the regression, while simultaneously accounting for a population-driven survey design. The methods used for the resampling are consistent with those used in geospatial modelling of under-5 mortality, published previously.[21]

For each polygon-level observation, 10,000 points were randomly sampled from within the polygon (regardless of the polygon's area) using the WorldPop total population raster[22] to weight the locations of the draws. K-means clustering was performed on the candidate points to generate integration points (1 per 1,000 pixels) used in the modelling. Weights were assigned to each integration point proportionally to the number of candidate points that entered into the k-means cluster, such that the weight of each point represented the number of population-sampled locations contained within the K-means cluster location, divided by the number of sampled points generated (10,000). Each point generated by this process is assigned the mean attainment observed from the survey for that polygon. These sample weights are used in model fit.[23]

5.2 Geostatistical model

5.2.1 Model geographies

A total of five models were run for each indicator based on continuous geographic regions within Africa chosen to align with the regions used in the Global Burden of Disease Study, which determines regions based on both proximity and epidemiological similarity (see tables in Supplementary Fig. 1-3 for listing of regions and countries). Minor changes were made to the

GBD regions to ensure spatial contiguity across Africa (see Extended Data Fig. 2 for an illustration of the modelling regions). All data within the spatial region, and within a one-degree buffer from the boundaries of each region, were included in each model to minimise edge effects.

5.2.2 Ensemble covariate modelling

An ensemble covariate modelling method was implemented in order to both select covariates and capture possible non-linear effects and complex interactions between them.[24] For each region, four sub-models were fit to our dataset, using all of our covariate data as explanatory predictors: generalised additive models, boosted regression trees, and lasso regression. Sample weights are used in sub-models, where applicable, such that cluster locations with latitude and longitude had a sample weight of 1, while cluster locations where the latitude and longitude was generated by the polygon resampling process had a weight based on the K-means clustering process (see 5.1, Cluster combination and spatial integration over polygon records).

Each sub-model is fit using five-fold cross-validation to avoid overfitting. The out-of-sample predictions from across the five holdouts are compiled into a single comprehensive set of predictions from that model. Additionally, the same sub-models were also run using 100% of the data, and a full set of in-sample predictions were created. The five sets of out-of-sample sub-model predictions are fed into the full geostatistical model as the explanatory covariates when performing the model fit. The in-sample predictions from the sub-models are used as the covariates when generating predictions using the fitted full geostatistical model. A recent study has shown that this ensemble approach can improve predictive validity by up to 25% over an individual model.[24]

Predictions from each sub-model are generated based on patterns and relationships between the raw covariates and attainment data, while predictions from the full geostatistical model are generated based on patterns and relationships between the predictions from the ensemble of sub-models and attainment data. To discover the relationships between the sub-model prediction layers (used as covariates in the full geostatistical model) and the attainment data, the only values of the covariates (sub-model prediction layers) "seen" by the model are the values underlying the locations of surveys. As such, it is possible that estimates will be generated in areas where the values of the covariates exceed the minimum and maximum values observed by the model. In these areas, the estimates are generated by extrapolating from the patterns observed within the range of covariates underlying the survey and census data.

5.2.3 Model description

Gaussian data are modeled within a Bayesian hierarchical modeling framework using a spatially and temporally explicit hierarchical generalized linear regression model to fit mean years of education attainment in five regions in Africa as defined in the GBD (Northern, Western,

Southern, Central, and Eastern; see Extended Data Figure 3).[23,25] For each GBD region we approximated the posterior distribution of our Bayesian model:

$$edu_i|\mu_i, \tau, s_i \sim Gaussian(\mu_i, \tau, s_i)$$

$$f_{edu_i|\mu_i,\tau,s_i}(edu_i) = \frac{\sqrt{\tau s_i}}{\sqrt{2\pi}} \exp\left(-\frac{1}{2}\tau s_i (edu_i - \mu_i)^2\right)$$

$$\mu_i = \beta_0 + \boldsymbol{X_i \beta} + \epsilon_{GP_i} + \epsilon_i$$

$$\epsilon_i \sim N(0, \sigma^2_{nug})$$

$$\epsilon_{GP}|\Sigma_{\text{space}}, \Sigma_{time} \sim GP(0, \Sigma_{\text{space}} \otimes \Sigma_{time})$$

$$\Sigma_{\text{space}} = \frac{2^{1-\nu}}{\tau \times \Gamma(\nu)} \times (\kappa \boldsymbol{D})^{\nu} \times \mathrm{K}_{\nu}(\kappa \boldsymbol{D})$$

$$\Sigma_{time\,j,k} = \rho^{|t_k - t_j|}.$$

We model the mean years of attainment at cluster i as Gaussian data given precision τ and a fixed scaling parameter s_i. We use the sample size in each cluster as our scaling parameter. We have suppressed the notation, but the means, edu_i, scaling parameters, s_i, predictions from the three submodels $\boldsymbol{X_i}$, and residual terms ϵ_* are all indexed at a space-time coordinate. The means, edu_i represent an individual's expected educational attainment given that they live at that particular location. Mean attainment was modeled as a linear combination of the three sub-models (GAM, BRT and lasso), $\boldsymbol{X_i}$, a correlated spatiotemporal error term, ϵ_{GP_i}, and an independent nugget effect, ϵ_i. Coefficients, $\boldsymbol{\beta}$, on the sub-models represent their respective predictive weighting in the mean, while the joint error term, ϵ_{GP}, accounts for residual spatiotemporal autocorrelation between individual data points that remains after accounting for the predictive effect of the sub-model covariates and the nugget, ϵ_i, is an independent error term independent. The residuals, ϵ_{GP}, are modeled as a three-dimensional Gaussian process in space-time centered at zero and with a covariance matrix constructed from a Kroenecker product of spatial and temporal covariance kernels. The spatial covariance, Σ_{space}, is modeled using an isotropic and stationary Matérn function[26], and temporal covariance, Σ_{time}, as an annual autoregressive (AR1) function over the 16 years represented in the model. This approach leveraged the data's residual correlation structure to more accurately predict prevalence estimates for locations with no data, while also propagating the dependence in the data through to uncertainty estimates.[27] The posterior distributions were fit using computationally efficient and accurate approximations in R-INLA (integrated nested Laplace approximation) with the

stochastic partial differential equations (SPDE) approximation to the Gaussian process residuals.[28] Pixel-level uncertainty intervals (UIs) were generated from 1,000 draws (*i.e.*, statistically plausible candidate maps)[29] created from the posterior-estimated distributions of modelled parameters.

5.2.4 Priors

The following priors were used for all four of our education models:[23]

- $\beta_0 \sim N(\mu = 0, \sigma^2 = 2^2)$,
- $\boldsymbol{\beta} \sim^{iid} N(\mu = 0, \sigma^2 = 2^2)$,
- $log\left(\frac{1+\rho}{1-\rho}\right) \sim N(\mu = 0, \sigma^2 = 1/0.15)$,
- $log\left(\frac{1}{\sigma_{nug}^2}\right) \sim loggamma(\alpha = 1, \gamma = 2)$.
- $\theta_1 = \log(\tau) \sim N(\mu_{\theta_1}, \sigma_{\theta_1}^2)$
- $\theta_2 = \log(\kappa) \sim N(\mu_2, \sigma_{\theta_2}^2)$

Given that our covariates used in INLA, i.e. the predicted outputs from the ensemble models, should be on the same scale as our predictive target, we believe that the intercept in our model should be close to zero and that the regression coefficients should sum to one. As such, we have chosen the prior for our intercept to be $N(0, \sigma^2 = 3^2)$, and the prior for the fixed effect coefficients to be $N(\frac{1}{\#\text{ ensemble models}}, \sigma^2 = 3^2)$. The prior on the temporal correlation parameter ρ is chosen to be mean zero, showing no prior preference for either positive or negative auto-correlation structure, and with a distribution that is wide enough such that within three standard deviations of the mean the prior includes values of ρ ranging from -0.95 to 0.95. The priors on the random effects variances were chosen to be relatively loose given that we believe our fixed effects covariates should be well correlated with our outcome of interest, which might suggest relatively small random effects values. At the same time, we wanted to avoid using a prior that was so diffuse as to actually put high prior weight on large random effect variances. For stability, we used the uncorrelated multivariate normal priors that INLA automatically determines (based on the finite elements mesh) for the log-transformed spatial hyperparameters κ and τ. The mean and variance parameters for the hyperpriors selected by INLA for the meshes in each region can be found in Supplementary Table 4. In our parameterization we represent α and γ in the *loggamma* distribution as scale and shape, respectively.

Supplementary Table 4. Spatial hyperparameter priors by region

Region	μ_{θ_1}	$\sigma_{\theta_1}^2$	μ_2	$\sigma_{\theta_2}^2$
**Central sub-Saharan Africa**	-0.23082	10	-1.03469	10
**Eastern sub-Saharan Africa**	0.104454	10	-1.36997	10
**Northern Africa**	0.22028	10	-1.48579	10
**Southern sub-Saharan Africa**	-0.17385	10	-1.09166	10
**Western sub-Saharan Africa**	0.181774	10	-1.44729	10

5.2.5 Mesh construction

We constructed the finite elements mesh for the stochastic partial differential equation approximation to the Gaussian process regression using a simplified polygon boundary (in which coastlines and complex boundaries were smoothed) for each of the regions within our model.[23] We set the inner mesh triangle maximum edge length (the mesh size for areas over land) to be 0.2 degrees, and the buffer maximum edge length (the mesh size for areas over the ocean) to be 5.0 degrees. An example of finite elements mesh-constructed for eastern sub-Saharan mesh can be found in Supplementary Figure 12.

5.2.6 Model fitting and estimate generation

Models were fit in INLA with methods consistent with those used in geospatial modelling of under-5 mortality, published previously.[21]

Resampling K-means weights (Supplementary Methods 5.2) were used within the INLA fit by multiplying the corresponding log-likelihood evaluation for the specific observation by the observation's K-means weight. Data points that could be georeferenced to latitude-longitude locations were assigned a weight of 1, ensuring that when the log-likelihood contribution from that observation was evaluated it contributed only to the log-likelihood at the observation's space-time location. For cluster locations generated based on the polygon resampling process, the log-likelihood of those points contributed proportionate to the K-means weights, effectively diffusing the evaluation of the observation across the polygon.

As part of the ensemble modelling process, prediction surfaces from the out-of-sample ensemble sub-models were used as covariates in the spatiotemporal model. Estimates of the fixed effects beta coefficients derived from the contribution of each of the sub-models to INLA's predicted prevalence estimates, in conjunction with parameter estimates of the contribution of location and time (based on estimated parameters described in model description in the Supplementary Methods 5.3.3), were generated and can be found in Supplementary Tables 5-7. To create final estimates, the in-sample prediction surfaces of prevalence from the sub-models (serving as covariates) were used to calculate estimates of prevalence for each pixel in each year.

All estimates were generated by taking 1,000 draws from the posterior distribution, which yielded 1,000 candidate maps used to summarise the pixel- and aggregated-level statistics. For estimates at the pixel level, these draws were used directly to generate estimates and uncertainty. Aggregated estimates, in which estimates at the pixel level were summarised to administrative boundaries, were generated by creating population-weighted averages for each administrative boundary, for each draw.

doi:10.1038/nature25761

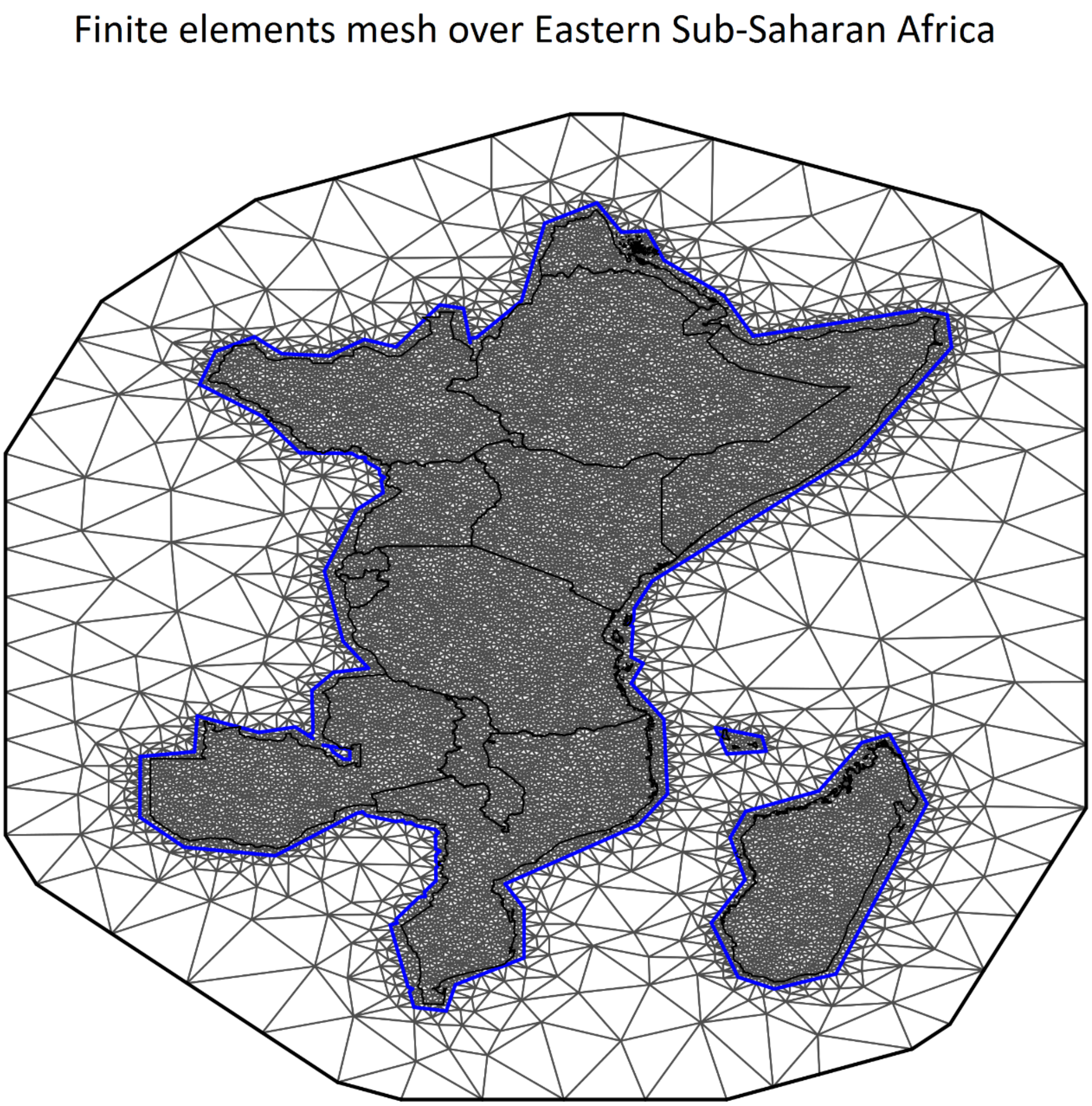

Supplementary Figure 6. Finite elements mesh.
The finite elements mesh used to fit the space-time correlated error for the Eastern sub-Saharan Africa (ESSA) region overlaid on the countries in ESSA. Both the fine-scale mesh over land in the modelling region and the coarser buffer region mesh are shown. The simplified region polygon used to determine the boundary for the modelling region is shown in blue.

5.2.7 Model Results

Fitted parameters and hyperparameters, as well as their 95% uncertainty intervals are shown by indicator and region in Supplementary Tables 5-7. Spatial hyperparameters (τ and κ) and their uncertainties have been transformed into more interpretable nominal variance and range parameters. Nominal variance, approximating the variance at any single point, is calculated as

$nom.var = 4\pi\kappa^2\tau^2$, and nominal range, approximating the distance before spatial correlation decays by 90%, as $range = \sqrt{8}/\kappa$.[20]

doi:10.1038/nature25761

Supplementary Table 5. Education (women, ages 15-49) fitted parameters.
Lower, median, and upper quantiles (0.025%, 0.50%, 0.975%) are displayed for the main parameters by region. The fixed effects covariates corresponding to the predicted ensemble rasters are shown in the first five rows, while fitted values for the spatiotemporal field hyperparameters and the precisions (inverse variance) for our random effects are shown in the bottom five rows.

	Central sub-Saharan Africa quantiles			Eastern sub-Saharan Africa quantiles			Northern Africa quantiles			Southern sub-Saharan Africa quantiles			Western sub-Saharan Africa quantiles		
	**0.025**	**0.500**	**0.975**	**0.025**	**0.500**	**0.975**	**0.025**	**0.500**	**0.975**	**0.025**	**0.500**	**0.975**	**0.025**	**0.500**	**0.975**
**int**	-1.241	-0.223	0.794	-0.076	0.693	1.462	-0.389	0.231	0.851	-0.543	0.065	0.672	0.220	0.382	0.543
**gam**	-0.170	-0.080	0.010	-0.075	-0.047	-0.020	-0.204	-0.125	-0.046	-0.415	-0.364	-0.314	-0.048	-0.003	0.042
**gbm**	0.630	0.721	0.811	0.741	0.763	0.784	0.555	0.612	0.669	0.495	0.540	0.585	0.631	0.663	0.696
**lasso**	0.279	0.359	0.440	0.257	0.285	0.313	0.433	0.513	0.593	0.770	0.824	0.878	0.296	0.340	0.383
**Nominal Range**	10.618	15.009	19.670	6.232	7.574	9.410	3.569	4.820	6.649	2.829	4.071	5.550	2.112	2.458	2.904
**Nominal Variance**	1.473	2.292	3.623	3.460	5.007	7.542	2.012	3.065	4.665	0.988	1.782	2.962	0.454	0.557	0.680
**Ar1 ρ**	0.543	0.761	0.887	0.851	0.890	0.923	0.144	0.595	0.861	0.847	0.920	0.954	0.720	0.797	0.857
**Variance for IID.ID**	0.086	0.104	0.134	0.150	0.161	0.171	0.775	0.873	0.976	0.061	0.076	0.091	0.171	0.184	0.201

doi:10.1038/nature25761

Supplementary Table 6. Education (male, ages 15-49) fitted parameters.
Lower, median, and upper quantiles (0.025%, 0.50%, 0.975%) are displayed for the main parameters by region. The fixed effects covariates corresponding to the predicted ensemble rasters are shown in the first five rows, while fitted values for the spatiotemporal field hyperparameters and the precisions (inverse variance) for our random effects are shown in the bottom five rows.

	Central sub-Saharan Africa quantiles			Eastern sub-Saharan Africa quantiles			Northern Africa quantiles			Southern sub-Saharan Africa quantiles			Western sub-Saharan Africa quantiles		
	**0.025**	**0.500**	**0.975**	**0.025**	**0.500**	**0.975**	**0.025**	**0.500**	**0.975**	**0.025**	**0.500**	**0.975**	**0.025**	**0.500**	**0.975**
**int**	-0.656	-0.082	0.492	-0.010	0.833	1.675	-1.081	-0.375	0.331	-0.204	0.361	0.924	0.284	0.709	1.134
**gam**	-0.140	-0.034	0.072	0.015	0.045	0.075	-0.297	-0.217	-0.137	-0.377	-0.324	-0.271	-0.073	-0.021	0.031
**gbm**	0.512	0.616	0.719	0.474	0.496	0.518	0.326	0.396	0.466	0.521	0.564	0.607	0.477	0.508	0.539
**lasso**	0.331	0.419	0.506	0.430	0.459	0.489	0.740	0.821	0.902	0.706	0.760	0.813	0.462	0.513	0.564
**Nominal Range**	6.025	7.917	11.413	5.535	6.544	7.866	3.805	4.975	6.737	2.645	3.559	5.266	3.629	4.475	5.509
**Nominal Variance**	1.023	1.386	2.049	6.032	8.290	11.724	2.837	4.233	6.514	1.387	2.239	4.229	1.334	1.852	2.593
**Ar1 ρ**	0.618	0.794	0.912	0.843	0.881	0.912	0.294	0.688	0.886	0.909	0.950	0.978	0.703	0.790	0.856
**Variance for IID.ID**	0.081	0.097	0.127	0.162	0.175	0.187	0.330	0.385	0.444	0.077	0.094	0.117	0.235	0.256	0.278

Supplementary Table 7. Education (women, ages 20-24) fitted parameters.
Lower, median, and upper quantiles (0.025%, 0.50%, 0.975%) are displayed for the main parameters by region. The fixed effects covariates corresponding to the predicted ensemble rasters are shown in the first five rows, while fitted values for the spatiotemporal field hyperparameters and the precisions (inverse variance) for our random effects are shown in the bottom five rows.

	Central sub-Saharan Africa quantiles			Eastern sub-Saharan Africa quantiles			Northern Africa quantiles			Southern sub-Saharan Africa quantiles			Western sub-Saharan Africa quantiles		
	**0.025**	**0.500**	**0.975**	**0.025**	**0.500**	**0.975**	**0.025**	**0.500**	**0.975**	**0.025**	**0.500**	**0.975**	**0.025**	**0.500**	**0.975**
**int**	-1.049	-0.227	0.594	0.008	0.878	1.747	-0.737	0.073	0.883	-0.605	0.042	0.689	0.332	0.628	0.924
**gam**	-0.180	-0.084	0.013	0.049	0.078	0.107	-0.209	-0.132	-0.055	-0.323	-0.280	-0.237	-0.105	-0.057	-0.009
**gbm**	0.541	0.636	0.730	0.417	0.437	0.456	0.446	0.514	0.582	0.487	0.532	0.577	0.529	0.563	0.596
**lasso**	0.362	0.448	0.534	0.455	0.485	0.516	0.537	0.618	0.700	0.696	0.748	0.799	0.447	0.494	0.542
**Nominal Range**	6.935	9.339	12.761	6.189	7.203	8.365	3.848	5.283	7.349	3.719	5.210	7.312	2.709	3.216	3.847
**Nominal Variance**	1.827	2.873	4.639	6.222	8.280	11.021	3.433	5.297	8.361	0.930	1.660	3.134	1.048	1.337	1.718
**Ar1 ρ**	0.481	0.704	0.848	0.769	0.819	0.859	0.256	0.708	0.903	0.866	0.923	0.959	0.728	0.805	0.860
**Variance for IID.ID**	0.087	0.120	0.162	0.134	0.147	0.159	0.862	0.989	1.130	0.034	0.042	0.052	0.210	0.230	0.255

doi:10.1038/nature25761

Supplementary Table 8. Education (men, ages 20-24) fitted parameters.
Lower, median, and upper quantiles (0.025%, 0.50%, 0.975%) are displayed for the main parameters by region. The fixed effects covariates corresponding to the predicted ensemble rasters are shown in the first five rows, while fitted values for the spatiotemporal field hyperparameters and the precisions (inverse variance) for our random effects are shown in the bottom five rows.

	Central sub-Saharan Africa quantiles			Eastern sub-Saharan Africa quantiles			Northern Africa quantiles			Southern sub-Saharan Africa quantiles			Western sub-Saharan Africa quantiles		
	**0.025**	**0.500**	**0.975**	**0.025**	**0.500**	**0.975**	**0.025**	**0.500**	**0.975**	**0.025**	**0.500**	**0.975**	**0.025**	**0.500**	**0.975**
**int**	-0.870	-0.134	0.600	0.071	0.855	1.638	-1.495	-0.623	0.250	0.012	0.518	1.024	0.223	0.683	1.142
**gam**	-0.165	-0.045	0.074	-0.040	-0.009	0.023	-0.179	-0.101	-0.023	-0.362	-0.306	-0.250	-0.104	-0.050	0.004
**gbm**	0.346	0.448	0.550	0.468	0.493	0.518	0.210	0.283	0.356	0.437	0.484	0.531	0.436	0.470	0.503
**lasso**	0.500	0.598	0.695	0.484	0.515	0.546	0.740	0.818	0.897	0.762	0.822	0.882	0.528	0.580	0.633
**Nominal Range**	6.436	8.965	12.508	5.284	6.231	7.389	5.288	7.138	10.254	2.500	3.317	4.654	3.570	4.314	5.313
**Nominal Variance**	1.054	1.778	3.063	5.291	7.187	9.898	3.594	5.625	9.161	1.086	1.712	2.918	1.794	2.420	3.339
**Ar1 ρ**	0.677	0.842	0.930	0.805	0.853	0.890	0.249	0.675	0.923	0.917	0.953	0.978	0.678	0.767	0.837
**Variance for IID.ID**	0.103	0.134	0.174	0.120	0.131	0.145	0.301	0.359	0.419	0.055	0.069	0.086	0.233	0.258	0.290

doi:10.1038/nature25761

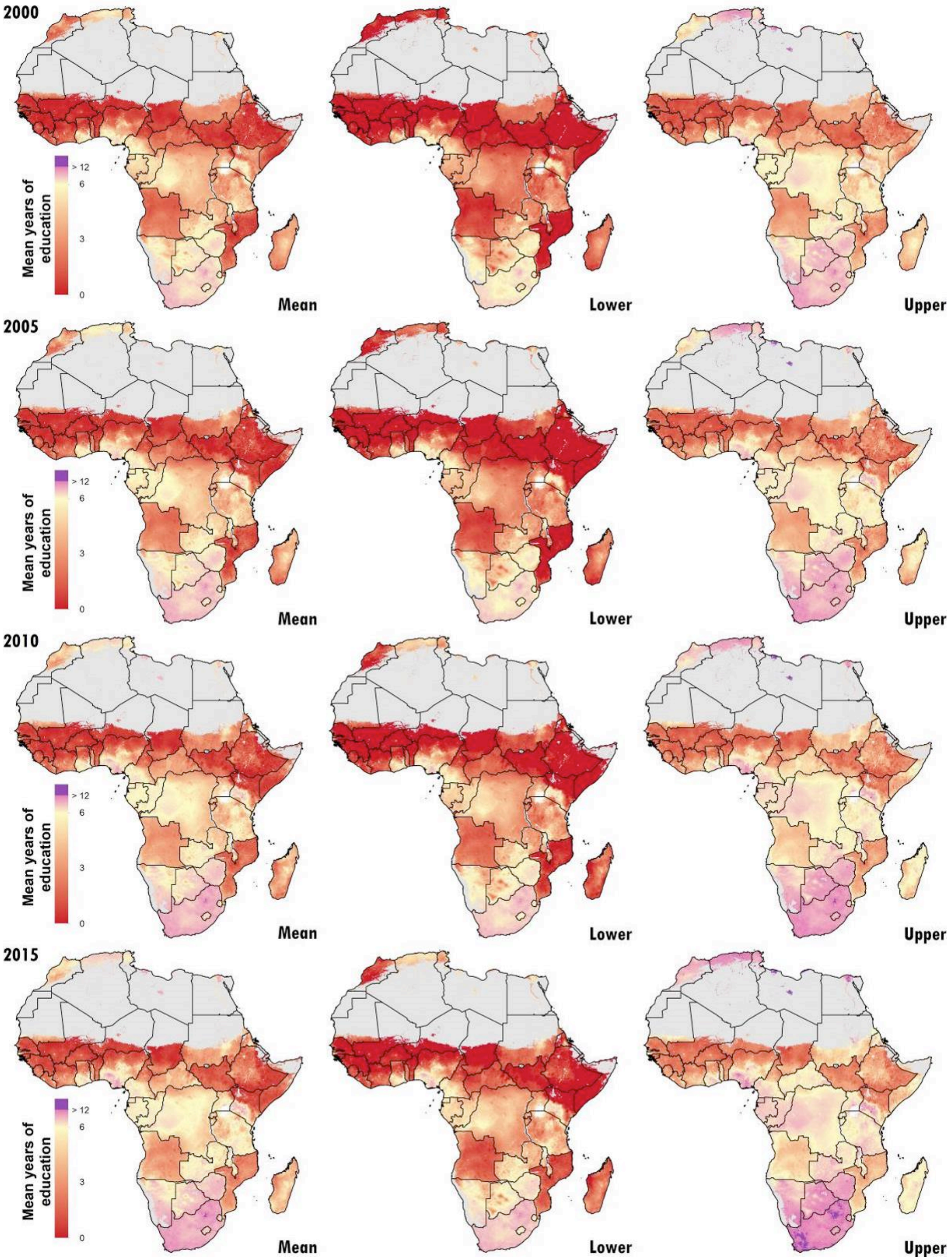

Supplementary Figure 7. Education (women, ages 15-49) posterior means and 95% uncertainty intervals.

doi:10.1038/nature25761

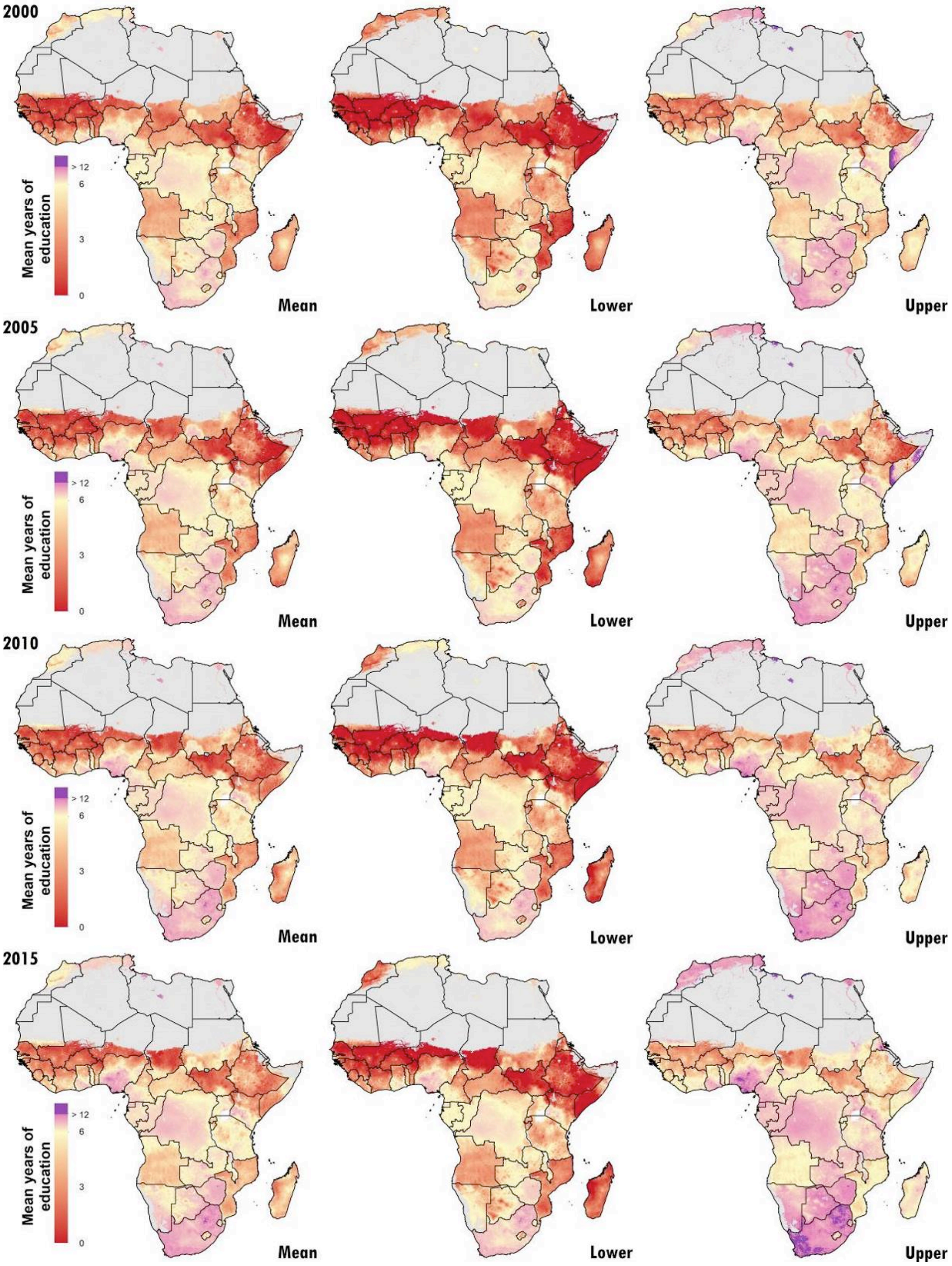

Supplementary Figure 8. Education (men, ages 15-49) posterior means and 95% uncertainty intervals.

doi:10.1038/nature25761

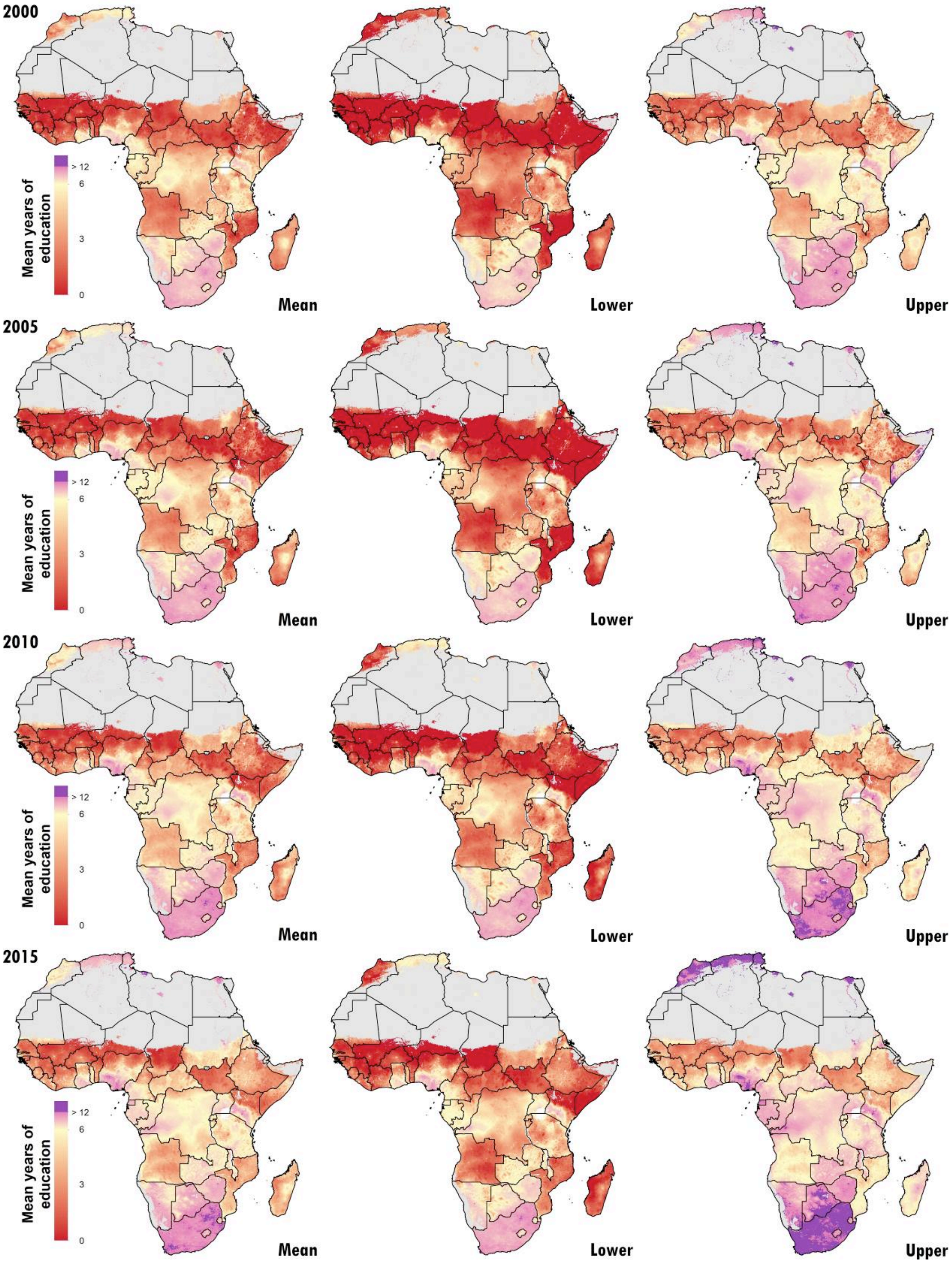

Supplementary Figure 9. Education (women, ages 20-24) posterior means and 95% uncertainty intervals.

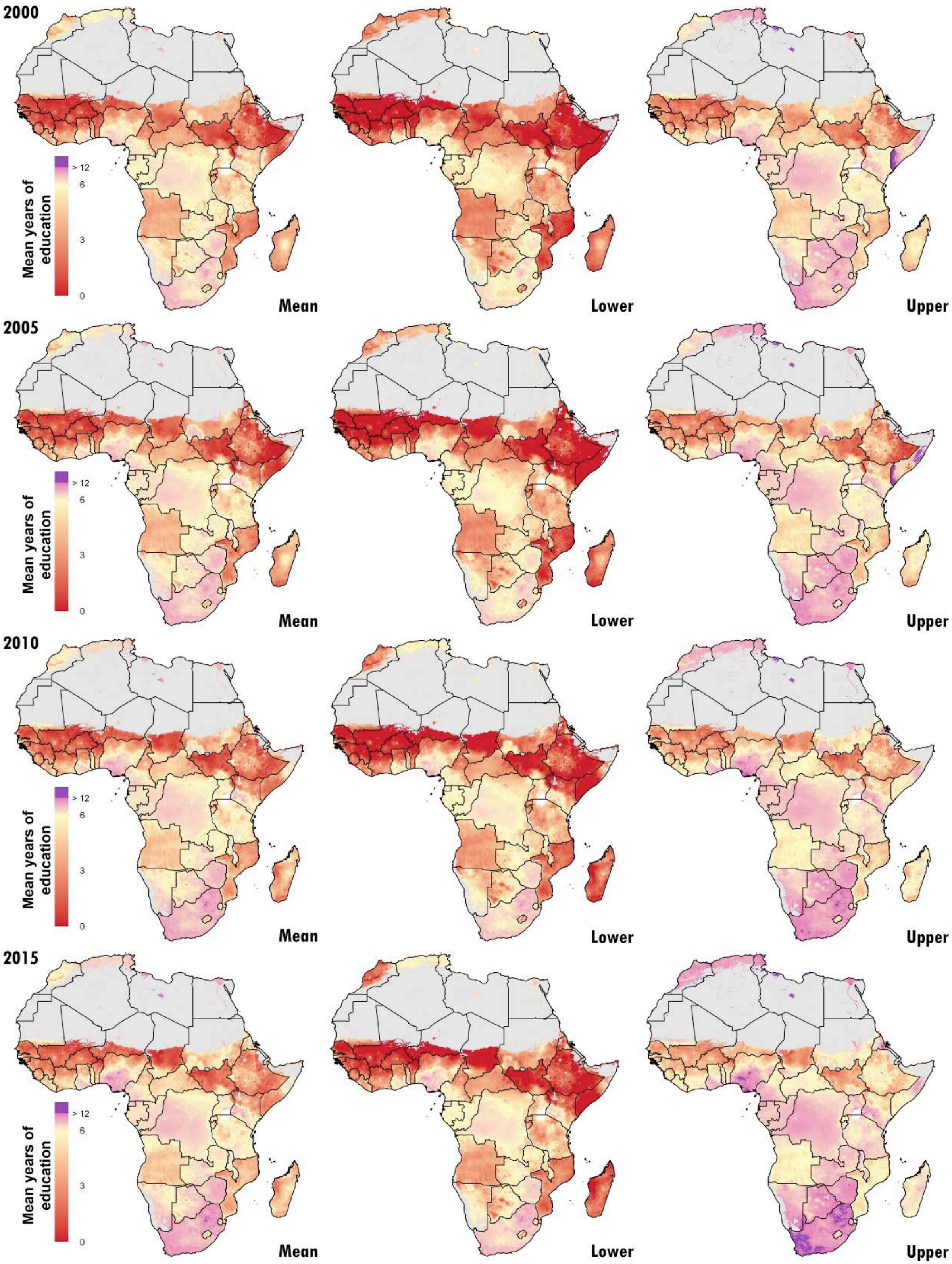

Supplementary Figure 10. Education (men, ages 20-24) posterior means and 95% uncertainty intervals.

5.3 Model validation

5.3.1 In-sample metrics

For each indicator, we generated a suite of diagnostic plots for each region and country estimated, in order to assess the in-sample performance of our model and compare to national-level estimates produced by GBD.

To explore residual error over space and time, absolute error (data minus predicted posterior mean estimates at the corresponding pixels) was produced at five-year intervals (2000, 2005, 2010, and 2015) for each modelled region (see Supplementary Fig. 25-27).

5.3.2 Metrics of predictive validity

In order to assess the predictive validity of our estimates, we validated our models using spatially stratified five-fold out-of-sample cross-validation.[30] To construct each spatial fold, we used a modified bi-tree algorithm to spatially aggregate data points. This algorithm recursively partitions two-dimensional space, alternating between horizontal and vertical splits on the weighted data sample size medians, until the data contained within each spatial partition are of a similar sample size. The depth of recursive partitioning is constrained by the target sample size within a partition and the minimum number of clusters or pseudo-clusters allowed within each spatial partition (in this case, a minimum sample size of 500 was used). These spatial partitions are then allocated to one of five folds for cross-validation. For validation, each geostatistical model was run five times, each time holding out data from one of the folds, generating a set of out-of-sample predictions for the held-out data. For each indicator, a full suite of out-of-sample predictions over the entire dataset was generated by combing the out-of-sample predictions from the five cross-validation runs.

Using these out-of-sample predictions, we then calculated mean error (ME, or bias), root-mean-squared-error (RMSE, which summarises total variance), and 95% coverage of our predictive intervals (the proportion of observed out-of-sample data that fall within our predicted 95% credible intervals) aggregated up to different administrative levels (levels 0, 1, and 2) as defined by FAO Global Administrative Unit Layers (GAUL).[31] Administrative level 0 borders correspond to national boundaries, administrative level 1 borders generally correspond to regions, provinces, or state-level boundaries within a country, and administrative level 2 borders correspond to the next finer subdivision, often districts, within regions. These metrics are summarised in Supplementary Tables 4-12 for each indicator and are calculated across all regions. Included in the sample tables for comparison are the same metrics calculated on in-sample predictions.

5.3.3 Education (women, ages 15-49) validation metrics

Supplementary Table 9. Predictive metrics for education (women, ages 15-49) aggregated to admin 0.
The out-of-sample (OOS) column indicates whether the metric was calculated using in-sample or out-of-sample predictions.

Year	OOS	Median SS	Mean err.	RMSE	Corr.	95% Cov.
**2000**	FALSE	12964	-0.020	0.311	0.996	0.976
**2005**	FALSE	10262	0.410	0.618	0.989	0.951
**2010**	FALSE	13559	-0.073	0.363	0.998	0.976
**2015**	FALSE	15795	0.145	0.367	0.986	0.877

Supplementary Table 10. Predictive metrics for education (women, ages 15-49) aggregated to admin 1.

Year	OOS	Median SS	Mean err.	RMSE	Corr.	95% Cov.
**2000**	FALSE	1249	-0.006	0.406	0.992	0.978
**2005**	FALSE	729	0.409	0.786	0.979	0.949
**2010**	FALSE	799	-0.073	0.446	0.995	0.976
**2015**	FALSE	935	0.140	0.430	0.987	0.879

Supplementary Table 11. Predictive metrics for education (women, ages 15-49) aggregated to admin 2.

Year	OOS	Median SS	Mean err.	RMSE	Corr.	95% Cov.
**2000**	FALSE	141	-0.013	0.534	0.984	0.979
**2005**	FALSE	76	0.409	1.051	0.956	0.957
**2010**	FALSE	95	-0.073	0.595	0.988	0.975
**2015**	FALSE	71	0.136	0.812	0.960	0.877

Supplementary Table 12. Predictive metrics for education (women, ages 15-49) aggregated to holdout units.

Year	OOS	Median SS	Mean err.	RMSE	Corr.	95% Cov.
**2000**	FALSE	31	-0.207	1.191	0.900	0.987
**2005**	FALSE	30	0.409	1.223	0.938	0.960
**2010**	FALSE	30	-0.072	0.840	0.973	0.974
**2015**	FALSE	27	0.140	1.401	0.889	0.876
**2000**	TRUE	31	-0.136	1.215	0.887	0.978
**2005**	TRUE	30	0.258	1.111	0.945	0.970
**2010**	TRUE	30	0.101	0.904	0.962	0.965
**2015**	TRUE	27	0.101	1.493	0.873	0.857

doi:10.1038/nature25761

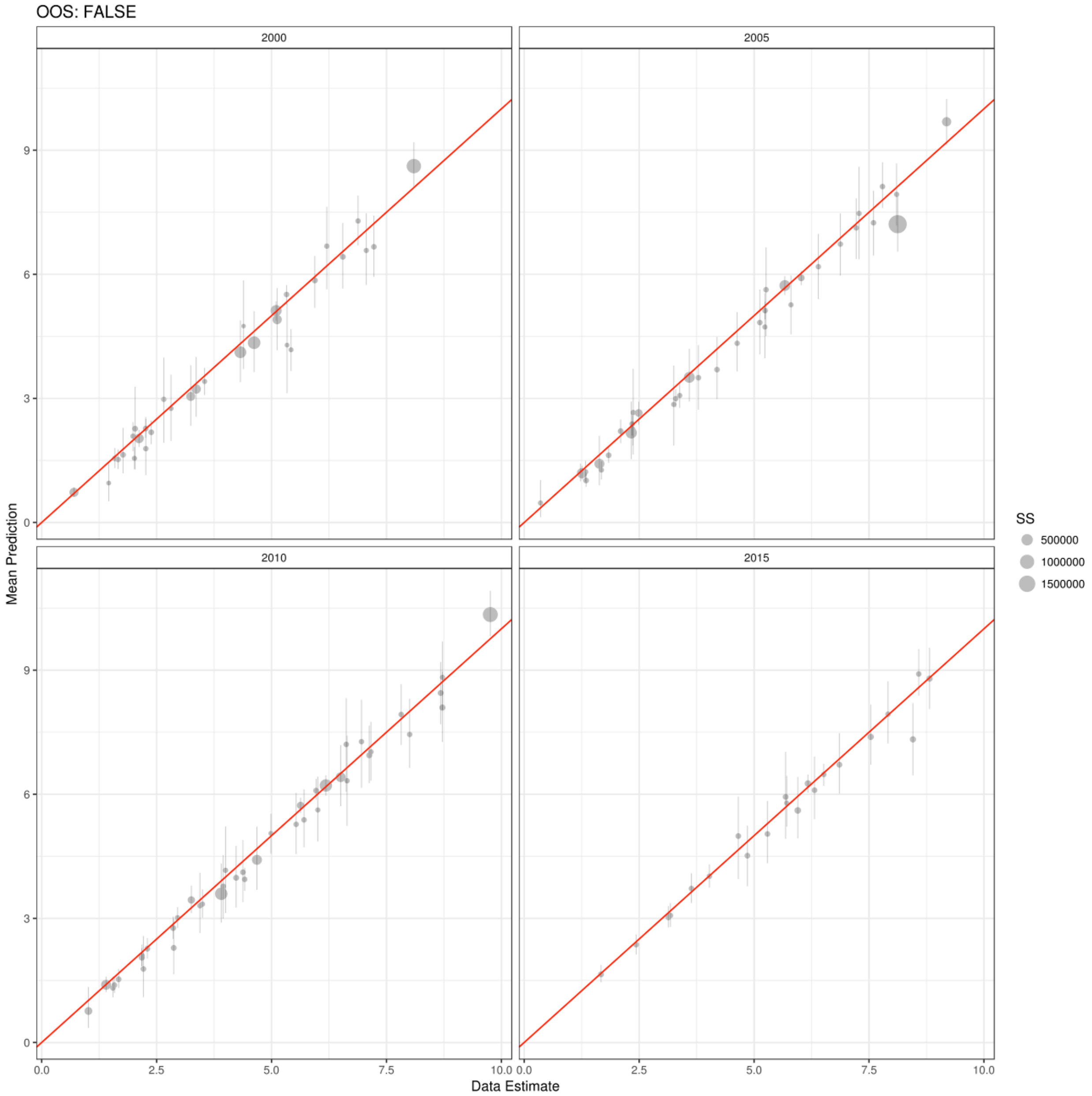

Supplementary Figure 11. Education (women, ages 15-49) admin 0 aggregation. Comparison of in-sample education predictions aggregated to admin 0 with 95% uncertainty intervals plotted against admin 0 aggregated data observations.

doi:10.1038/nature25761

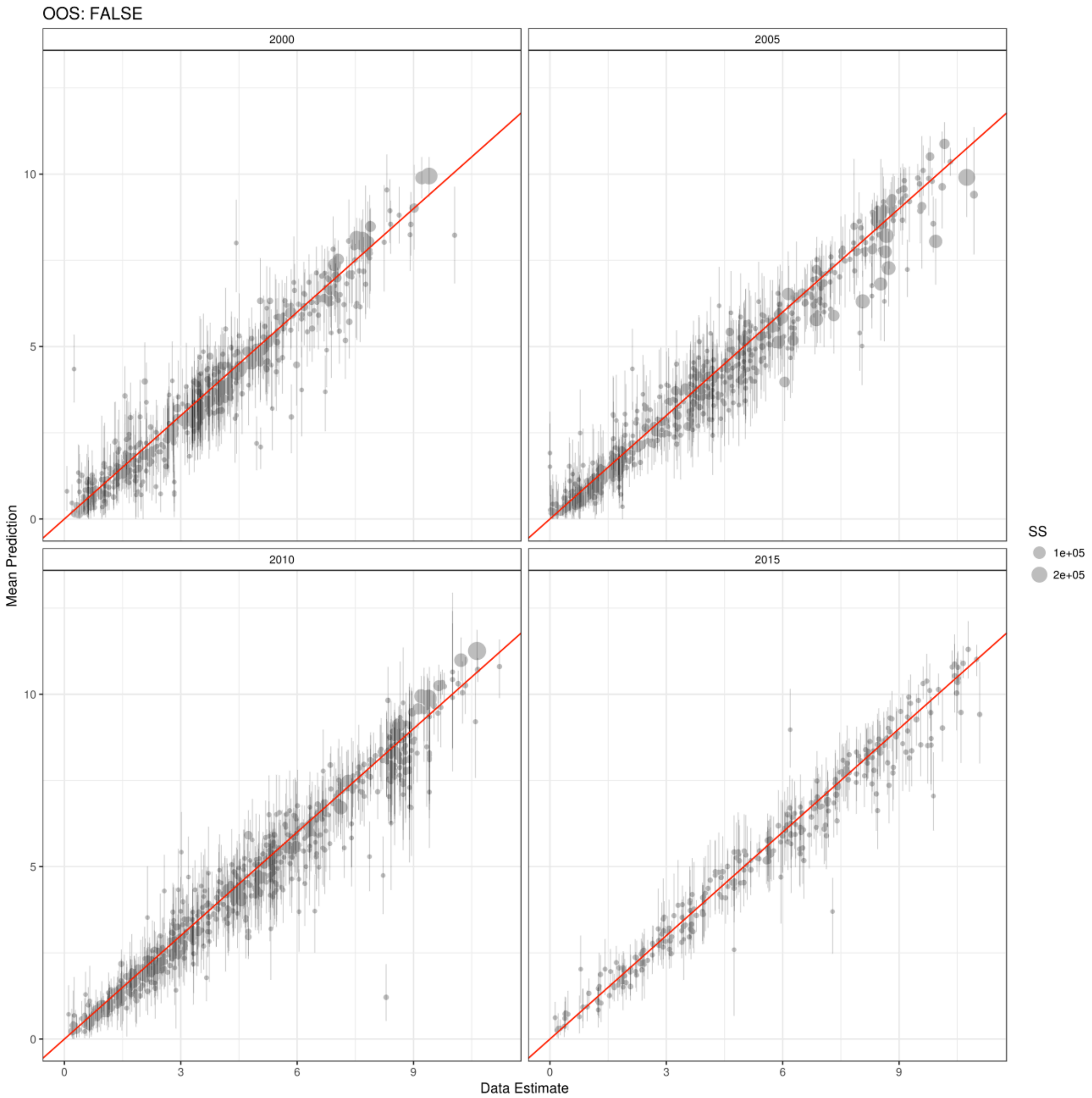

Supplementary Figure 12. Education (women, ages 15-49) admin 1 aggregation. Comparison of in-sample education predictions aggregated to admin 1 with 95% uncertainty intervals plotted against admin 1 aggregated data observations.

doi:10.1038/nature25761

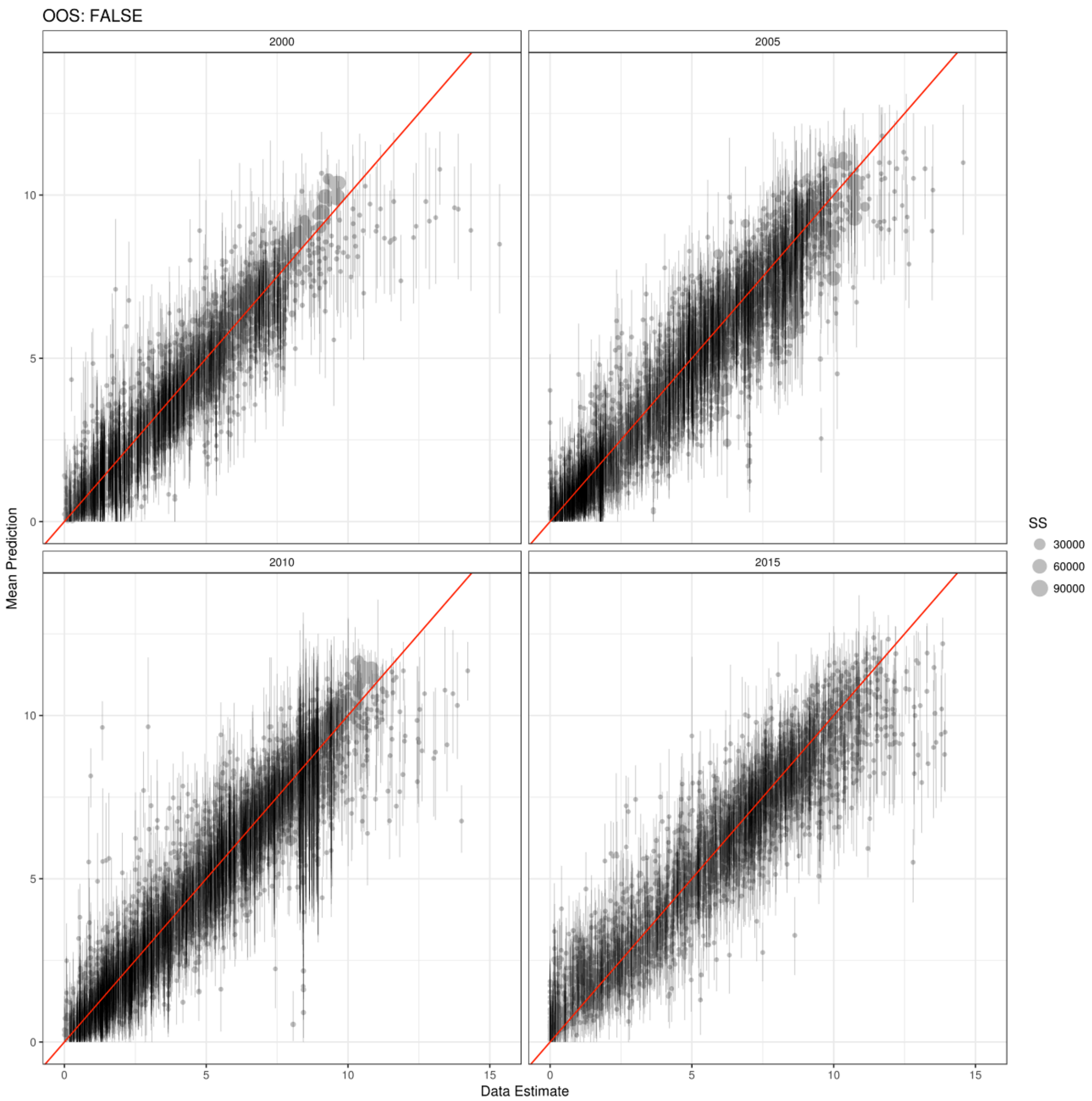

Supplementary Figure 13. Education (women, ages 15-49) admin 2 aggregation. Comparison of in-sample education predictions aggregated to admin 2 with 95% uncertainty intervals plotted against admin 2 aggregated data observations.

doi:10.1038/nature25761

5.3.4 Education (women, ages 20-24) validation metrics

Supplementary Table 13. Predictive metrics for education (women, ages 20-24) aggregated to admin 0.

The out-of-sample (OOS) column indicates whether the metric was calculated using in-sample or out-of-sample predictions.

Year	OOS	Median SS	Mean err.	RMSE	Corr.	95% Cov.
**2000**	FALSE	2533	0.032	0.254	0.998	0.969
**2005**	FALSE	2157	0.479	0.635	0.996	0.970
**2010**	FALSE	2751	0.166	0.229	0.999	0.970
**2015**	FALSE	3208	-0.100	0.388	0.981	0.867

Supplementary Table 14. Predictive metrics for education (women, ages 20-24) aggregated to admin 1.

Year	OOS	Median SS	Mean err.	RMSE	Corr.	95% Cov.
**2000**	FALSE	259	0.047	0.397	0.994	0.971
**2005**	FALSE	147	0.479	0.743	0.991	0.974
**2010**	FALSE	166	0.166	0.341	0.996	0.966
**2015**	FALSE	130	-0.103	0.520	0.981	0.867

Supplementary Table 15. Predictive metrics for education (women, ages 20-24) aggregated to admin 2.

Year	OOS	Median SS	Mean err.	RMSE	Corr.	95% Cov.
**2000**	FALSE	30	0.039	0.542	0.985	0.971
**2005**	FALSE	16	0.479	0.966	0.978	0.973
**2010**	FALSE	19	0.165	0.559	0.988	0.967
**2015**	FALSE	14	-0.106	0.903	0.947	0.867

Supplementary Table 16. Predictive metrics for education (women, ages 20-24) aggregated to holdout units.

Year	OOS	Median SS	Mean err.	RMSE	Corr.	95% Cov.
**2000**	FALSE	7	-0.165	1.169	0.908	0.986
**2005**	FALSE	6	0.480	1.140	0.966	0.962
**2010**	FALSE	10	0.166	0.781	0.975	0.969
**2015**	FALSE	4	-0.104	1.094	0.919	0.865
**2000**	TRUE	7	-0.062	1.232	0.892	0.981
**2005**	TRUE	6	0.425	1.137	0.966	0.963
**2010**	TRUE	10	0.092	1.023	0.953	0.944
**2015**	TRUE	4	0.224	1.293	0.892	0.835

doi:10.1038/nature25761

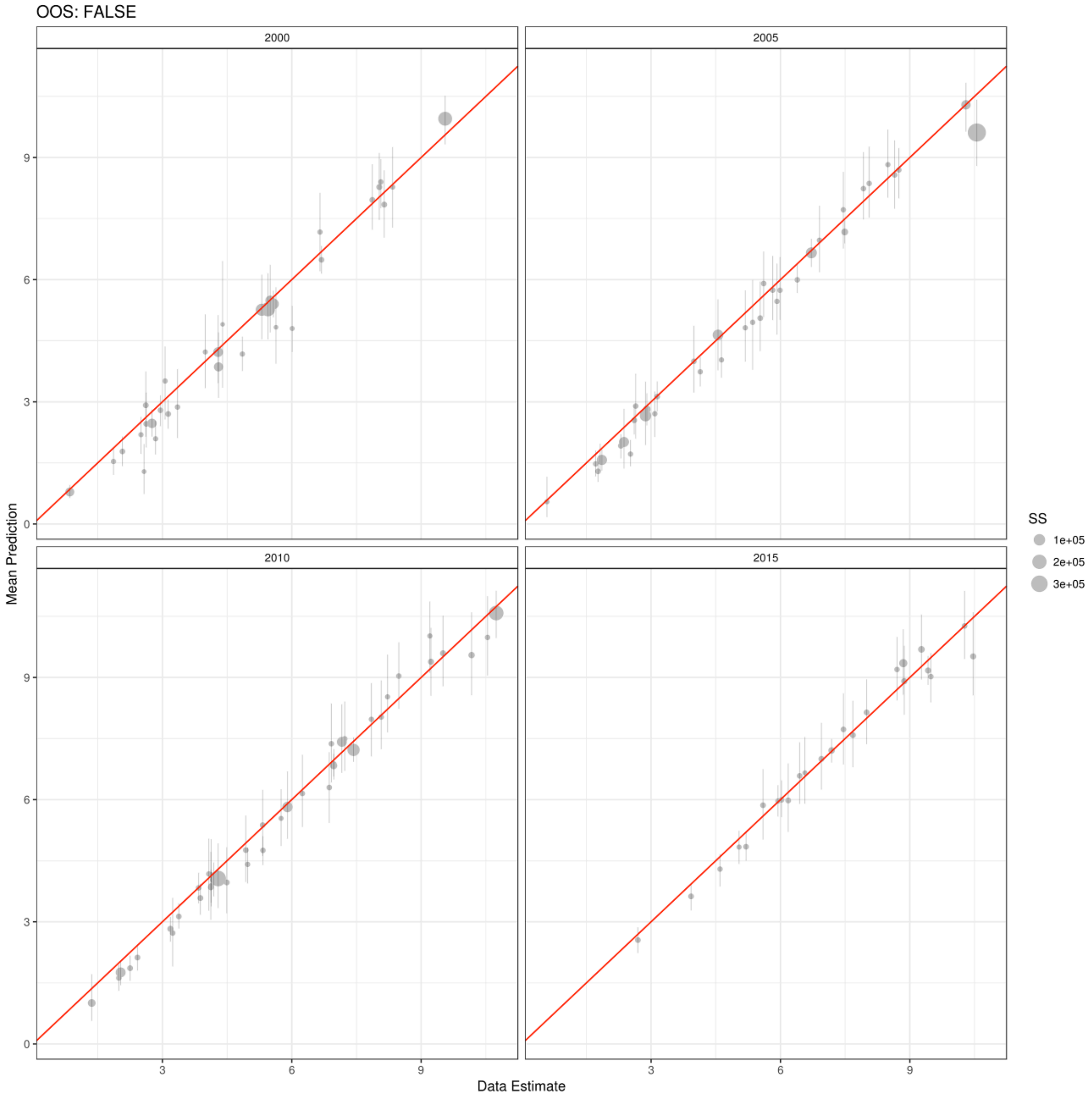

Supplementary Figure 14. Education (women, ages 20-24) admin 0 aggregation. Comparison of in-sample education predictions aggregated to admin 0 with 95% uncertainty intervals plotted against admin 0 aggregated data observations.

doi:10.1038/nature25761

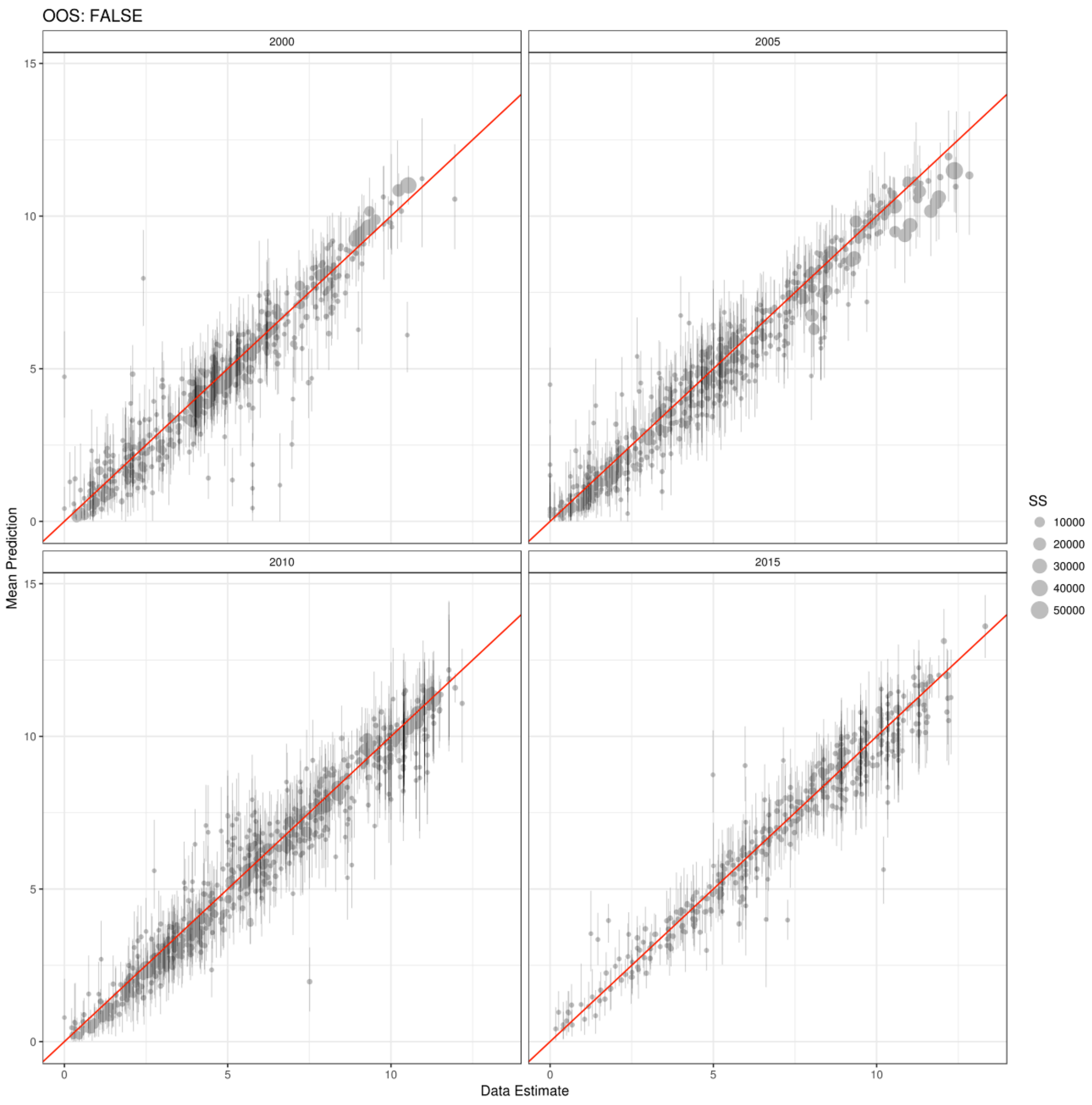

Supplementary Figure 15. Education (women, ages 20-24) admin 1 aggregation. Comparison of in-sample education predictions aggregated to admin 1 with 95% uncertainty intervals plotted against admin 1 aggregated data observations.

doi:10.1038/nature25761

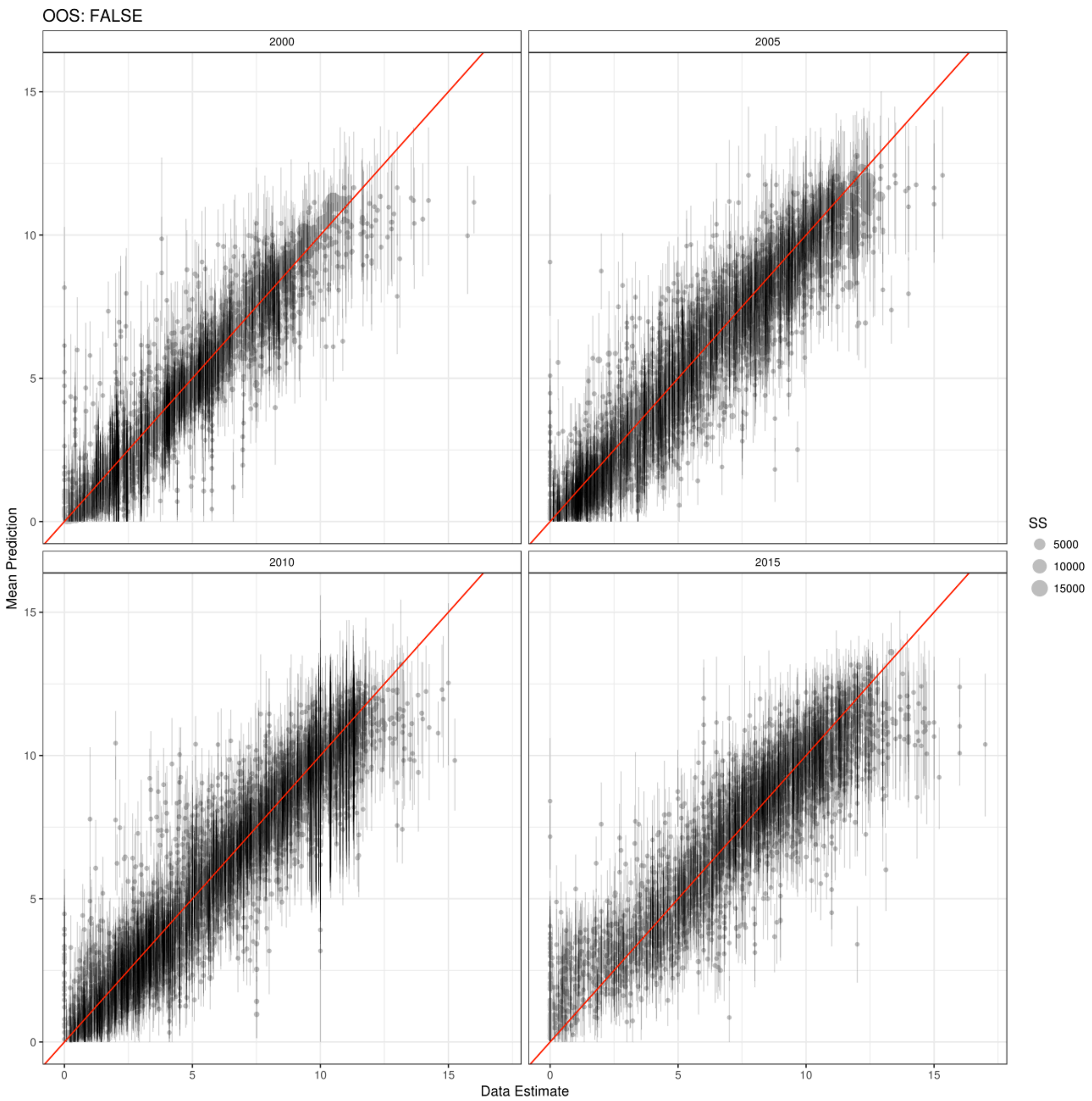

Supplementary Figure 16. Education (women, ages 20-24) admin 2 aggregation. Comparison of in-sample education predictions aggregated to admin 2 with 95% uncertainty intervals plotted against admin 2 aggregated data observations**.**

5.3.5 *Education (men, ages 15-49) validation metrics*

Supplementary Table 17. Predictive metrics for education (men, ages 15-49) aggregated to admin 0.
The out of sample (OOS) column indicates whether the metric was calculated using in-sample or out-of-sample predictions.

Year	OOS	Median SS	Mean err.	RMSE	Corr.	95% Cov.
2000	FALSE	11971	-0.047	0.226	0.997	0.973
2005	FALSE	9297	0.010	0.227	0.997	0.980
2010	FALSE	12121	-0.026	0.268	0.997	0.970
2015	FALSE	12969	-0.073	0.376	0.985	0.860

Supplementary Table 18. Predictive metrics for education (men, ages 15-49) aggregated to admin 1

Year	OOS	Median SS	Mean err.	RMSE	Corr.	95% Cov.
2000	FALSE	1302	-0.026	0.377	0.991	0.971
2005	FALSE	693	0.010	0.384	0.993	0.978
2010	FALSE	828	-0.025	0.368	0.994	0.971
2015	FALSE	920	-0.077	0.479	0.982	0.857

Supplementary Table 19. Predictive metrics for education (men, ages 15-49) aggregated to admin 2

Year	OOS	Median SS	Mean err.	RMSE	Corr.	95% Cov.
2000	FALSE	143	-0.034	0.516	0.980	0.973
2005	FALSE	77	0.009	0.602	0.983	0.980
2010	FALSE	97	-0.026	0.571	0.983	0.970
2015	FALSE	67	-0.080	0.926	0.934	0.853

Supplementary Table 20. Predictive metrics for education (men, ages 15-49) aggregated to holdout units

Year	OOS	Median SS	Mean err.	RMSE	Corr.	95% Cov.
2000	FALSE	30	-0.250	1.225	0.849	0.986
2005	FALSE	30	0.011	0.827	0.966	0.980
2010	FALSE	31	-0.025	0.883	0.958	0.972
2015	FALSE	27	-0.073	1.442	0.849	0.859
2000	TRUE	30	-0.289	1.265	0.834	0.977
2005	TRUE	30	0.031	0.939	0.953	0.966
2010	TRUE	31	-0.072	1.001	0.939	0.947
2015	TRUE	27	-0.019	1.519	0.829	0.846

doi:10.1038/nature25761

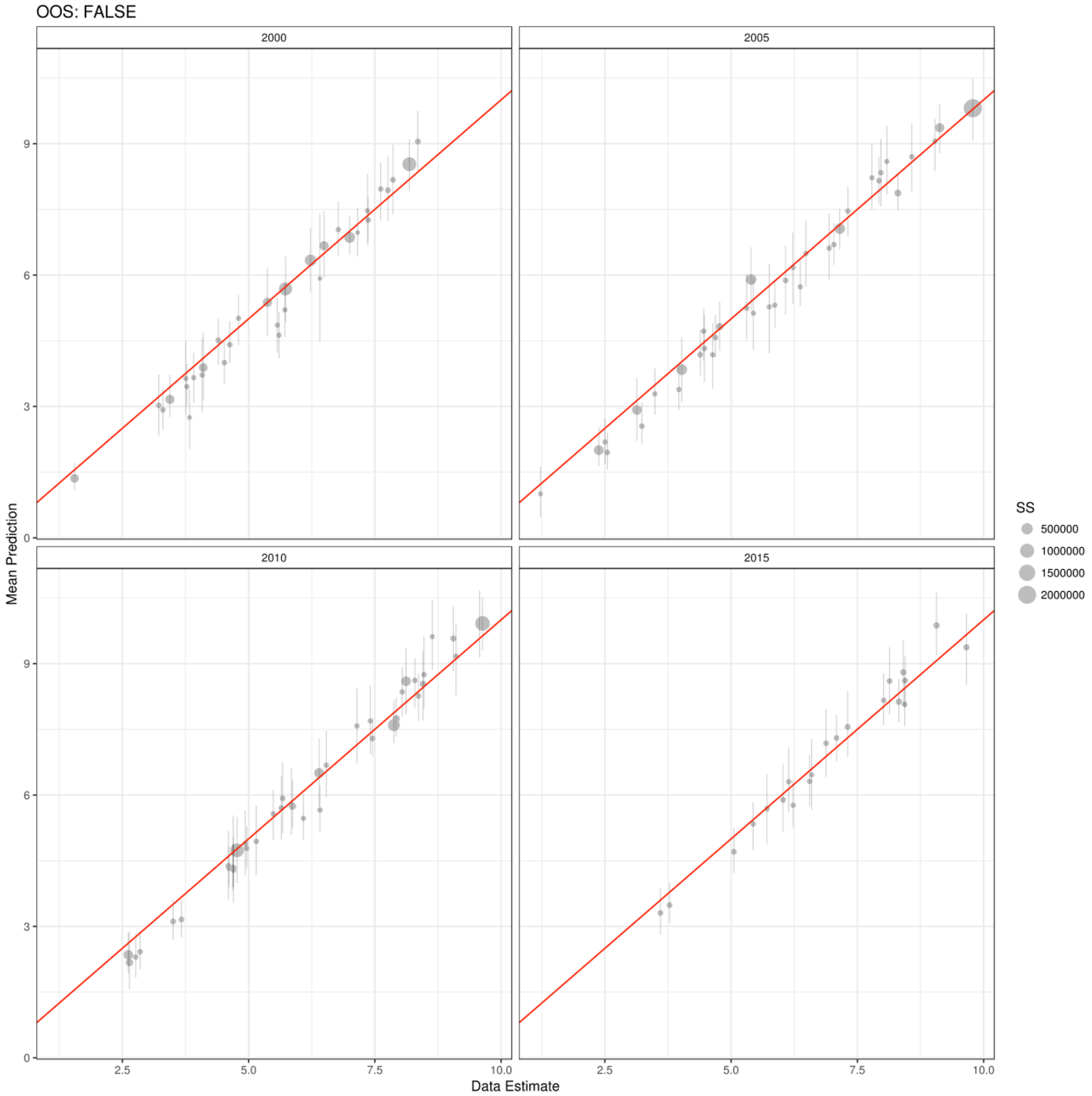

Supplementary Figure 17. **Education (men, ages 15-49) admin 0 aggregation.** Comparison of in-sample education predictions aggregated to admin 0 with 95% uncertainty intervals plotted against admin 0 aggregated data observations.

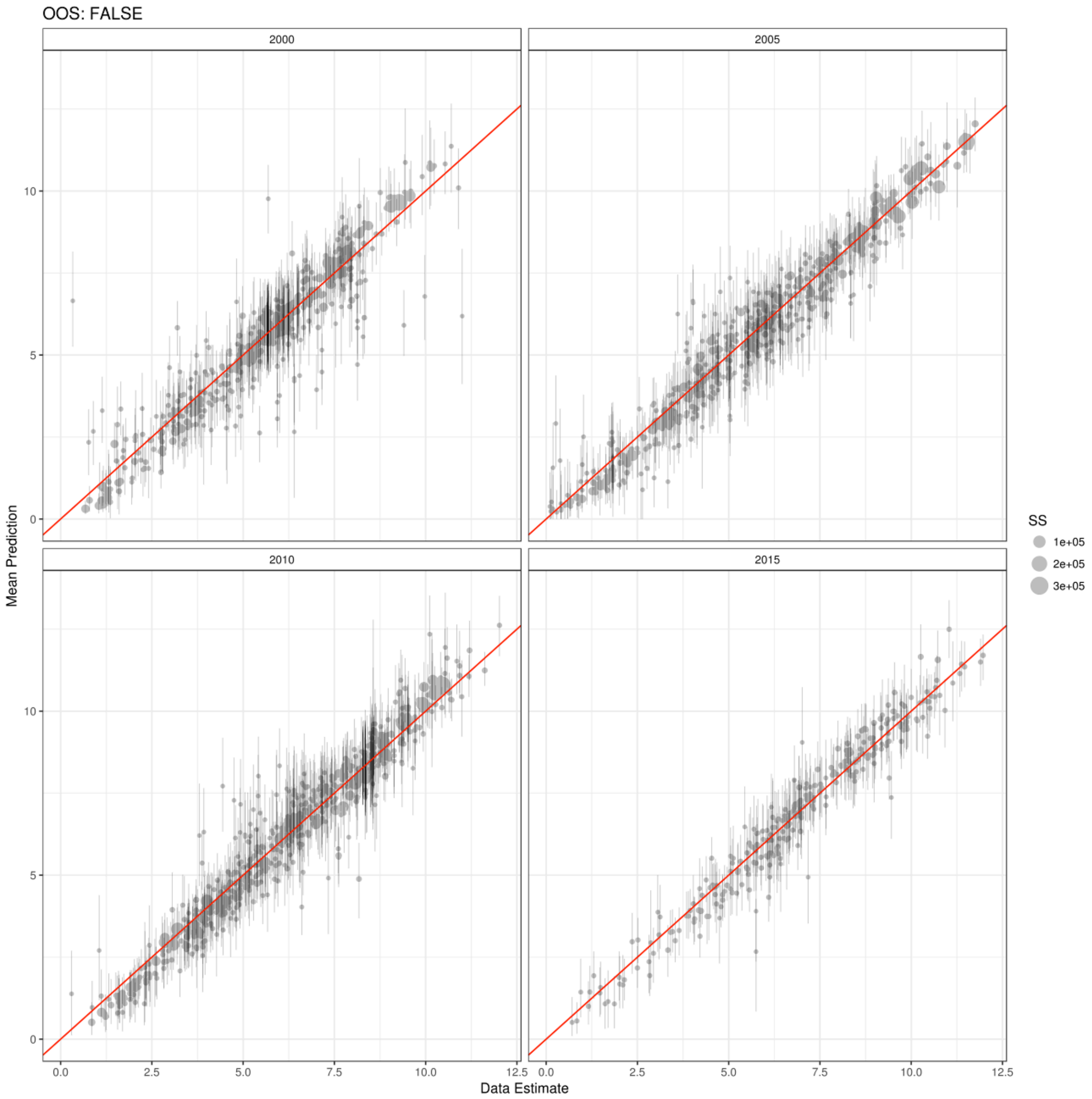

Supplementary Figure 18. Education (men, ages 15-49) admin 1 aggregation. Comparison of in-sample education predictions aggregated to admin 1 with 95% uncertainty intervals plotted against admin 1 aggregated data observations.

doi:10.1038/nature25761

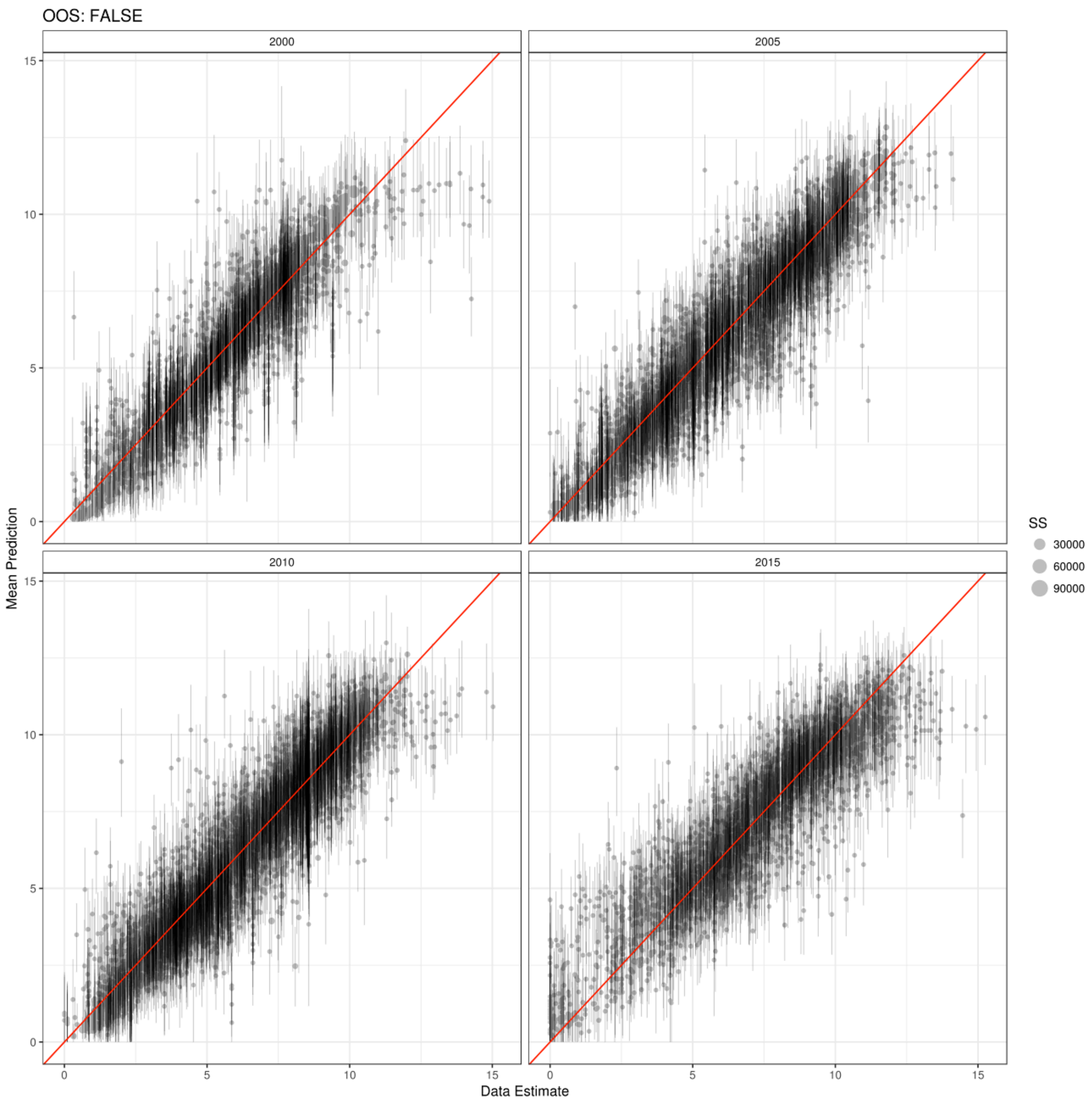

Supplementary Figure 19. **Education (men, ages 15-49) admin 2 aggregation.** Comparison of in-sample education predictions aggregated to admin 2 with 95% uncertainty intervals plotted against admin 2 aggregated data observations.

5.3.6 Education (men, ages 20-24) validation metrics

Supplementary Table 21. Predictive metrics for education (men, ages 20-24) aggregated to admin 0.
The out of sample (OOS) column indicates whether the metric was calculated using in-sample or out-of-sample predictions.

Year	OOS	Median SS	Mean err.	RMSE	Corr.	95% Cov.
**2000**	FALSE	2250	-0.010	0.157	0.997	0.967
**2005**	FALSE	1804	-0.037	0.283	0.996	0.973
**2010**	FALSE	2303	0.185	0.345	0.992	0.962
**2015**	FALSE	2308	-0.103	0.442	0.970	0.855

Supplementary Table 22. Predictive metrics for education (men, ages 20-24) aggregated to admin 1

Year	OOS	Median SS	Mean err.	RMSE	Corr.	95% Cov.
**2000**	FALSE	238	0.024	0.327	0.992	0.966
**2005**	FALSE	122	-0.038	0.396	0.993	0.975
**2010**	FALSE	142	0.185	0.422	0.990	0.962
**2015**	FALSE	156	-0.106	0.529	0.969	0.848

Supplementary Table 23. Predictive metrics for education (men, ages 20-24) aggregated to admin 2

Year	OOS	Median SS	Mean err.	RMSE	Corr.	95% Cov.
**2000**	FALSE	26	0.016	0.476	0.982	0.968
**2005**	FALSE	14	-0.038	0.590	0.985	0.973
**2010**	FALSE	17	0.184	0.618	0.978	0.962
**2015**	FALSE	11	-0.108	0.972	0.900	0.856

Supplementary Table 24. Predictive metrics for education (men, ages 20-24) aggregated to holdout units

Year	OOS	Median SS	Mean err.	RMSE	Corr.	95% Cov.
**2000**	FALSE	6	-0.154	1.065	0.891	0.988
**2005**	FALSE	5	-0.037	0.785	0.974	0.974
**2010**	FALSE	8	0.184	0.858	0.955	0.961
**2015**	FALSE	5	-0.104	0.998	0.884	0.852
**2000**	TRUE	6	-0.216	1.154	0.873	0.983
**2005**	TRUE	5	0.159	0.927	0.962	0.964
**2010**	TRUE	8	-0.033	1.038	0.929	0.935
**2015**	TRUE	5	-0.073	1.216	0.832	0.820

doi:10.1038/nature25761

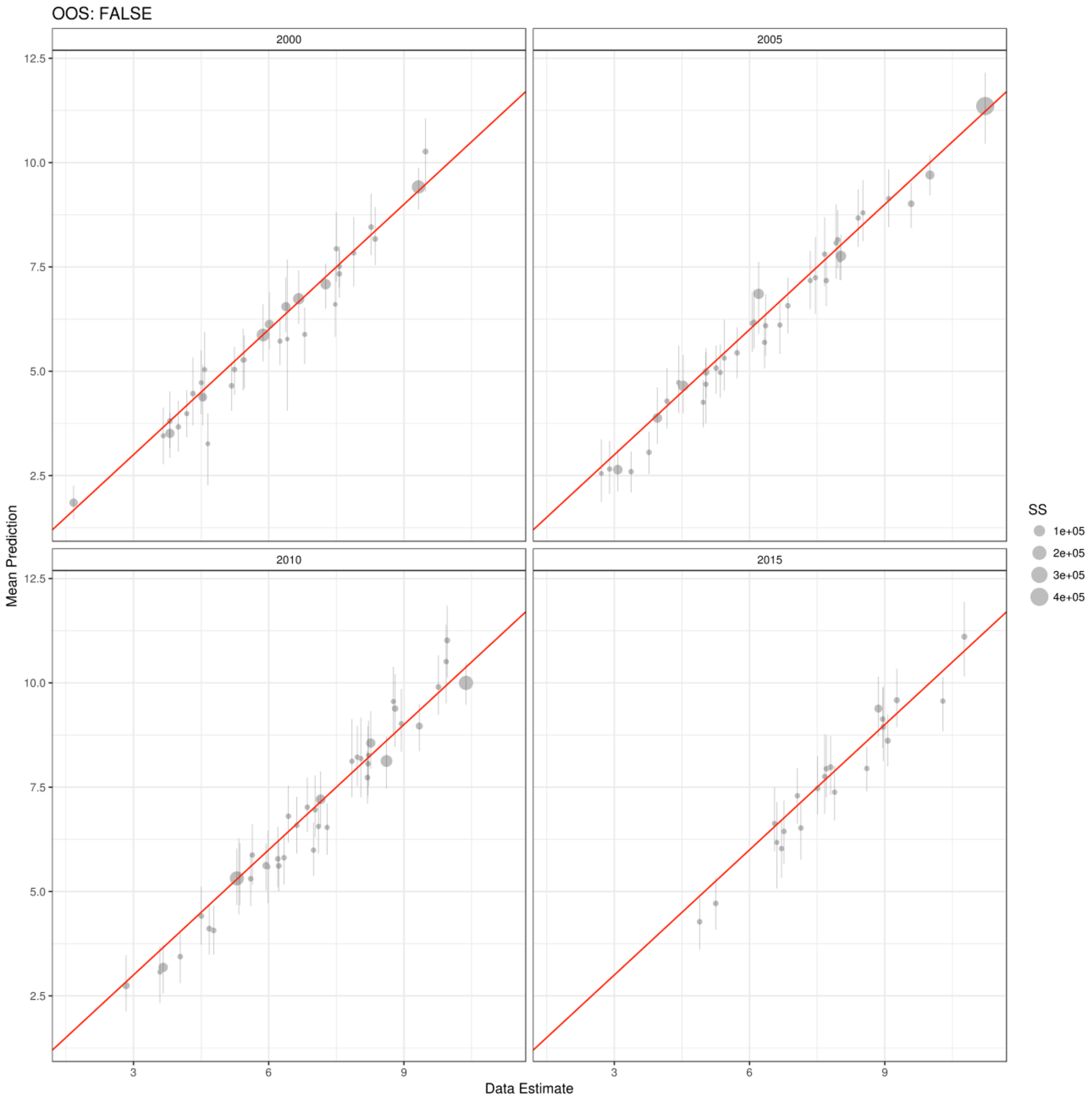

Supplementary Figure 20. Education (men, ages 20-24) admin 0 aggregation.
Comparison of in-sample education predictions aggregated to admin 0 with 95% uncertainty intervals plotted against admin 0 aggregated data observations.

doi:10.1038/nature25761

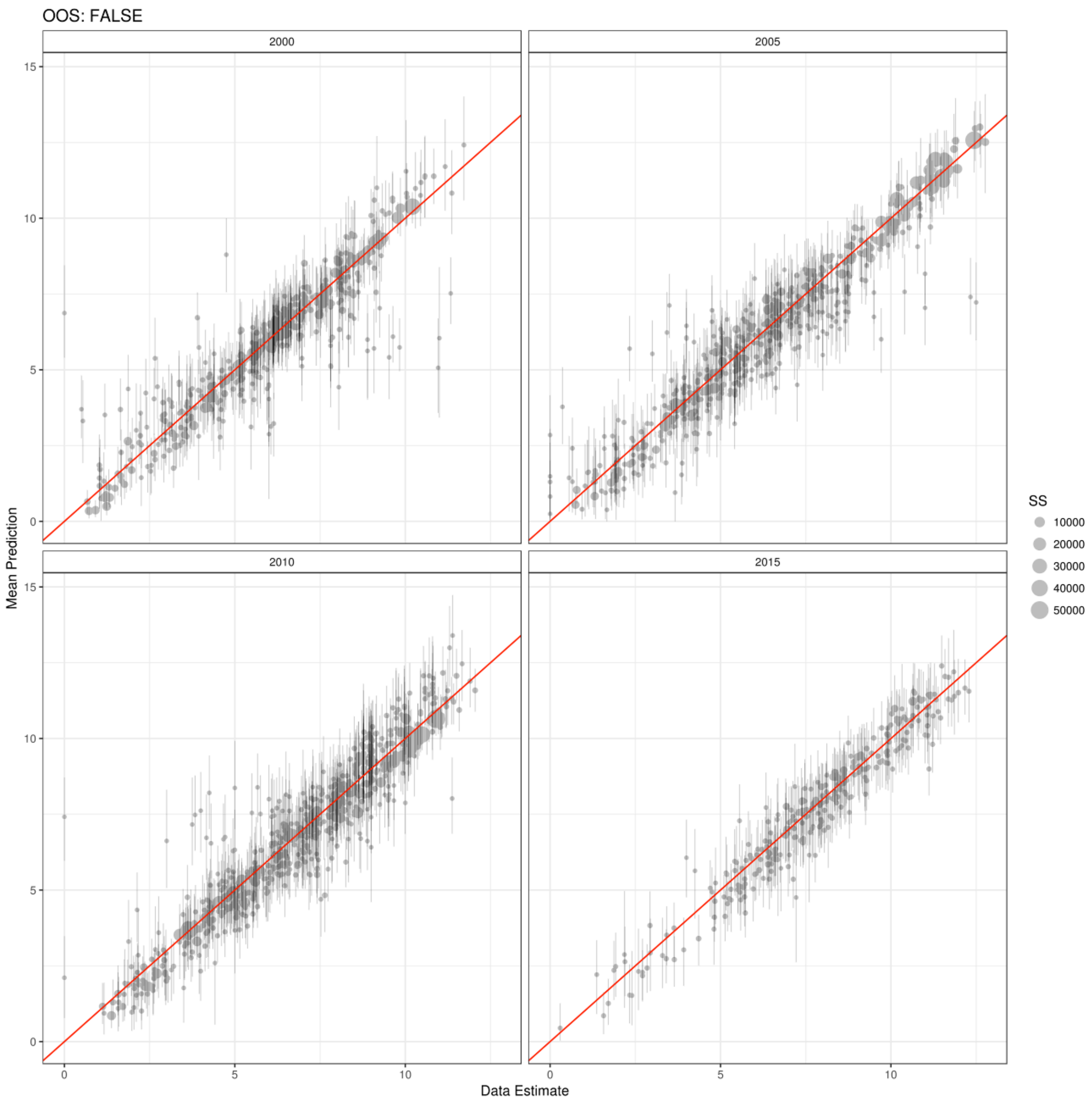

Supplementary Figure 21. Education (men, ages 20-24) admin 1 aggregation.
Comparison of in-sample education predictions aggregated to admin 1 with 95% uncertainty intervals plotted against admin 1 aggregated data observations.

doi:10.1038/nature25761

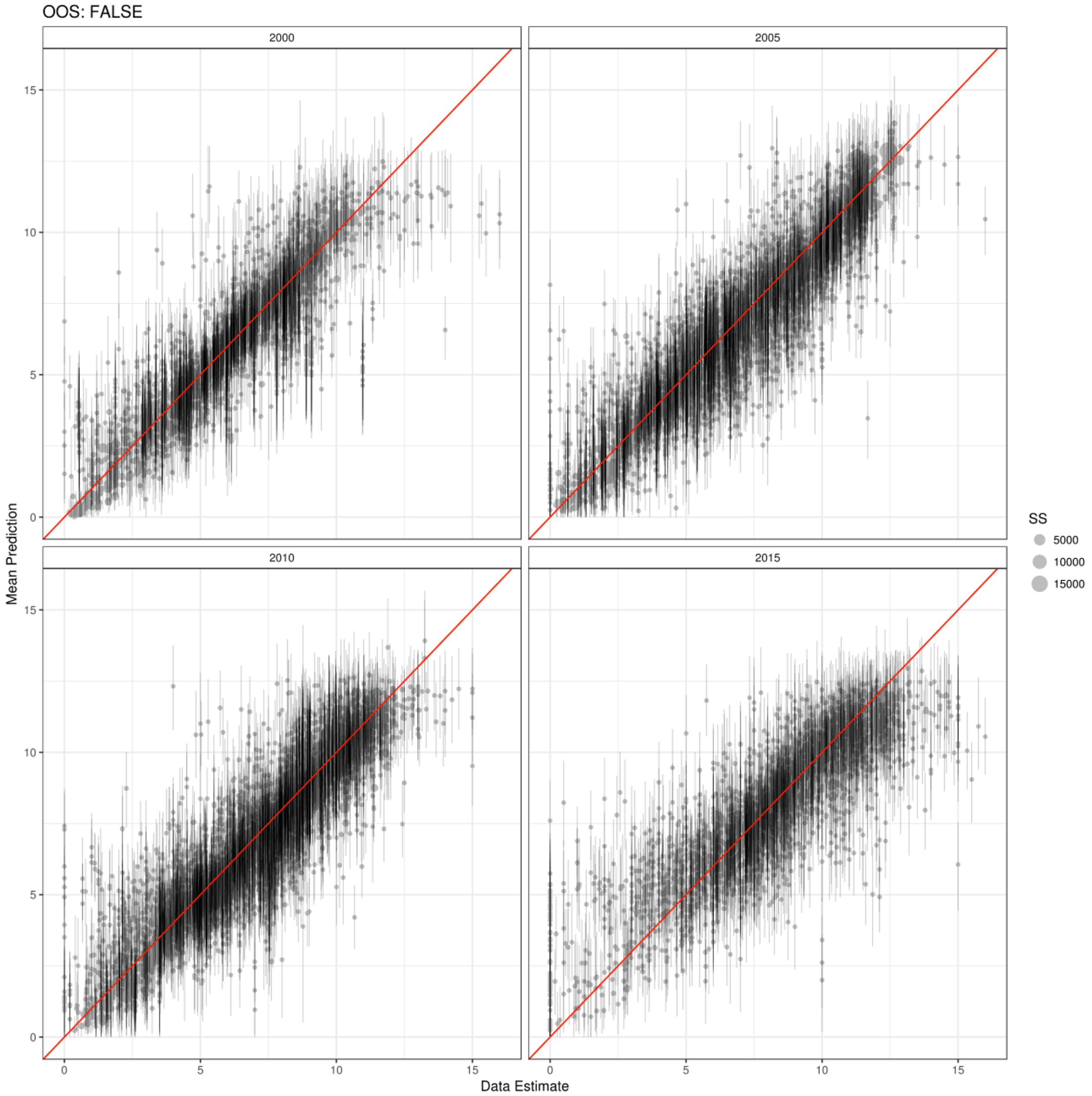

Supplementary Figure 22. Education (men, ages 20-24) admin 2 aggregation.
Comparison of in-sample education predictions aggregated to admin 2 with 95% uncertainty intervals plotted against admin 2 aggregated data observations.

5.3.7 Post estimation calibration to national estimates

In order to leverage national-level data included in GBD 2016, but outside the scope of our current geospatial modelling framework, and to ensure perfect calibration between these estimates and GBD 2016 national-level estimates, we performed a post hoc calibration to each of our 1,000 candidate maps.[32] For each posterior draw we calculated population-weighted pixel aggregations to a national level and compared these country-year estimates to the GBD 2016[32] country-years. We defined the raking factor to be the ratio between the GBD 2016[32] estimate and our current estimates. Finally, we multiplied each of our pixels in a country-year by its associated raking factor. This ensures perfect calibration between our geospatial estimates and GBD 2016[32] national-level estimates, while preserving our estimated within-country geospatial and temporal variation.

To allow comparison between our modelled estimates and the GBD 2016[32] national-level estimates to which they were calibrated, Supplementary Fig. 28-30 plot mean uncalibrated estimates from the model-based geostatistics (MBG) process aggregated to the national-level ("MBG mean") as compared to the GBD national estimates ("GBD mean") for all modelled years. The median raking factors for women 15-49, men 15-49, women 20-24, and men 20-24 were 0.926 (interquartile range: 0.794-1.084), 0.895 (IQR: 0.761-1.012), 1.036 (IQR: 0.798-1.031), and 1.053 (IQR: 0.861-1.233) respectively.

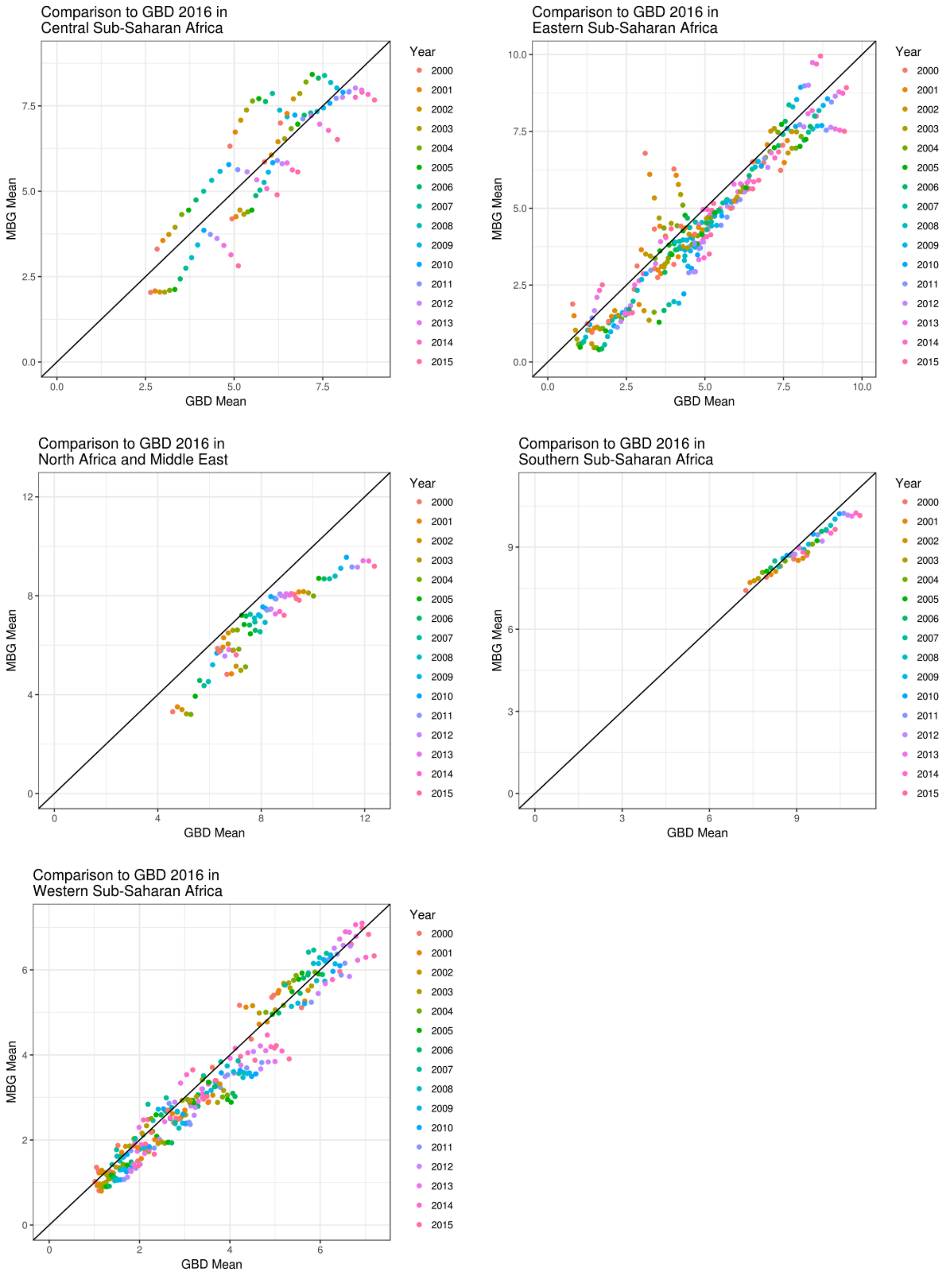

Supplementary Figure 23. Comparison of aggregated education (women, ages 15-49) MBG estimates to GBD 2016 education estimates.
Note that our models and GBD 2016 datasets overlap but are not identical.

doi:10.1038/nature25761

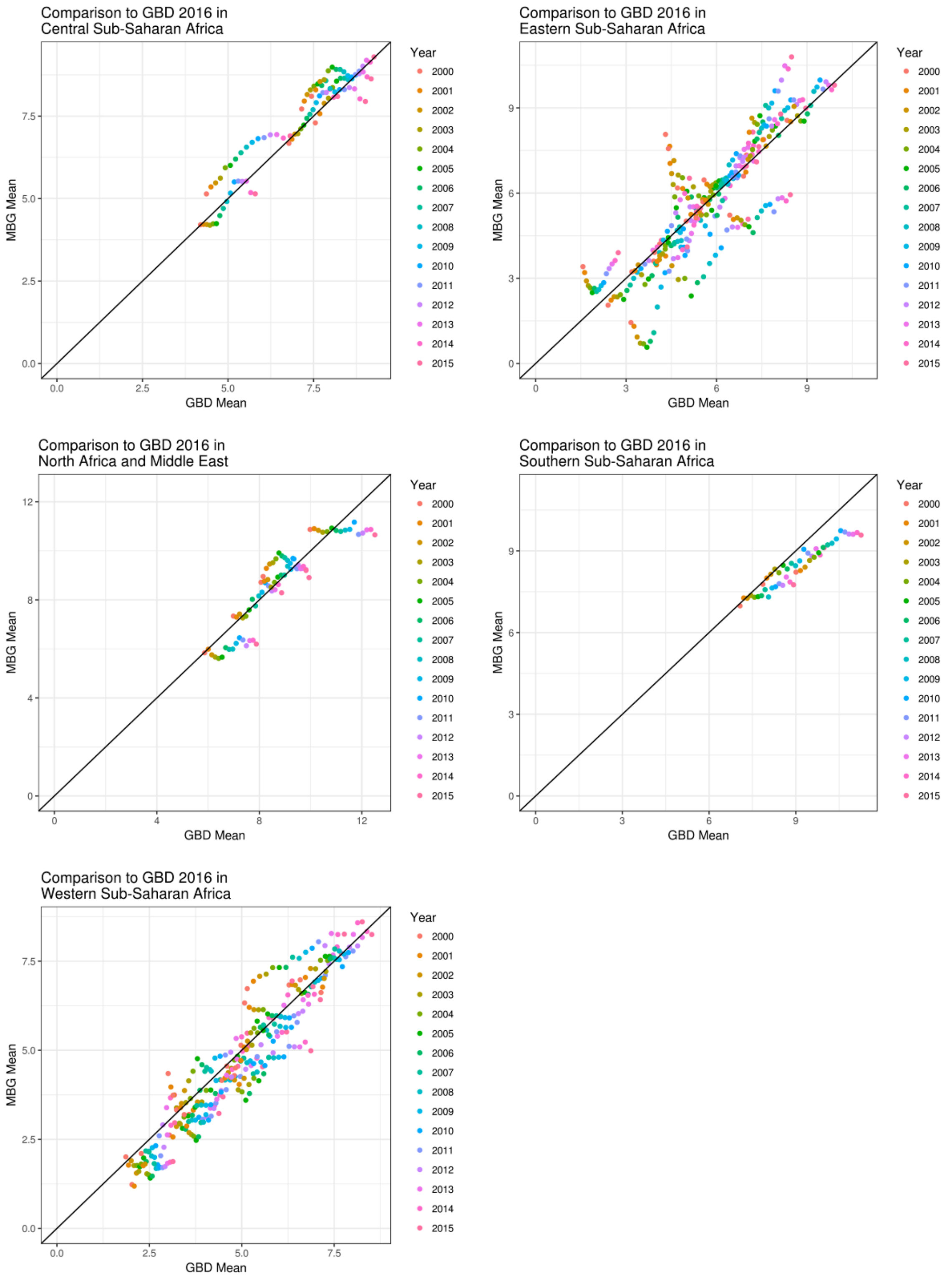

Supplementary Figure 24. Comparison of aggregated education (men, ages 15-49) MBG estimates to GBD 2016 education estimates.
Note that our models and GBD 2016 datasets overlap but are not identical.

doi:10.1038/nature25761

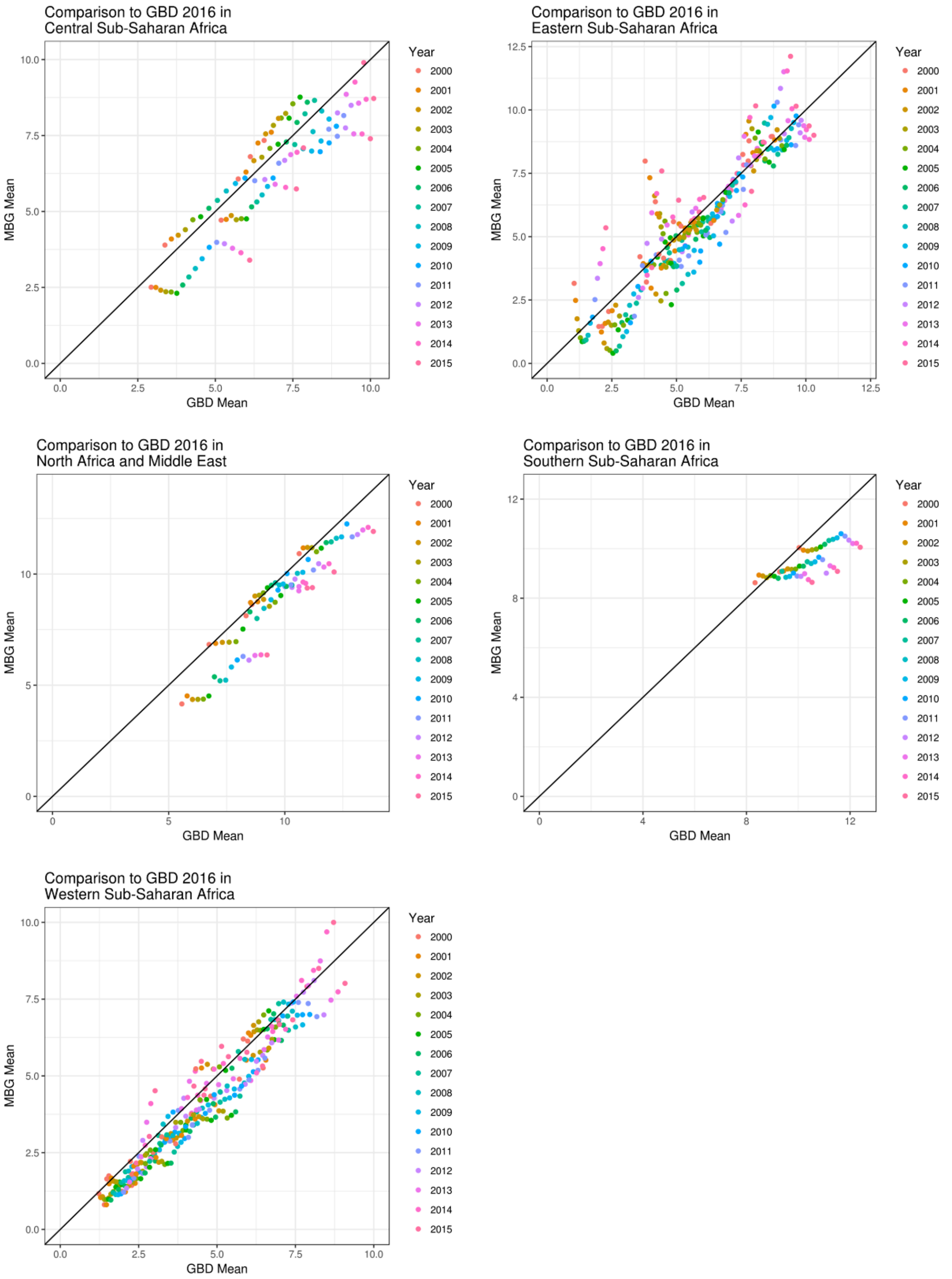

Supplementary Figure 25. Comparison of aggregated education (women, ages 20-24) MBG estimates to GBD 2016 education estimates.
Note that our models and GBD 2016 datasets overlap but are not identical.

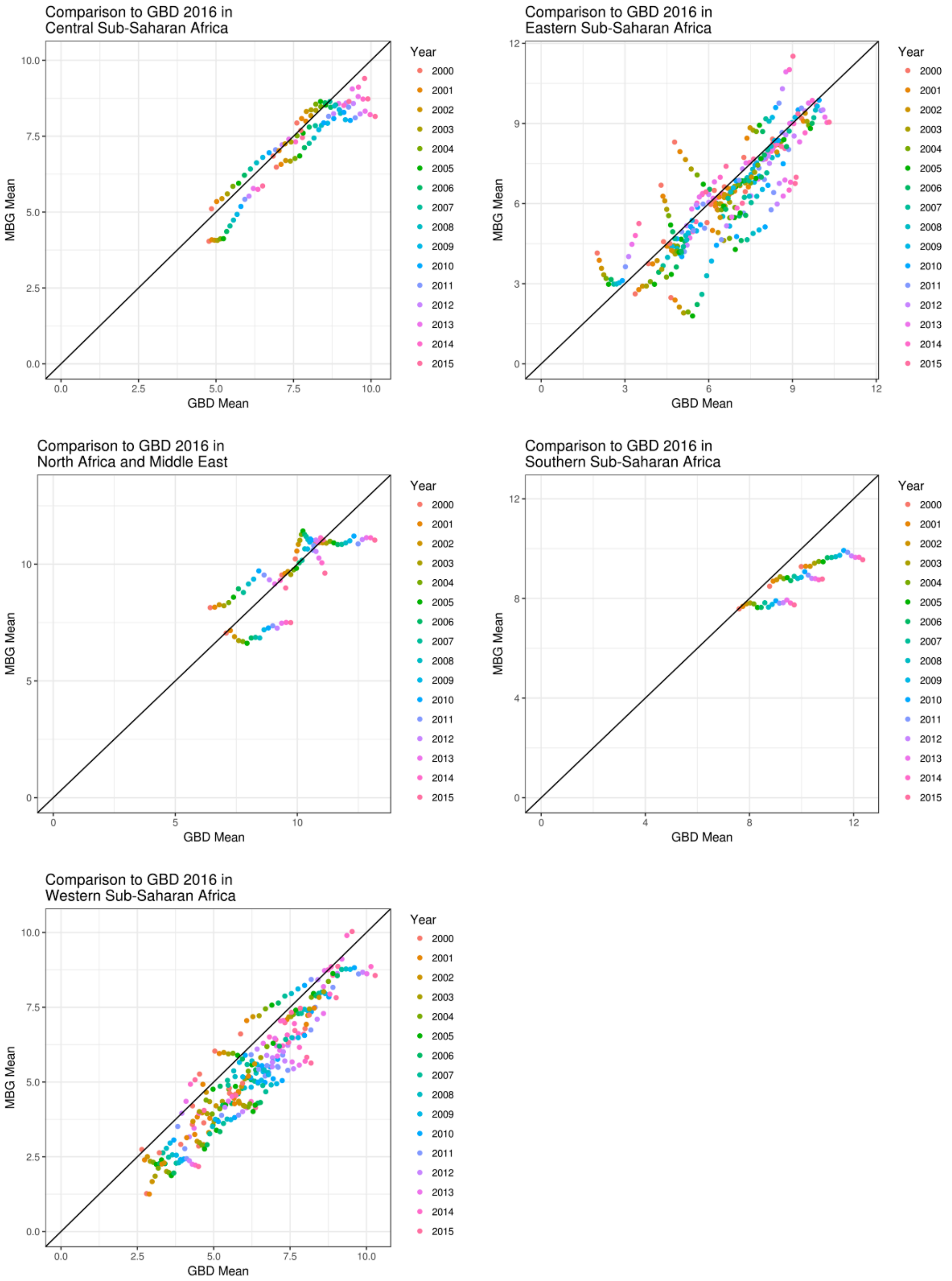

Supplementary Figure 26. Comparison of aggregated education (men, ages 20-24) MBG estimates to GBD 2016 education estimates.

Note that our models and GBD 2016 datasets overlap but are not identical.

5.3.8 Verification and comparison against other subnational education estimates

Estimates produced by MBG models were compared to raw estimates from the DHS series, each aggregated to the first subnational geographic subdivision. These results are presented below.

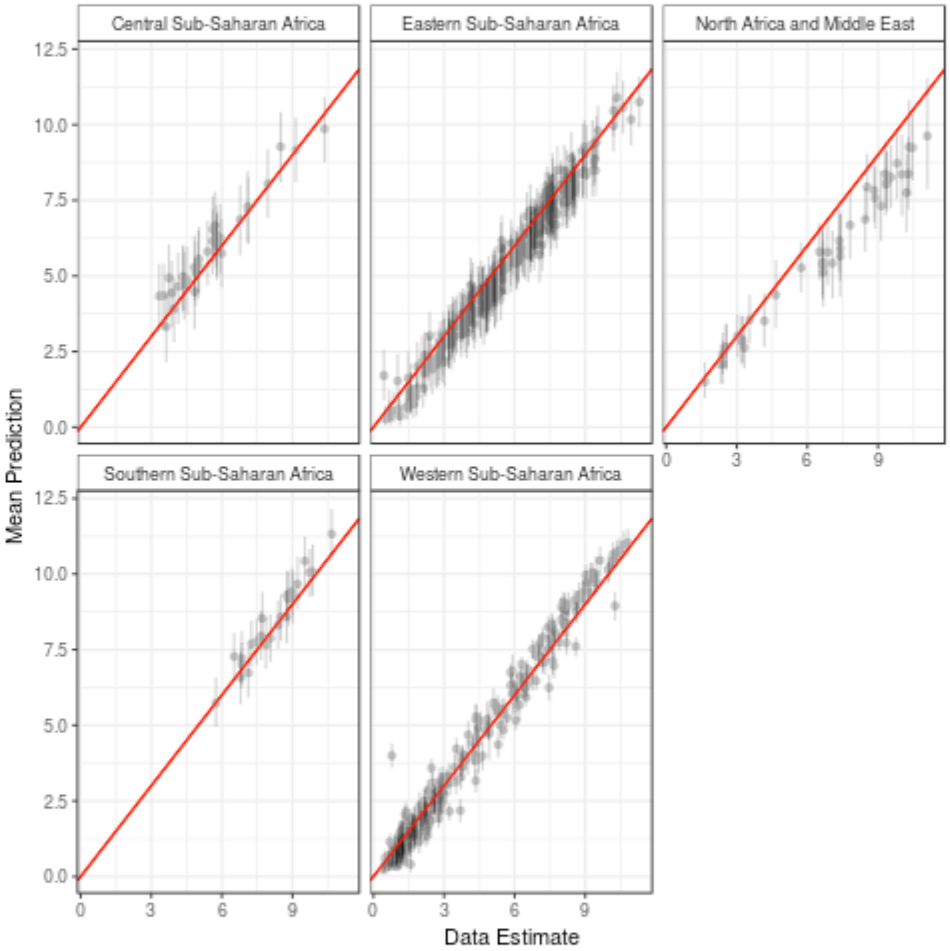

Supplementary Figure 27. Comparison of education (women, ages 15-49) MBG estimates aggregated to admin 1 to DHS admin 1 estimates.
95% uncertainty intervals are plotted along with the aggregated MBG estimates. Note that our model includes more data than just the DHS surveys.

doi:10.1038/nature25761

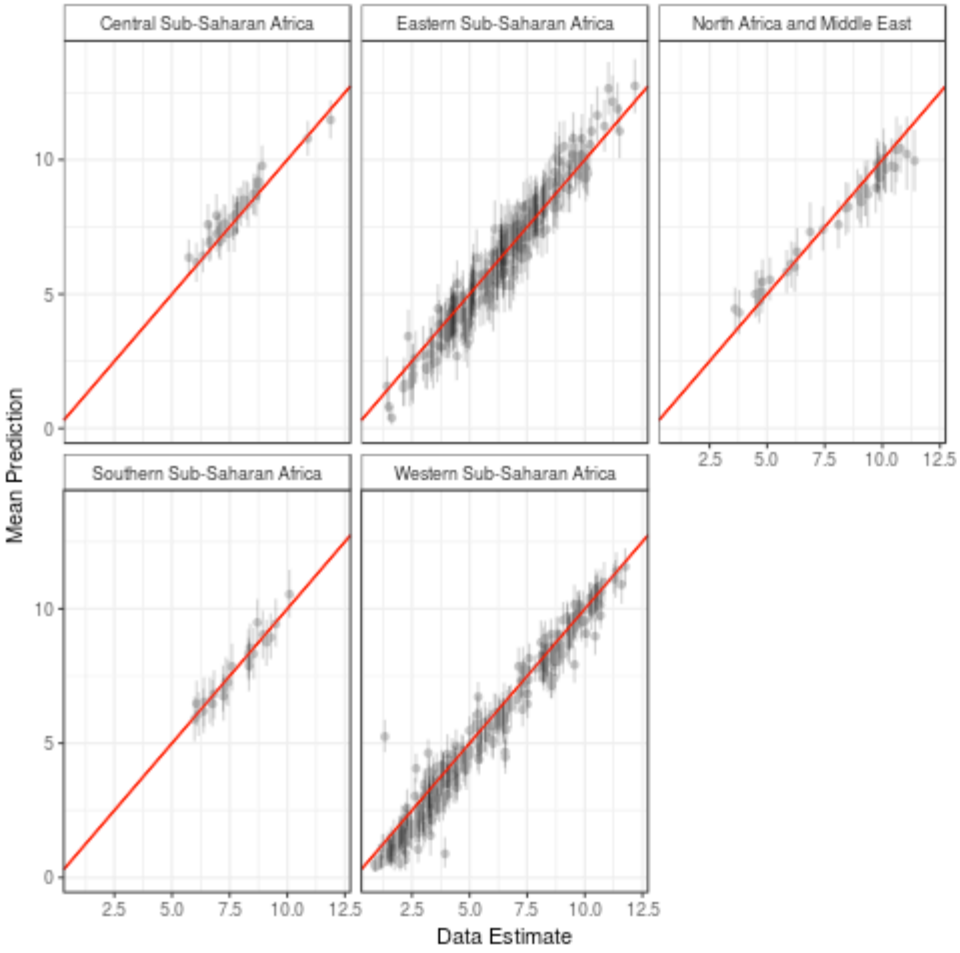

Supplementary Figure 28. Comparison of education (men, ages 15-49) MBG estimates aggregated to admin 1 to DHS admin 1 estimates.

doi:10.1038/nature25761

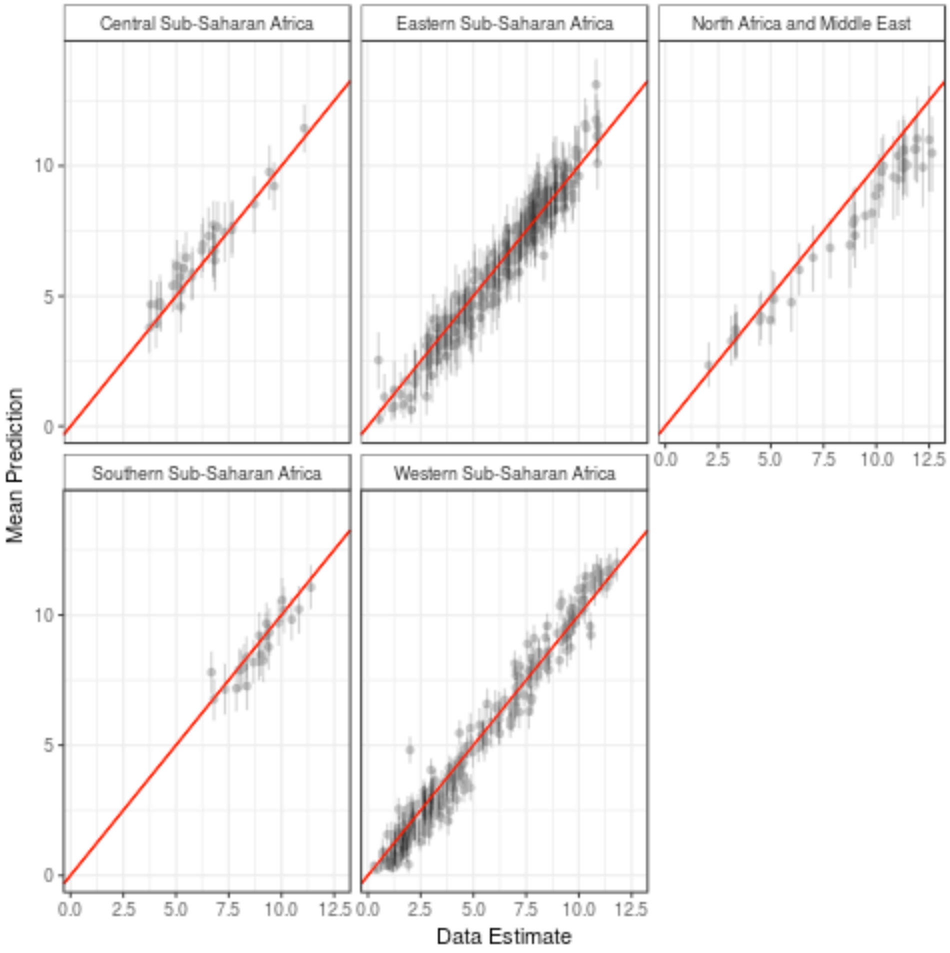

Supplementary Figure 29. Comparison of education (women, ages 20-24) MBG estimates aggregated to admin 1 to DHS admin 1 estimates.

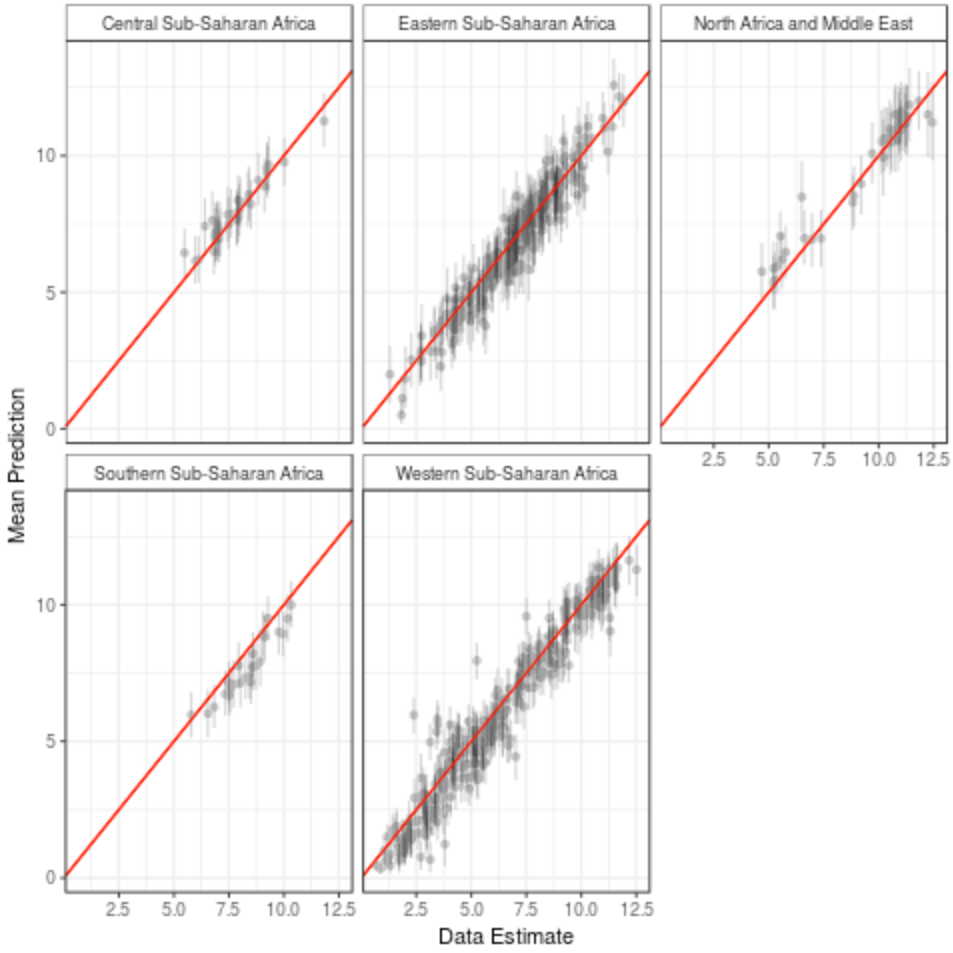

Supplementary Figure 30. Comparison of education (men, ages 20-24) MBG estimates aggregated to admin 1 to DHS admin 1 estimates.

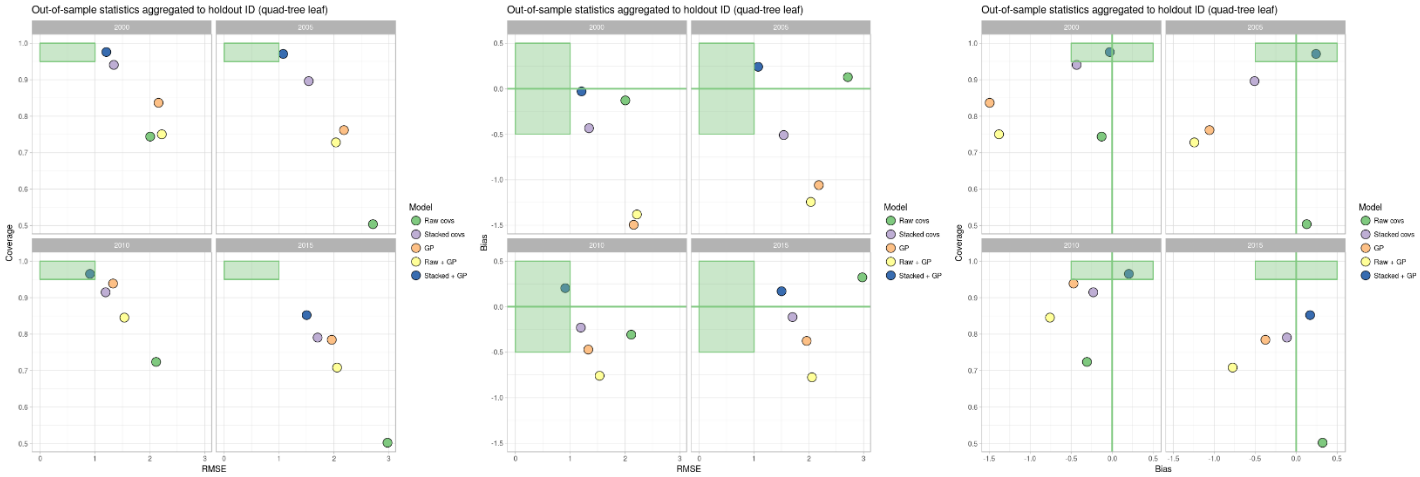

Supplementary Figure 31. Education (women 15-49) out-of-sample root-mean-squared-error (RMSE) and 95% coverage.

Fit statistics are aggregated by periods 2000-2002, 2003-2007, 2008-2012, and 2013-2015. “Covariates” is the INLA model fit with linear terms on all raw covariates; “Stacking” is fit with all stacking surfaces; “GP” is fit only with the space-time Gaussian process; “GP + covariates” is fit with linear terms on all raw covariates and the space-time Gaussian process; “GP + stacking” is fit with all stacking surfaces and the space-time Gaussian process. The green polygon highlights the area of desirable fit, defined for coverage (>95%), RMSE (0 to 1 year of education), and bias (-0.5 to 0.5 years of education).

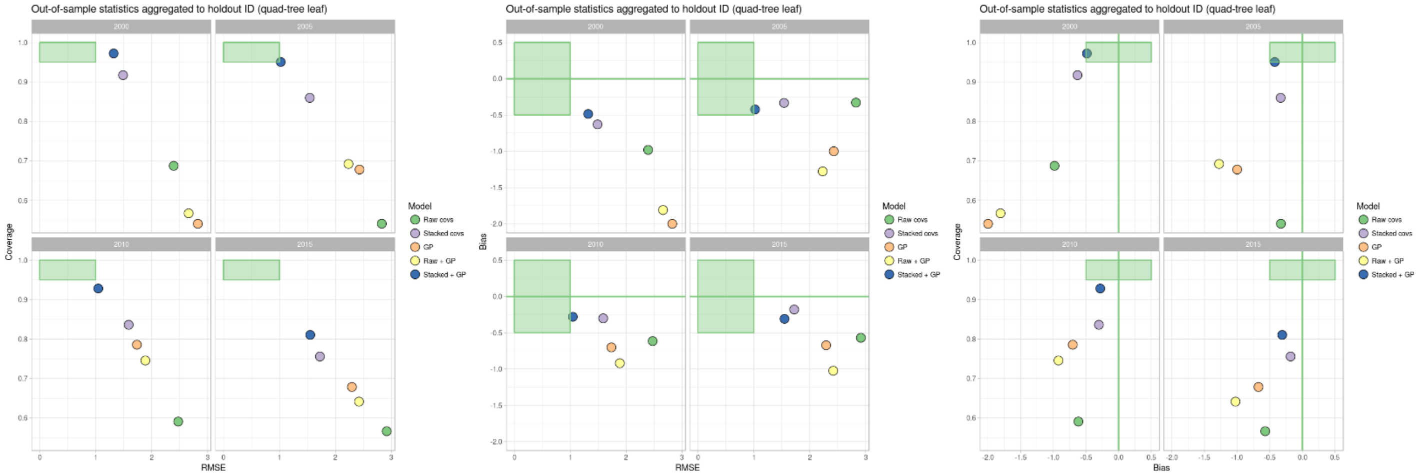

Supplementary Figure 32. Education (men 15-49) out-of-sample root-mean-squared-error (RMSE) and 95% coverage.

Fit statistics are aggregated by periods 2000-2002, 2003-2007, 2008-2012, and 2013-2015. “Covariates” is the INLA model fit with linear terms on all raw covariates; “Stacking” is fit with all stacking surfaces; “GP” is fit only with the space-time Gaussian process; “GP + covariates” is fit with linear terms on all raw covariates and the space-time Gaussian process; “GP + stacking” is fit with all stacking surfaces and the space-time Gaussian process. The green polygon highlights

the area of desirable fit, defined for coverage (>95%), RMSE (0 to 1 year of education), and bias (-0.5 to 0.5 years of education).

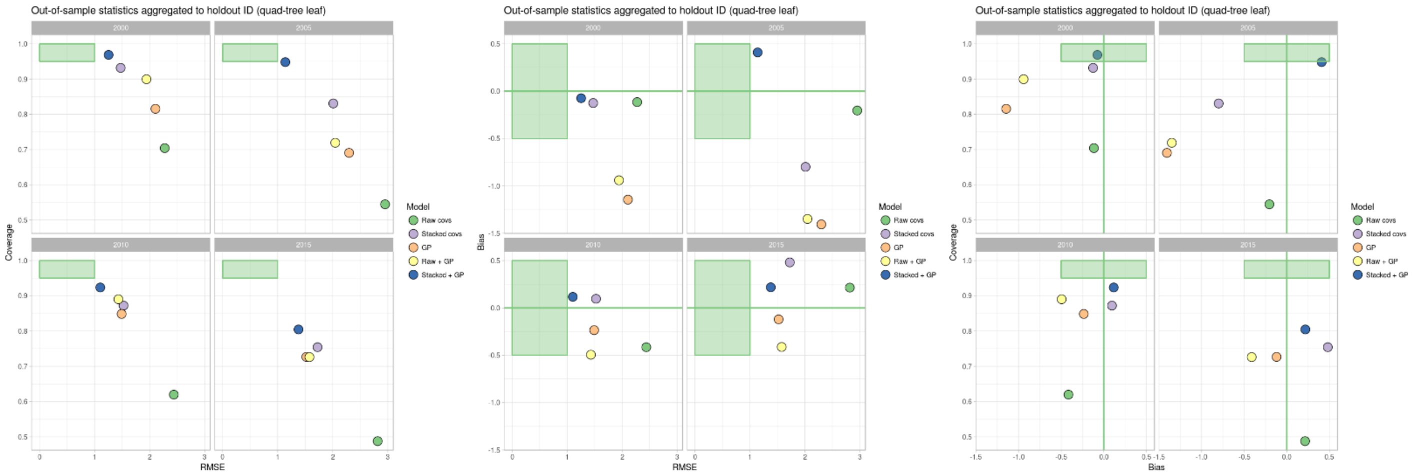

Supplementary Figure 33. Education (women 20-24) out-of-sample root-mean-squared-error (RMSE) and 95% coverage.
Fit statistics are aggregated by periods 2000-2002, 2003-2007, 2008-2012, and 2013-2015. “Covariates” is the INLA model fit with linear terms on all raw covariates; “Stacking” is fit with all stacking surfaces; “GP” is fit only with the space-time Gaussian process; “GP + covariates” is fit with linear terms on all raw covariates and the space-time Gaussian process; “GP + stacking” is fit with all stacking surfaces and the space-time Gaussian process. The green polygon highlights the area of desirable fit, defined for coverage (>95%), RMSE (0 to 1 year of education), and bias (-0.5 to 0.5 years of education).

doi:10.1038/nature25761

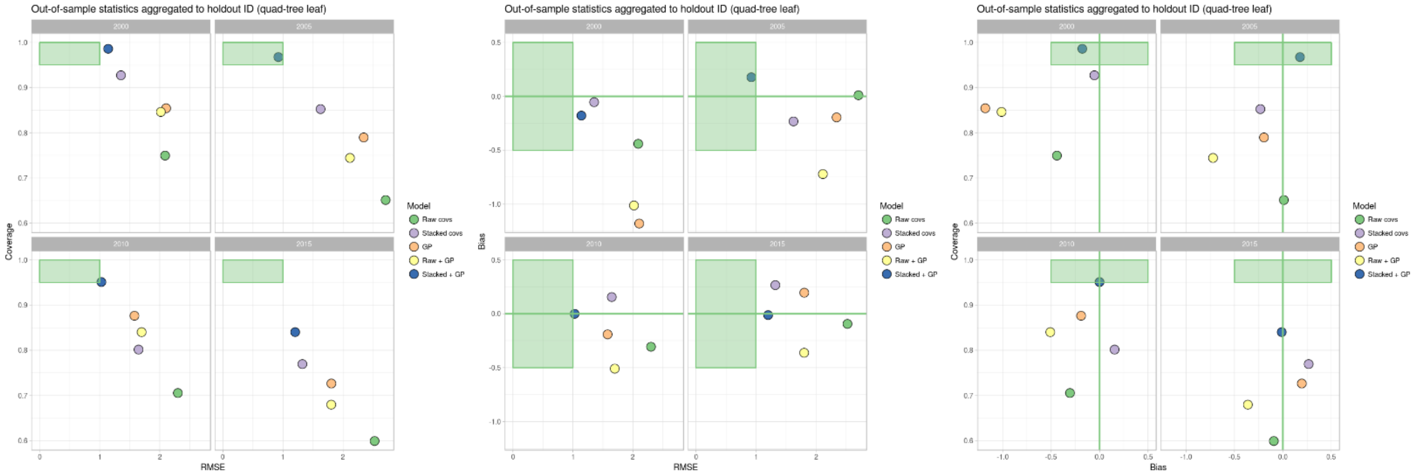

Supplementary Figure 34. **Education (men 20-24) out-of-sample root-mean-squared-error (RMSE) and 95% coverage.**

Fit statistics are aggregated by periods 2000-2002, 2003-2007, 2008-2012, and 2013-2015. “Covariates” is the INLA model fit with linear terms on all raw covariates; “Stacking” is fit with all stacking surfaces; “GP” is fit only with the space-time Gaussian process; “GP + covariates” is fit with linear terms on all raw covariates and the space-time Gaussian process; “GP + stacking” is fit with all stacking surfaces and the space-time Gaussian process. The green polygon highlights the area of desirable fit, defined for coverage (>95%), RMSE (0 to 1 year of education), and bias (-0.5 to 0.5 years of education).

6.0 Supplementary references

1. World Bank Group Education Strategy 2020. *Learning for All: Investing in People's Knowledge and Skills to Promote Development*. (2011).
2. UNESCO. *Aid to education is stagnating and not going to countries most in need*. (2017).
3. LeVine, R. A., LeVine, S., Schnell-Anzola, B., Rowe, M. L. & Dexter, E. *Literacy and MotheringHow Women's Schooling Changes the Lives of the World's Children*. (Oxford University Press, 2012). doi:10.1093/acprof:oso/9780195309829.001.0001
4. UNESCO. Is real progress being made in the equitable provision of education? #PISAresults | IIEP UNESCO.
5. Nilsson, M., Griggs, D. & Visbeck, M. Policy: Map the interactions between Sustainable Development Goals. *Nature* **534,** 320–322 (2016).
6. Hillman, A. L., Hillman, A. L., Jenkner, E. & International Monetary Fund. *Educating Children in Poor Countries*. (International Monetary Fund, 2004).
7. Kremer, M. Randomized Evaluations of Educational Programs in Developing Countries: Some Lessons. *The American Economic Review* **93,** 102–106
8. Friedman, T., Schwantner, U., Spink, J., Tabata, N. & Waters, C. Improving Quality Education and Children's Learning Outcomes and Effective Practices in the Eastern and Southern Africa Region Report for UNICEF ESARO b.
9. World Bank & International Center for Research on Women. Economic Impacts of Child Marriage. (2017).
10. UNESCO. *Accountability in Education: Meeting our Commitments*. *Global Education Monitoring Report 2017/18* (2017). doi:10.1017/CBO9781107415324.004
11. UNESCO. Education 2030. *World Educ. Forum 2015* (2015).
12. World Bank. Results-Based Financing (RBF) and Results in Education for All Children (REACH). (2017). Available at: http://www.worldbank.org/en/programs/reach. (Accessed: 2nd November 2017)
13. Gakidou, E., Cowling, K., Lozano, R. & Murray, C. J. Increased educational attainment and its effect on child mortality in 175 countries between 1970 and 2009: A systematic analysis. *Lancet* **376,** 959–974 (2010).
14. Pamuk, E., Fuchs, R. & Lutz, W. Cmparing Relative Effects of Education and Economic Resources on Infant Mortality in Developing Countries. *Popul. Dev. Rev.* **37,** 637–664 (2011).
15. Fuchs, R., Pamuk, E. & Lutz, W. Education or wealth: which matters more for reducing child mortality in developing countries? *Vienna Yearbook of Population Research* **8,** 175–199 (2010).
16. UNESCO. *Global Education Monitoring Report*. (2016).
17. Thomas Lumley. Survey analysis in R. (2014).

18. Gelman, A. Struggles with Survey Weighting and Regression Modeling 1. *Stat. Sci.* **22,** 153–164 (2007).

19. Lumley, T. in *Complex Surveys* 17–37 (John Wiley & Sons, Inc., 2010).

20. Kish, L. *Survey sampling*. (Wiley, 1995).

21. Golding, N., Burstein, R., Longbottom, J. & et al. Mapping under-5 and neonatal mortality in Africa, 2000–2015: a baseline analysis for the Sustainable Development Goals. *Lancet (In Press.* (2017).

22. Tatem, A. J. WorldPop, open data for spatial demography. *Sci. Data* **4,** 170004 (2017).

23. Osgood-Zimmerman, A. & Millear, A. Mapping child growth failure in Africa between 2000 and 2015. *Under Rev.* (2017).

24. Bhatt, S. *et al.* Improved prediction accuracy for disease risk mapping using Gaussian Process stacked generalisation. (2016).

25. Kassebaum, N. J. *et al.* Global, regional, and national disability-adjusted life-years (DALYs) for 315 diseases and injuries and healthy life expectancy (HALE), 1990–2015: a systematic analysis for the Global Burden of Disease Study 2015. *Lancet* **388,** 1603–1658 (2016).

26. Stein, M. L. *Interpolation of Spatial Data*. (Springer New York, 1999).

27. Waller, L. & Carlin, B. in *Handbook of Spatial Statistics* 217–243 (CRC Press, 2010).

28. Rue, H., Martino, S. & Chopin, N. Approximate Bayesian inference for latent Gaussian models by using integrated nested Laplace approximations. *J. R. Stat. Soc. Ser. B (Statistical Methodol.* **71,** 319–392 (2009).

29. Patil, A. P., Gething, P. W., Piel, F. B. & Hay, S. I. Bayesian geostatistics in health cartography: The perspective of malaria. *Trends Parasitol.* **27,** 246–253 (2011).

30. Roberts, D. R. *et al.* Cross-validation strategies for data with temporal, spatial, hierarchical, or phylogenetic structure. *Ecography (Cop.).* n/a-n/a (2017). doi:10.1111/ecog.02881

31. FAO. The Global Administrative Unit layers (GAUL): Technical Aspects.

32. GBD 2016 Risk Factors Collaborators. Global, regional, and national comparative risk assessment of 84 behavioural, environmental and occupational, and metabolic risks or clusters of risks, 1990-2016: a systematic analysis for the Global Burden of Disease Study 2016. *Lancet (In Rev.* (2017).
